# Supplementary material for: Insights into the Role of a Cardiomyopathy-Causing Genetic Variant in ACTN2
Source: Cells. 2023 Feb 24;12(5):721. doi: 10.3390/cells12050721 (PMC10001372; doi:10.3390/cells12050721)
Supplement: Supplementary file 1 [file cells-12-00721-s001.zip › supplementary.pdf]

Supplementary Materials

Supplementary Materials and Methods

Generation of the mice and genotyping

The p.Met228Thr variant was introduced into the orthologous position in the mouse *Actn2* gene using CRISPR-Cas9 mediated homology-directed repair in mouse embryonic stem cells. A target site for CRISPR mutagenesis (5'- CTTGGATATCCCTAAGATGT-3'), with minimal predicted off target sites (http://crispor.tefor.net) was selected, with the predicted nuclease cut site overlapping the Met-228 codon. Two complementary oligos encoding this CRISPR target were annealed and cloned into the BbsI site of pX330-Puro, encoding a CAG-Cas9 expression cassette and a U6-promoter driven single guide-RNA (sgRNA) scaffold, together with a puromycin resistance cassette. A 139 nt single-stranded-oligodeoxynucleotide (ssODN), serving as a homology repair template, was synthesized (Eurogentec, Seraing, Belgium). This contained the desired p.Met228Thr change and three silent base pair changes to introduce a *de novo* HgaI restriction site to facilitate the genotyping of the recombinant allele. Mouse C57BL/6N embryonic stem cells (ES, JM8F6) were electroporated with 5 µg of the pX330-Puro plasmid and 200 pmoles of the ssODN using the Neon transfection system (Life Technologies, Carlsbad, CA, USA, 3 x 1400V, 10ms). After 24 hours, 48 hours of selection in 600 ng/µl puromcyin was applied, and resistant ES cells were clonally expanded and genotyped using primers (5'-CACTCGTGTGTTAAGGGGCA-3' and 5'-CTGCATGGGTCCTGTGTTTC-3') to amplify the

target region. Successful targeting was first established by HgaI digestion, followed by confirmation by Sanger sequencing. ES cell clones harbouring the *Actn2* p.Met228Thr mutation were microinjected into albino C57BL/6J blastocysts. The resulting chimeras were bred with wild-type C57BL/6J mice (Envigo, London, UK) and germline transmission of the p.Met228Thr *Actn2* allele confirmed.

Animals were genotyped for *Actn2* p.Met228Thr mutation and a spontaneous genetic variant in the *Nnt* gene, occurring in C57BL/6J sub-strains [14] using Transnetyx services (Cordova, TN, USA). All animals of the colony were homozygous for the genetic variant in *Nnt*.

Ultrasound echocardiography

General anaesthesia was initially induced using 4 % isoflurane by inhalation (Piramal Critical Care) in an anaesthesia chamber. Animals were then placed onto a heated platform, and the anaesthesia was maintained continuously with the usage of 1-1.5 % isoflurane. Body temperature of the animals was maintained at 37 ºC, and echocardiography was performed with a 22 to 55 MHz linear array transducer using the Vevo 2100 ultrasound system (Visualsonics, Toronto, Canada). Data acquisition was normally completed within 30 min of anaesthesia, followed by offline analysis blinded to the genotype.

Embryo collection and fixation, Theiler staging

Animals were timed mated and noon on plug date was designated E0.5. At E15.5, pregnant animals were sacrificed and the uterus containing embryos was removed and placed into ice cold PBS with 5 mM EDTA. Embryos were dissected free of uterine tissues and yolk sac in fresh PBS with 5 mM EDTA. Umbilical vessels were cleanly cut to allow blood to drain from the embryo.

Once embryos were drained of blood, hearts were either dissected and flash frozen in liquid nitrogen or, for High Resolution Episcopic Microscopy (HREM) and wholemount staining, the whole embryo was placed into 4 % paraformaldehyde (PFA) for 24 hours at 4°C before being washed in PBS and placed in fresh PBS at 4°C.

For Theiler staging, PFA-fixed front limbs were examined under an inverted stereoscope (TI3000 Ergo stereoscope, Leica, Mannheim, Germany) and classified according to [16].

Proteasomal activity assays

Chymotrypsin-like, Caspase-like and Trypsin-like activity assays were performed as previously described using commercially available indirect enzyme based luminescent assay kits (Promega, Madison, WI, USA) [17]. Frozen embryonic hearts from E15.5 were lysed using tissue disruption and ice-cold hypotonic buffer (10mM Tris-HCL pH 7.5, 5mM $MgCl_2$, 5mM ATP). Protein concentration was estimated using Pierce™ Rapid Gold BCA protein assay kit (Thermo Fisher Scientific, Waltham, MA, USA). Lysates were then diluted to 0.14 mg/mL in hypotonic buffer and added to white-walled 96-well plate. Suc-LLVY-Glo™, Z-LRR-Glo™ and Z-nLPnLD-Glo™ were added separately to samples to measure Chymotrypsin-like activity, Trypsin-like activity and Caspase-like activity respectively. Following incubation at room temperature for 60 minutes, the luminescent signal intensity was measured using GloMax® Luminometer (Promega, Madison, WI, USA).

High Resolution Episcopic Microscopy

Tissue and data processing for HREM was performed as previously described [18]. Fixed embryos were washed extensively with PBS, then a methanol series was performed (10%, 20%, 30%, 40%, 50%, 60%, 70%, 80%, 90%, 95%, 100%, 100%) for 2 hours each prior to infiltration overnight in a 50:50 mixture of 100% methanol and JB-4 resin (00226-1, Polysciences), including 0.275g/100ml EosinB (Sigma Aldrich, St. Louis, MO, USA). Embryos were washed in the JB-4 infiltration solution for 1 hour prior to incubation for 1 week in fresh JB-4 solution. Embryos were sectioned at 3 µm using an optical HREM microscope (Indigo Scientific, Baldock, UK). Data was analysed using Horos 3.3.6 (https:// horosproject.org, accessed 23 Feb 2023) to calculate diameter of aortic and pulmonary trunk vessel segments; and myocardial wall thickness, and Amira for Life & Biomedical Sciences version 2019.4 (Thermo Fisher Scientific, Waltham, MA, USA) to generate precise

reconstructions and quantify volumes of the ventricles; ventricular lumens; atria; aortic and pulmonary semilunar valves; aortic arch; and pulmonary trunk.

Immunofluorescence staining on cryosections

Staining of cryosections of skeletal muscle was performed as described [15].

Hearts from E15.5 embryos were removed and embedded in Tissue-Tek OCT compound. After embedding, 7 micron-thick cryo-sections were cut and mounted onto positively charged glass slide followed by drying at room temperature for 30 minutes. Slides were stored at -80 before undergoing immunofluorescent staining.

Slide were placed into ice-cold acetone for 10 minutes followed by three washes in PBS. Slides were then incubated with PBS- 0.01% Triton-x 100 for 20 minutes at room temperature before undergoing incubation with mouse on mouse blocking reagent (2B Scientific Limited) for 60 minutes. This was then followed by incubation with antibodies against Phospho-Histone H3 (Thermo Fisher Scientific, Waltham, MA, USA) and alpha-actinin (see Table S9) overnight at 4 degrees. Sections were washed 3 times with PBS and incubated with Alexa Fluor labelled antibodies (Thermo Fisher Scientific, Waltham, MA, USA) against the relevant species of the primary antibodies. DAPI stain was also included to identify nuclei. Slides were incubated for two hours at room temperature before undergoing three more washes with PBS and subsequent mounting aquamount media and cover slip. Stained sections were analysed using Zeiss LSM 780 confocal microscope and ImageJ version 1.53a.

Mass spectrometry for identification

An excised gel sample was digested with trypsin (Promega, Madison, WI, USA) and analysed by nano-UPLC–MS/MS using a Dionex Ultimate 3000 coupled online to an Orbitrap Fusion Lumos mass spectrometer (Thermo Scientific) as described [20]. Raw data was analysed using PEAKS software (v 8.5). Mass tolerances were set to 10 ppm (precursor) and 0.05 Da (fragment). We allowed up to 3 missed cleavage sites under tryptic digest restrictions and set Carbamidomethylation (Cys) as fixed

modification while Oxidation (M) and Deamidation (Asn, Gln) were set as variable modifications. Data were searched against the reference proteome from *Mus musculus*, (Uniprot, after addition of Actn2 p.Met228Thr).

Proteomics

Wildtype and homozygous (n=6 per group) ventricular samples previously collected from embryos at E15.5 were homogenized using lysis solution provided in EasyPepTM mini MS sample prep kit (ThermoFisher Scientific, Waltham, MA, USA). After removal of insoluble material by centrifugation, total protein concentration was determined using PierceTM Rapid Gold BCA protein assay kit. Equal amounts of protein (100 ug) were reduced and alkylated using solutions provided in EasyPepTM mini MS sample prep kit following manufacturer's protocol.

Peptides were labelled with Tandem Mass Tag (TMTPro 16 plex, ThermoFisher Scientific, Waltham, MA, USA) dissolved in 100% acetonitrile and incubated for 1 hour at room temperature. Each reaction was quenched using 5% hydroxylamine, 20% formic acid solution. Resulting solution was then cleaned using peptide clean up columns according to manufacturer's protocol for EasyPep mini MS sample preparation.

Once labelled, the samples were then pooled and dried using SpeedVac and resuspended in 0.1% aqueous formic acid. A final clean up and drying step was performed using ZipTip-uC18 tips (Merck Millipore, Berlington, MA, USA) to remove any final unwanted solvents and excess labelling reagents. Peptides underwent a final drying step using SpeedVac and were resuspended in formic acid solution.

Mass spectroscopy was carried out by the University of Birmingham Advanced Mass Spectrometry Facility. UltiMate® 3000 HPLC series (Dionex, Sunnyvale, CA USA) was used for peptide concentration and separation. Samples were trapped on uPrecolumn Cartridge, Acclaim PepMap 100 C18, 5 um, 100A 300um i.d. x 5mm (Dionex, Sunnyvale, CA USA) and separated in Nano Series™ Standard Columns 75 µm i.d. x 15 cm, packed with C18 PepMap100, 3 µm, 100Å (Dionex, Sunnyvale, CA USA). The gradient used was from 3.2% to 24% solvent B (0.1% formic acid in acetonitrile) for 30 mins and then

increased to 60% in 15 min. The column was then washed with 80% solvent B for 15mins and equilibrated with 3.2% solvent B for another 15 mins. The total run time was 75 mins. Peptides were eluted directly (~ 350 nL min-1) via a Triversa Nanomate nanospray source (Advion Biosciences, NY) into a QExactive HF (QEHF) mass spectrometer (Thermo Fisher Scientific, Waltham, MA, USA). The data-dependent scanning acquisition was controlled by Xcalibur 4.0 software. The mass spectrometer alternated between a full FT-MS scan (m/z 375 – 1600) and subsequent high energy collision dissociation (HCD) MS/MS scans of the 20 most abundant ions. Survey scans were acquired in the QEHF with a resolution of 120 000 at m/z 200 and automatic gain control (AGC) 3x106. Precursor ions were fragmented in HCD MS/MS with resolution set up at 60,000 and a normalized collision energy of 32. ACG target for HCD MS/MS was 1x105. The width of the precursor isolation window was 1.2 m/z and only multiply-charged precursor ions were selected for MS/MS. Spectra were acquired for 60 mins with dynamic exclusion time of 20s.

For data analysis, the MS and MS/MS scans were searched against Uniprot database using Proteome Discoverer 2.2 (ThermoFisher Scientific, Waltham, MA, USA) with a 5 % false discovery rate (FDR) criteria. Oxidation (M) and N-terminal acetylation are set as variable modifications, cysteine carbamidomethylation and TMT 16-plex on lysine and N-terminal as fixed modifications. The precursor mass tolerance was 10 ppm and the MS/MS mass tolerance was 0.02 Da. The Quan method was set for reporter ions quantification with HCD and MS2. For the reporter ion abundance only unique peptides were considered for the quantification.

Electron microscopy

Embryos were generated as described above. Dissection of whole hearts was carried out in fresh ice-cold PBS with 5mM EDTA. Once cleaned of surrounding tissues, hearts were briefly flushed with ice-cold PBS via the aorta. Hearts were fixed in 4% PFA for 15 minutes before being transferred to 2.5% glutaraldehyde/2% PFA for two hours at room temperature then 3 hours at 4°C. Subsequently hearts were stored in 0.05% glutaraldehyde at 4°C.

Fixed hearts were briefly washed with fresh PBS and further dissected to remove the atria and blood vessels leaving the ventricles. This portion was cut crossways to give a small tip

fragment and a figure of eight fraction with most of the ventricular walls. These were further fixed in 1% osmium, dehydrated in ethanol, and embedded in Araldite. Before embedding the larger fragment was divided into three parts, the left and right ventricular walls and the septum. 70nm sections were stained with Uranyless heavy metal stain followed by Pb Citrate (both Labtech International Ltd, Heathfield, UK). The sections were viewed in a JEOL 1400 electron microscope in the Centre for Ultrastructural Imaging, KCL (London, UK).

Image analysis

ImageJ version 1.53a was used for co-localisation analysis and densitometry of Western blots. The ‘JACoP’ plugin (https://imagej.net/plugins/jacop, accessed on 22 Feb 2023) was used to calculate a correlation coefficient. The ImageJ plugin ‘Colocalisation finder’ (http://punias.free.fr/ImageJ/colocalization-finder.html, accessed on 22 Feb 2023) was used to generate cytofluorograms to visualise colocalisation.

Cell Profiler 4.2.1. was used for nuclear assessment [23]. Sarcomere length was analysed as described [24], using MatLab (version 2021a) and the script ‘ZLineDetection’ (https://github.com/Cardiovascular-Modeling-Laboratory/zlineDetection, accessed on 22 Feb 2023).

Statistics

All values are given as mean ± standard error of mean (SEM). To compare two unpaired sample groups, data was tested for normality using the Kolmogorov-Smirnov test. Normally distributed data was analysed by Student's *t*-test, and data that was not, was analysed by Mann-Whitney U-test. Deviation from expected Mendelian ratios and Theiler stages were assessed with Chi-square test. For comparison of three groups with normally distributed data, one-way ANOVA followed by Tukey’s post-hoc test was used. For image analysis of wholemount staining, nested ANOVA was employed allowing to consider the number of measurements from each heart. Fisher’s exact test was used to test occurrence of VSD. All statistical analyses were performed with GraphPad Prism 9.3.1.

Annotations used: * $p < 0.05$, ** $p < 0.01$, *** $p < 0.001$, **** $p < 0.0001$ versus WT, otherwise considered not significant ($p > 0.05$); n indicates number of animals in each group.

Supplementary Figures and Tables

Figure S1. Confirmation of the Actn2 p.Met228Thr change at protein level by proteomics. A total protein sample from an *Actn2* p.Met228Thr Het heart was separated by SDS-PAGE and the alpha-actinin 2 band (~95-105 kD) excised and subjected to mass spectrometry after trypic digest. Peptides were searched against a database into which Actn2 p.Met228Thr was added. (A) A peptide containing threonine at position 228 is indicated by an arrow. (B) Sequencing by MS/MS confirms the threonine at this position (arrow).

A

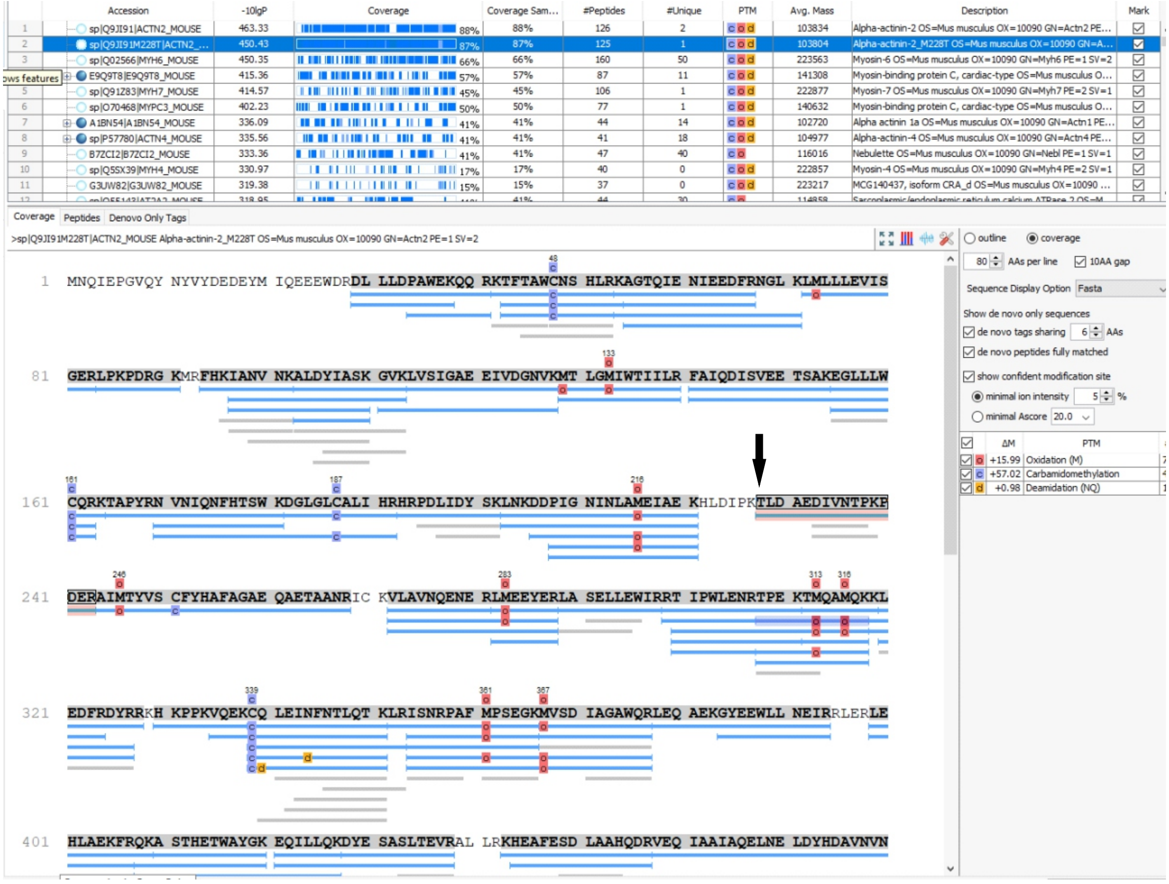

Accession
-10lgP
Coverage
#Peptides
#Unique
PTM
Avg. Mass
Description
Mark
Coverage
Peptides
Denovo Only Tags
outline
coverage
Sequence Display Option
Show de novo only sequences
show confident modification site
+15.99 Oxidation (M)
+57.02 Carbamidomethylation
+0.98 Deamidation (NQ)

B

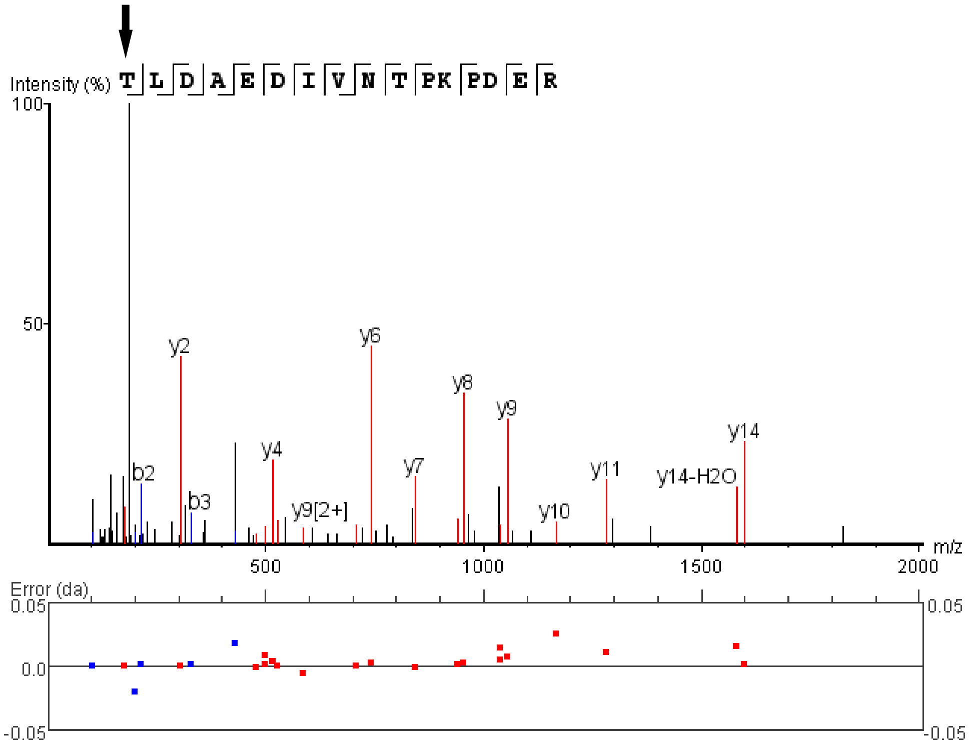

Intensity (%)
T L D A E D I V N T PK PD E R
100
50
y2
y6
y8
y9
y4
y14
b2
y7
y11
y14-H2O
b3
y9[2+]
y10
500
1000
1500
2000
m/z
Error (da)
0.05
0.0
-0.05

Figure S2. (A) Targeted assessment of transcriptional changes by qPCR in young male WT (white) and Het hearts (blue) for transcripts related to heart failure (fetal gene programme). Age: WT 99 ±1 d; Het 100 ±1 d; n = 6 per group. (B) Same analysis in mature female WT (white) and Het (green) hearts. Age: WT 250 ± 7 d, Het 254 ± 7 d; n = 6 per group. (C) The same mature female hearts were assessed for a panel related to hypertrophic signalling. All measurements are normalised to *Gapdh*. The only significant change observed was for *Ankrd2* in panel C (1.0 ± 0.09 versus 1.65 ± 0.30; Mann-Whitney U-test, * $p<0.05$). Please note, all graphs are scaled as in Figure 1 for direct comparison to mature males.

A

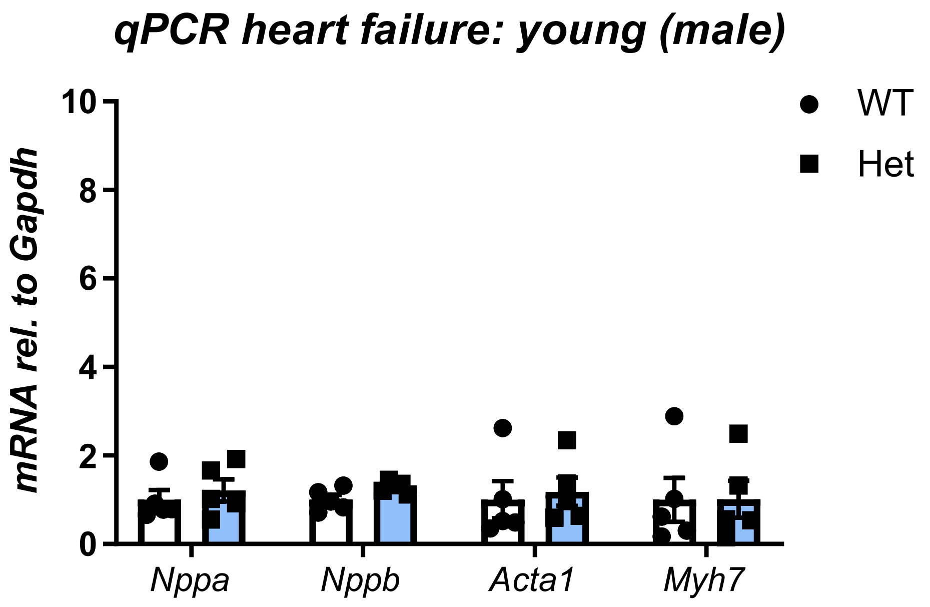
qPCR heart failure: young (male)
mRNA rel. to Gapdh
10
8
6
4
2
0
WT
Het
Nppa
Nppb
Acta1
Myh7

B

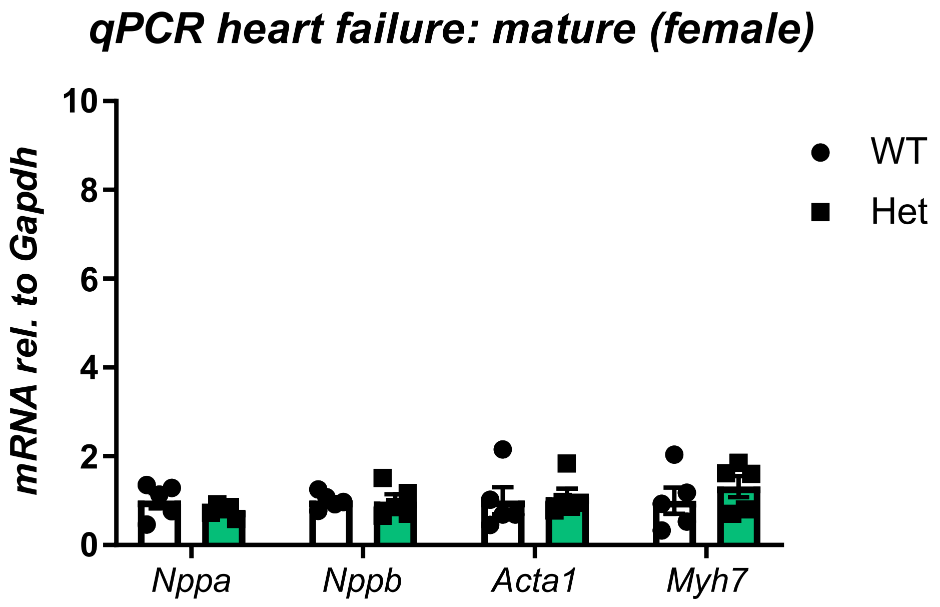
qPCR heart failure: mature (female)
mRNA rel. to Gapdh
10
8
6
4
2
0
WT
Het
Nppa
Nppb
Acta1
Myh7

C

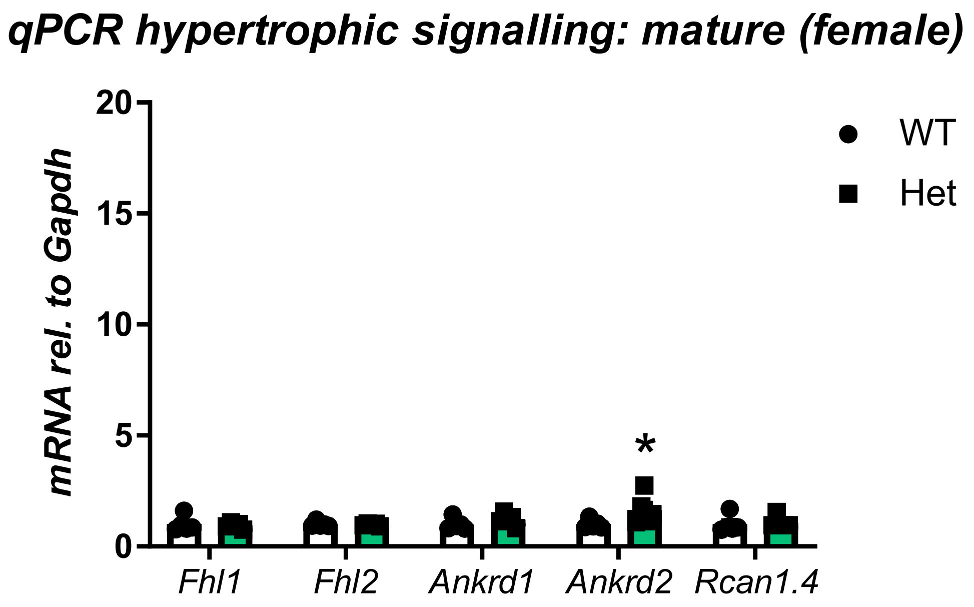
qPCR hypertrophic signalling: mature (female)
mRNA rel. to Gapdh
20
15
10
5
0
WT
Het
*
Fhl1
Fhl2
Ankrd1
Ankrd2
Rcan1.4

Figure S3. (A) Gross cardiac morphology shown on haematoxylin-eosin stained paraffin heart sections for WT and Het (aged 110 d, both males). Scale bar represents 2 mm. (B) In higher magnification, haematoxylin-eosin staining is normal. Scale bar represents 20 microns.

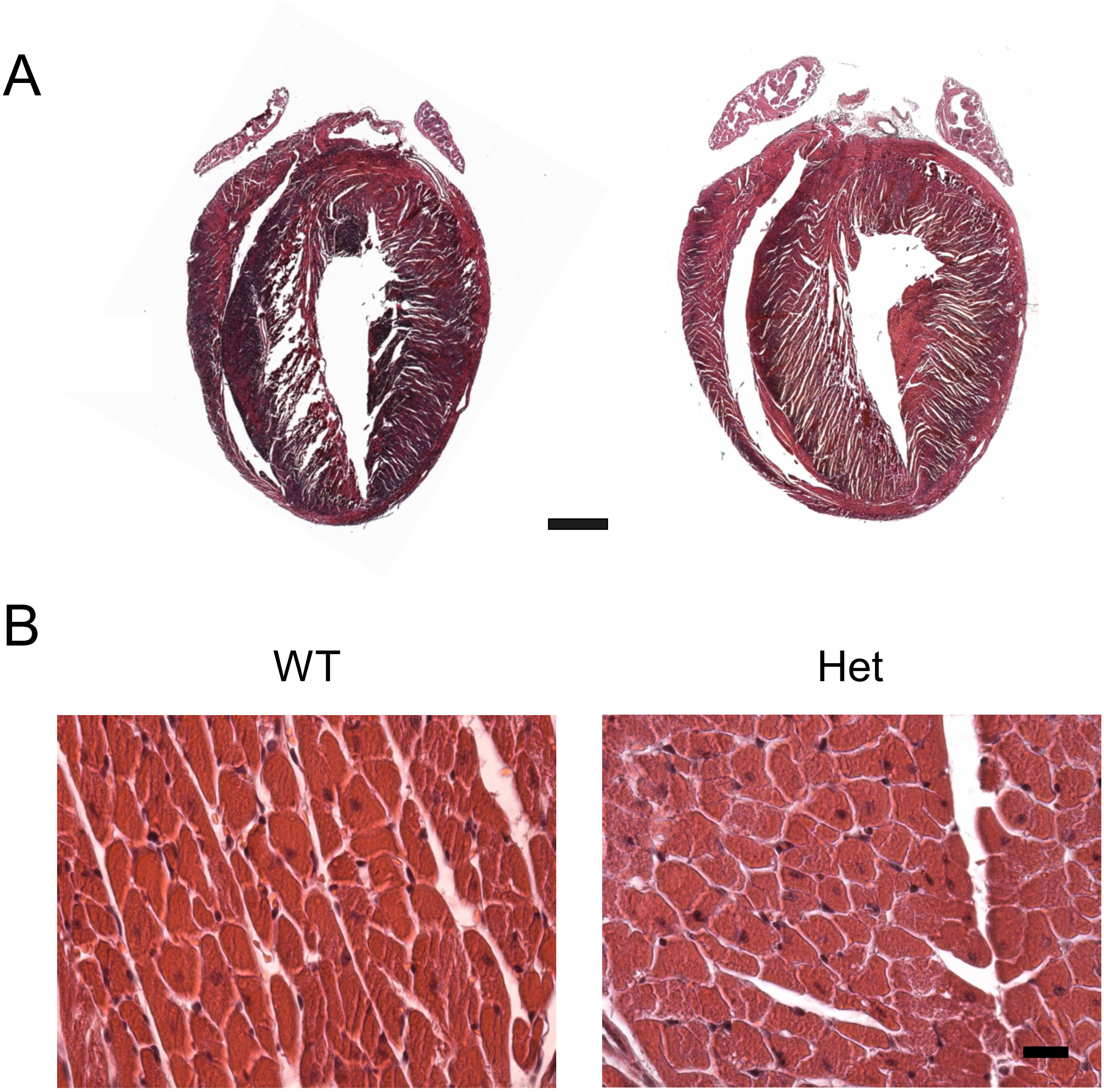
A
B
WT
Het

Figure S4. Analysis of proteins in mature male mice. (A) Western blots for beta-myosin heavy chain (Myh7), heat shock protein B7 (HspB7), heat shock protein 27 (Hsp27), alpha-actinin 2 (Actn2) and Gapdh (n = 3 per group, age 266 ± 1 d); position of marker bands indicated (molecular weight in kD). (B) Quantification indicates significant upregulation of HspB7 in Het mice (Student's *t*-test, ** $p<0.01$), and a visual trend not reaching significance for Myh7 ($p=0.099$). Please note, alpha-actinin 2 levels are normal in Het mice. (C) Western blot for ubiquitinated proteins (top) and Gapdh (bottom, loading control); position of marker bands indicated (molecular weight in kD). Arrow indicates theoretical position of ubiquitinated alpha-actinin, which could not be detected.

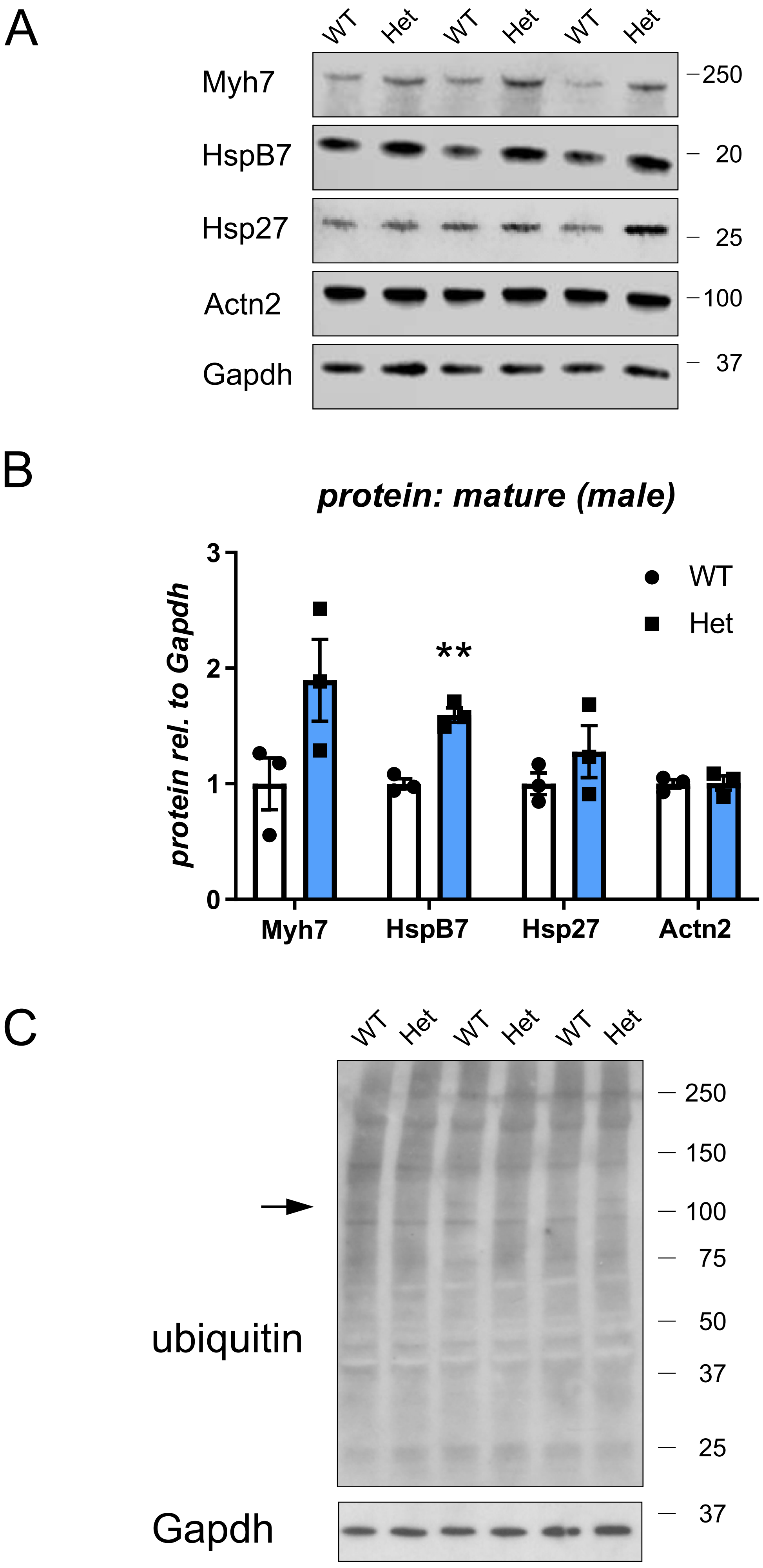
A
WT
Het
WT
Het
WT
Het
Myh7
HspB7
Hsp27
Actn2
Gapdh
250
20
25
100
37
B
protein: mature (male)
protein rel. to Gapdh
3
2
1
0
WT
Het
**
Myh7
HspB7
Hsp27
Actn2
C
WT
Het
WT
Het
WT
Het
250
150
100
75
50
37
25
ubiquitin
Gapdh
37

Figure S5. Normal skeletal muscle histology in aged male mice (n = 2 per group, age 266 ± 1 d). Cryosections of tibialis anterior were stained for myosin (red), laminin (green) and nuclei are visualised with DAPI (blue). Fibre size is normal in heterozygous *Actn2* p. Met228Thr skeletal muscle, and there is no evidence of centrally located nuclei, which would indicate muscle regeneration. Scale bar represent 50 microns.

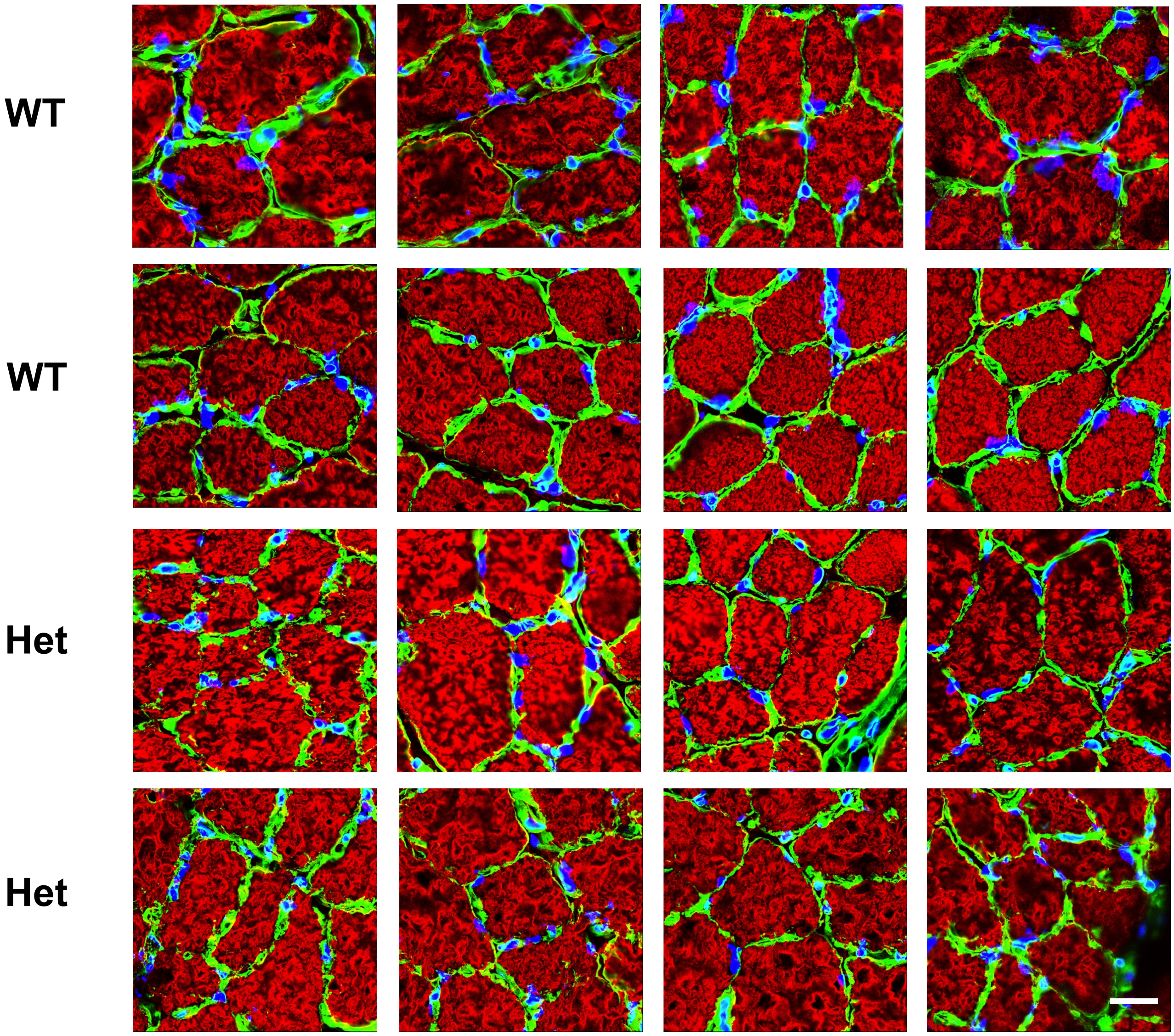
WT
WT
Het
Het

Figure S6. Pie charts of genotype distributions of (A) offspring at weaning, (B) at birth (P0) and at (C) embryonic stage E15.5. At weaning or at birth, no Hom mice (black) were found, while the genotype distribution is in agreement with expected Mendelian ratios at E15.5. (n indicates total number of animals genotyped at the time point).

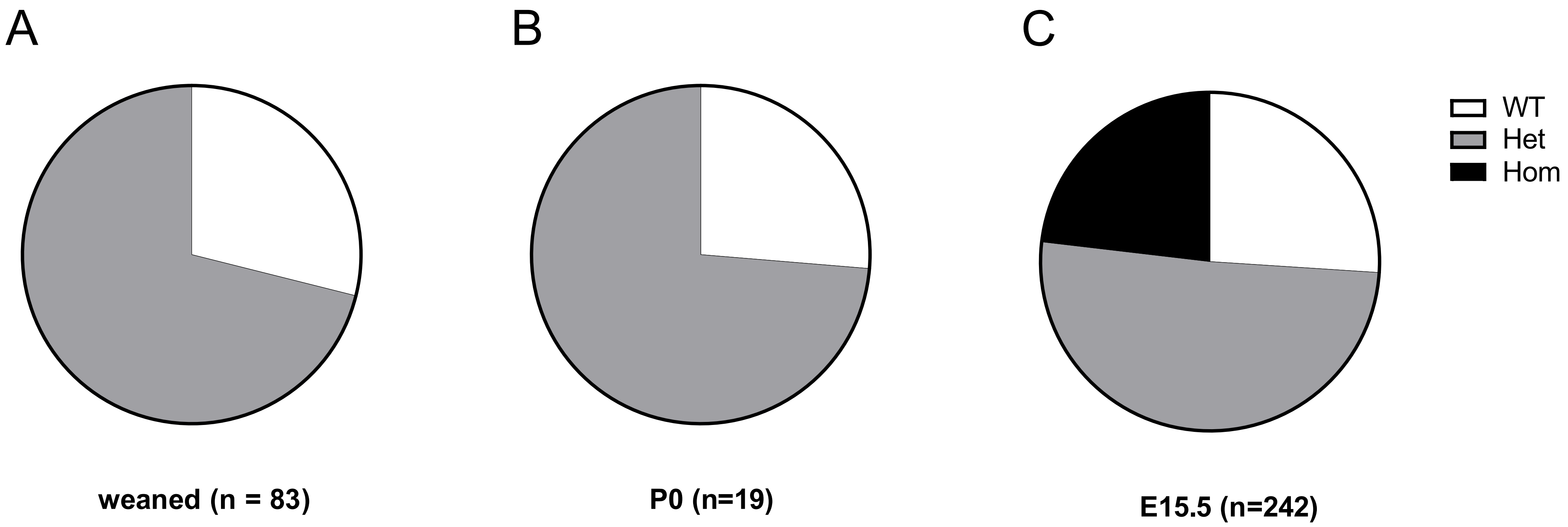
A
B
C
WT
Het
Hom
weaned (n = 83)
P0 (n=19)
E15.5 (n=242)

Figure S7. Appearance and body weights of embryos at embryonic stage E15.5. (A) Photos of two litters at E15.5 with genotypes for each embryo indicated. Please note the slightly different magnification in both images. Scale bars represent 1 cm. (B) No differences in body weight were observed between the three genotypes at E15.5 (one-way ANOVA).

A

E15.5 embryos

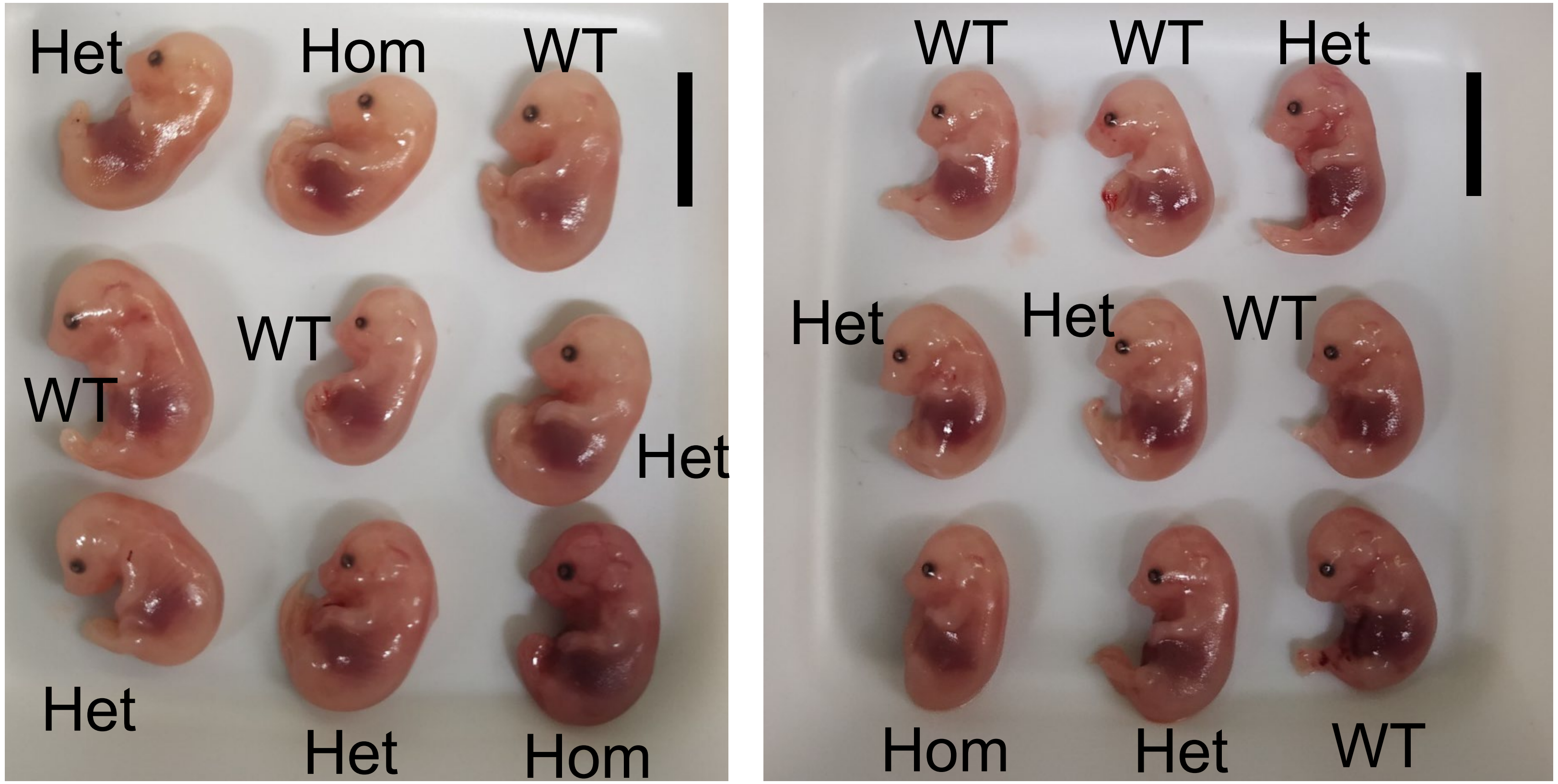

B

Body weight

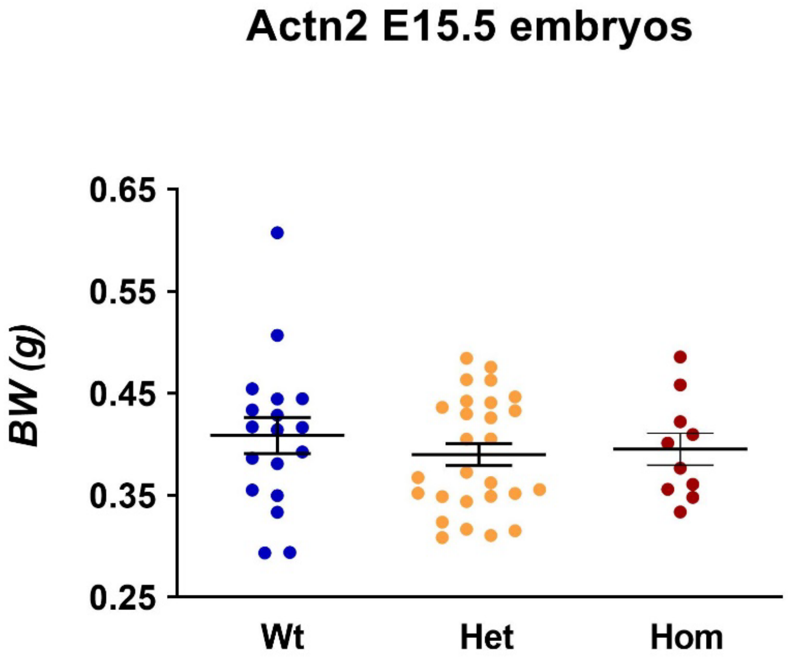

Figure S8. 3D reconstructions of embryonic heart compartments. WT and Hom E15.5 hearts were investigated for morphology and volume of the left (green) and right atria (yellow), left (blue) and right ventricular lumens (red). Ventricles including the ventricular wall is coloured in grey. For volumetric data, see Figure. 2. Please note, these images are not scaled.

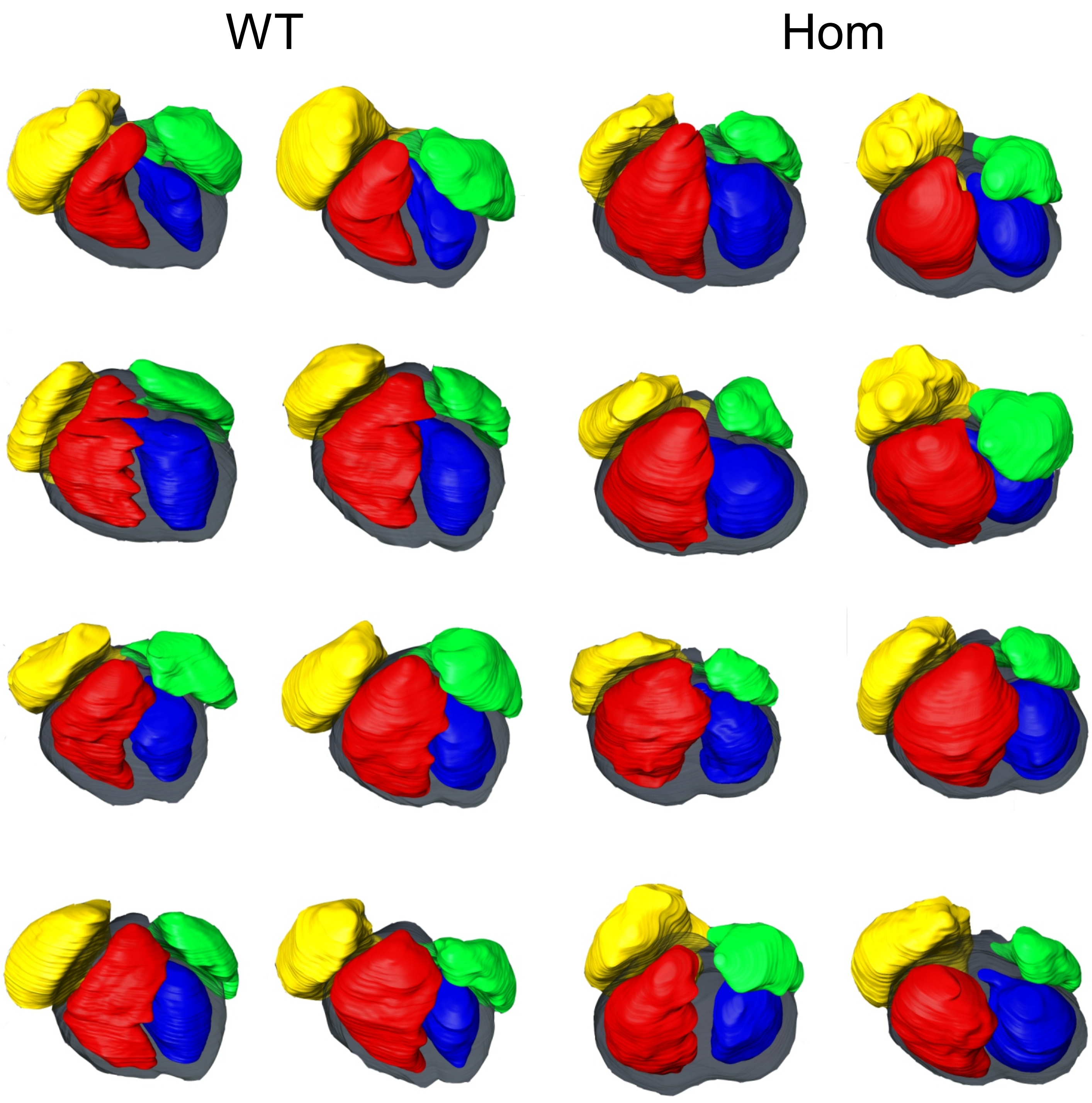
WT
Hom

Figure S9. Homozygous embryos have reduced myocardial wall thickness in the left ventricle. (A) Diagrammatic representation of sampling points for wall thickness of the E15.5 heart. Lengths 2 and 3 were taken close to the ventricular septum while lengths 1 and 4 were taken at a 45-degree angle to the septum. (B) Myocardial wall thickness measured in two segments per ventricle. Data was normally distributed, so Student's *t*-tests were used for each length. n = 8 WT (white bars), n = 8 Hom (grey bars), ** $p<0.01$.

A

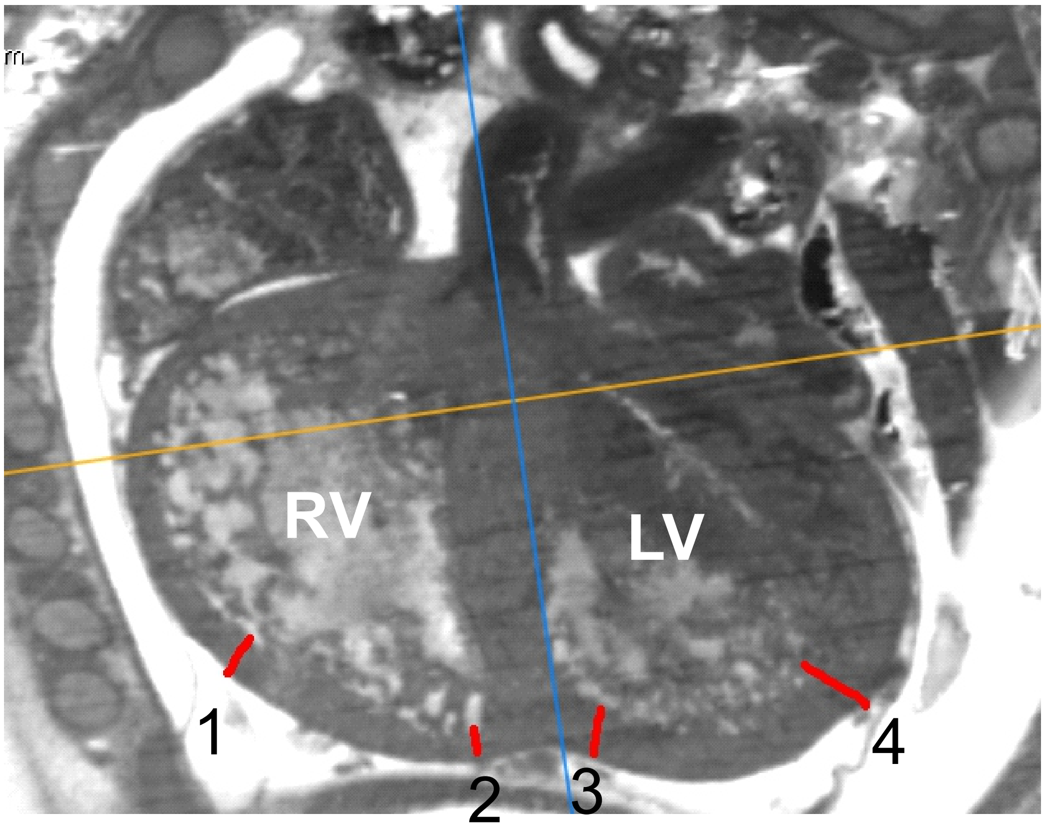
RV
LV
1
2
3
4

B

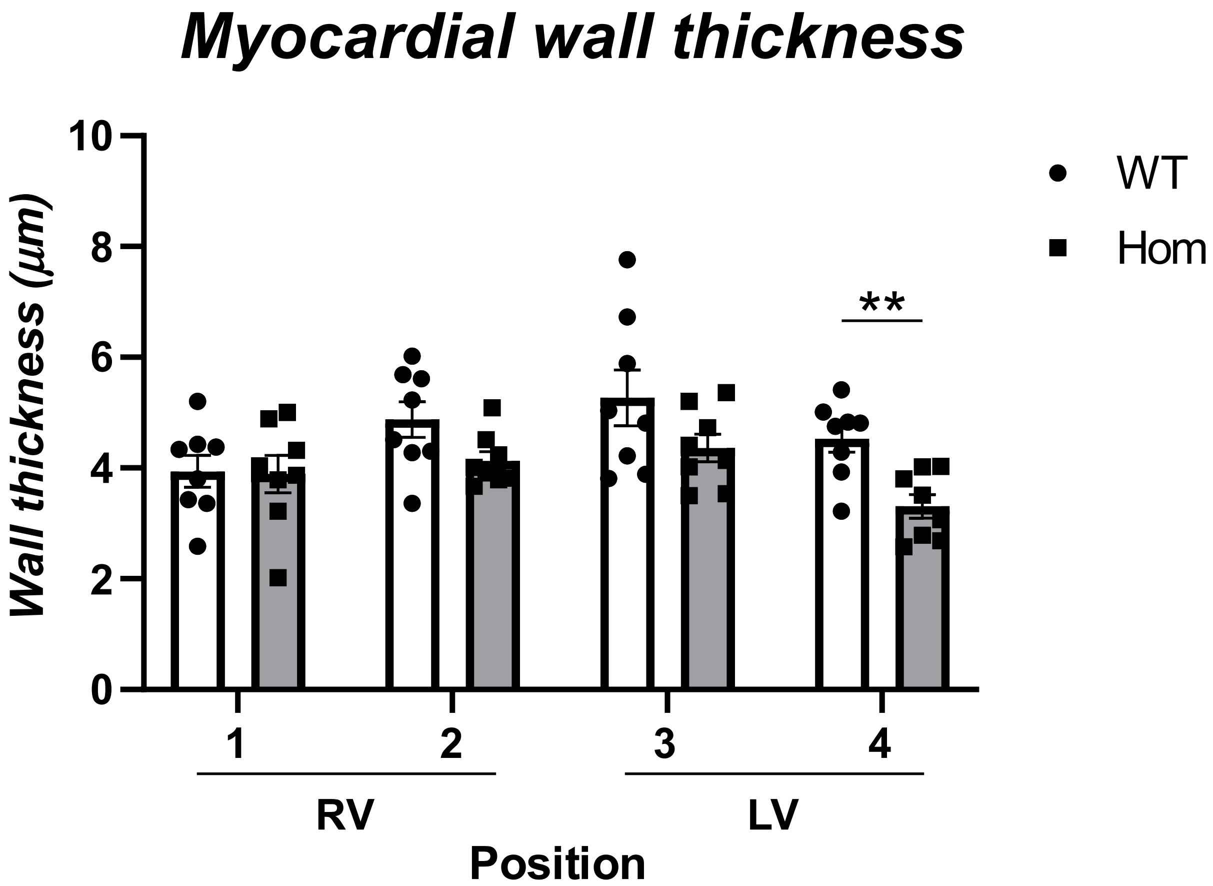
Myocardial wall thickness
10
8
6
4
2
0
Wall thickness (μm)
WT
Hom
**
1
2
3
4
RV
LV
Position

Figure S10. Homozygous embryonic hearts have altered aortic arch and pulmonary trunk structure. (A) 3D model of aortic arch (cerulean) and pulmonary trunk (PT, magenta) for a Hom heart. (B) Cross-sectional length of segments for the aorta (Ao, 1,4,5) and PT (2,3,6) were measured. (dAo- descending aorta, AoV- aortic valve) (C) Relative (percentage) PT volume to control. For B, data was normally distributed in segment 1, so a Student's *t*-test was used for this length (** $p<0.01$). The remaining segments 2-6 lengths for B and data in C used non-parametric Mann-Whitney U test (* $p<0.05$, ** $p<0.01$). n = 8 WT (white bars), n = 8 Hom (grey bars).

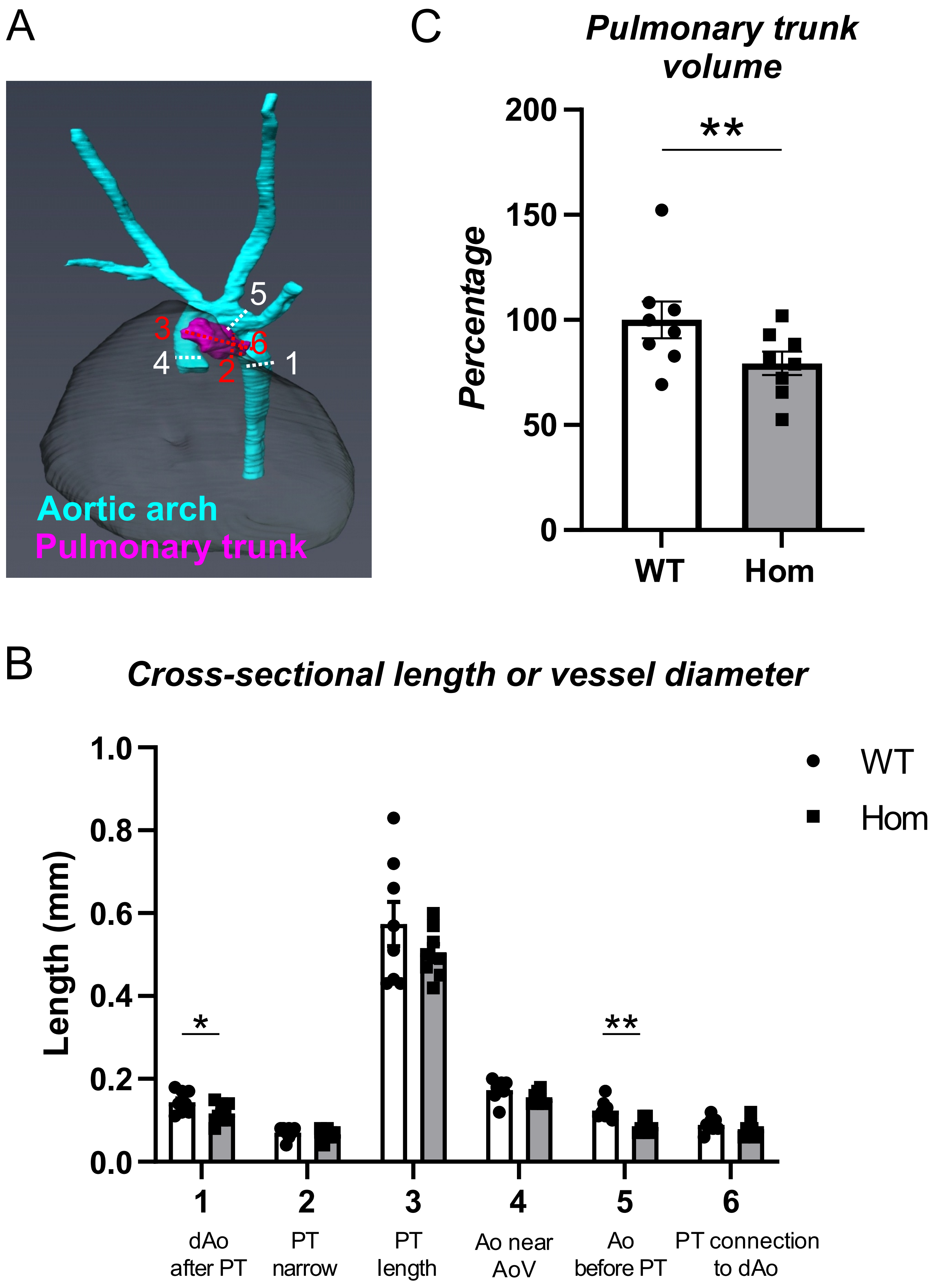
A
5
3
6
4
2
1
Aortic arch
Pulmonary trunk
C
Pulmonary trunk volume
Percentage
200
150
100
50
0
**
WT
Hom
B
Cross-sectional length or vessel diameter
Length (mm)
1.0
0.8
0.6
0.4
0.2
0.0
*
**
WT
Hom
1
dAo after PT
2
PT narrow
3
PT length
4
Ao near AoV
5
Ao before PT
6
PT connection to dAo

Figure S11. Homozygous embryonic hearts have pulmonary valve dysplasia. (A) Diagrammatic representation and location of semilunar valve leaflets in the context of other heart structures. (B) E15.5 3D models comparing WT and Hom semilunar valves. Superior pulmonary valve and inferior aortic valve leaflets are shown. (C) Relative (percentage) volume of semilunar valve leaflets in Hom hearts compared to WT. Pulmonary valve (PA) non-facing intercalated leaflet (nf, yellow), pulmonary left (L, red), and pulmonary right (R, blue); and aortic (Ao) non-coronary intercalated leaflet (nc, pink), aortic left (L, brown), and aortic right (R, green) are indicated. Non-parametric Mann-Whitney U tests were used for aortic non-facing intercalated leaflet, and pulmonary left leaflet. The remainder used Student's *t*-tests. n = 8 WT (white bars), n = 8 Hom (grey bars), ** $p<0.01$, *** $p<0.001$.

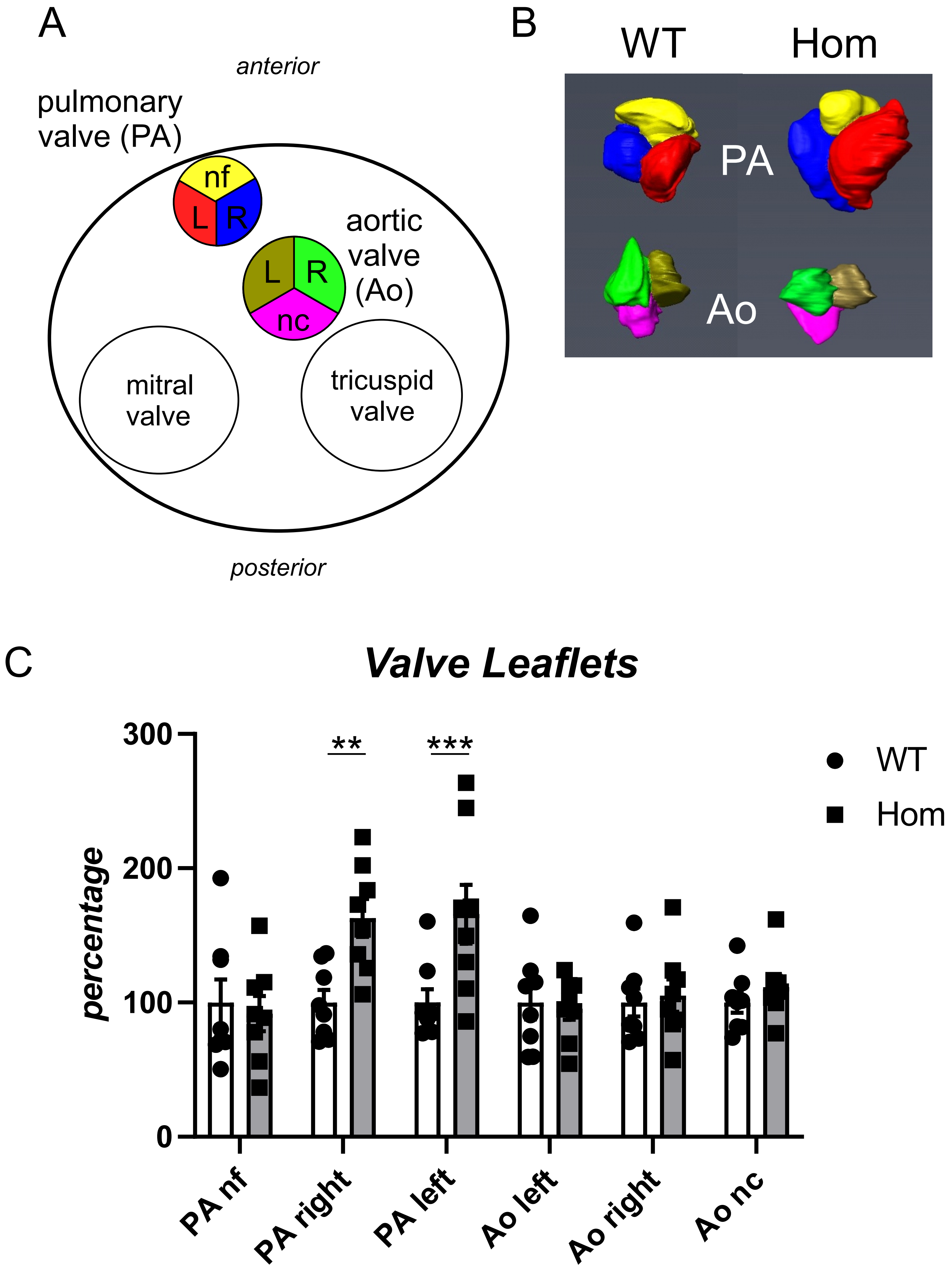
A
anterior
pulmonary valve (PA)
nf
L
R
aortic valve (Ao)
L
R
nc
mitral valve
tricuspid valve
posterior
B
WT
Hom
PA
Ao
C
Valve Leaflets
300
200
100
0
percentage
**
***
WT
Hom
PA nf
PA right
PA left
Ao left
Ao right
Ao nc

Figure S12. Homozygous embryonic hearts are mal-positioned within the body cavity. (A) 3D reconstructions of the E15.5 embryo, heart and aortic arch in the context of the frontal, lateral and cranial aspects, further investigated in B and C. (B) Comparative representations of 8 WT and 8 Hom embryonic hearts in frontal, lateral and cranial views. White lines delineate measured angles of vertical or lateral rotation. (C) Lateral rotation (measured from base of the aorta (Ao) to ventricular sulcus) and vertical rotation (measured from posterior to the distal tip of the heart). Data was not normally distributed, so was analysed by Mann-Whitney U test, * $p<0.05$. n = 8 WT (white bars), n = 8 Hom (grey bars).

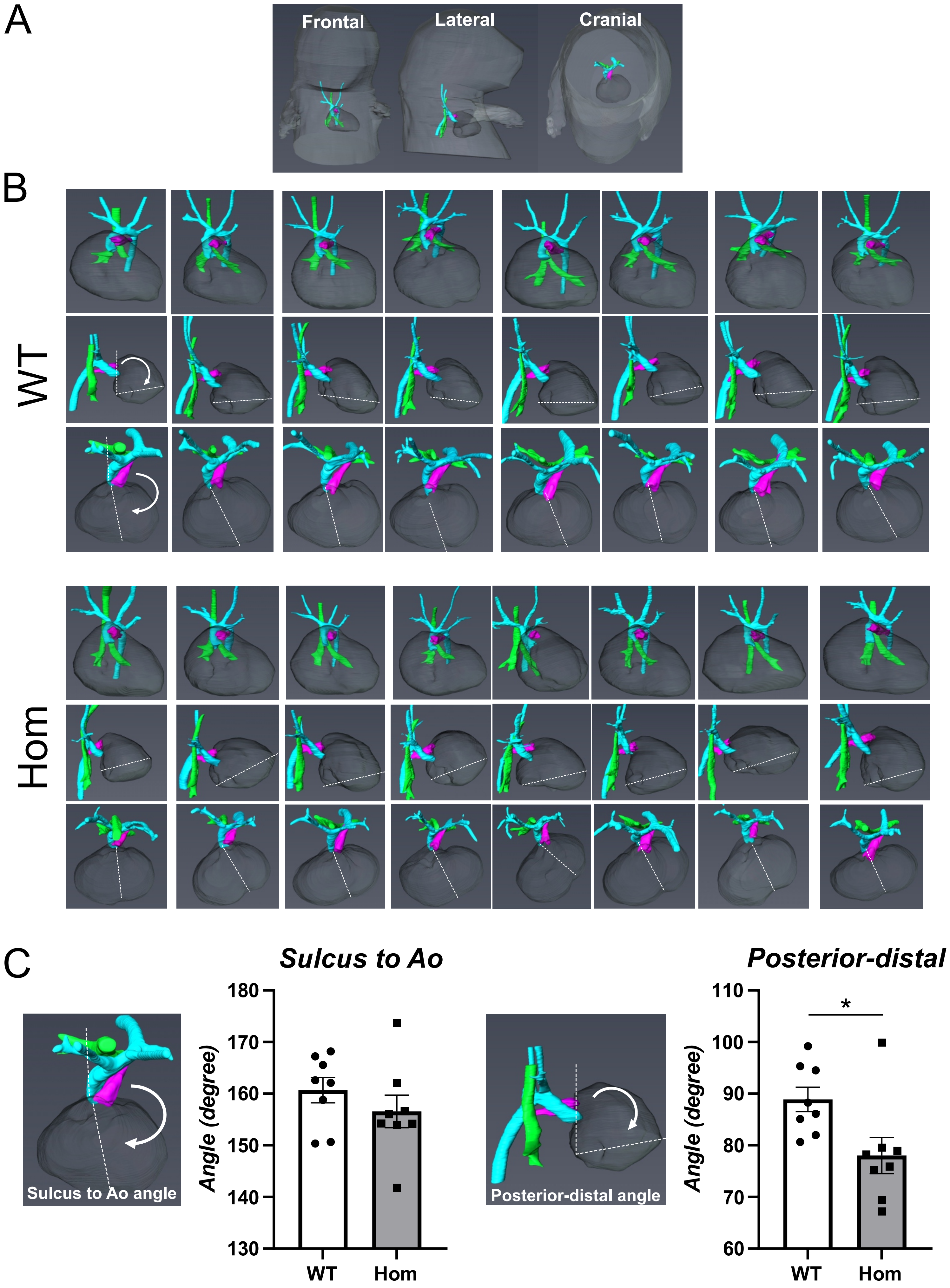
A
Frontal
Lateral
Cranial
B
WT
Hom
C
Sulcus to Ao
180
170
160
150
140
130
Angle (degree)
WT
Hom
Sulcus to Ao angle
Posterior-distal
110
100
90
80
70
60
*
Posterior-distal angle

Figure S13. Theiler staging of embryos at embryonic stage E15.5. For each genotype, the percentage of embryos classed as Theiler stage 22+ (white), 23- (grey) and 23 (black) is given. Total number of embryos: WT n = 26, Het n = 58, Hom n = 12. No significant differences were observed between genotypes (Chi-square p=0.20).

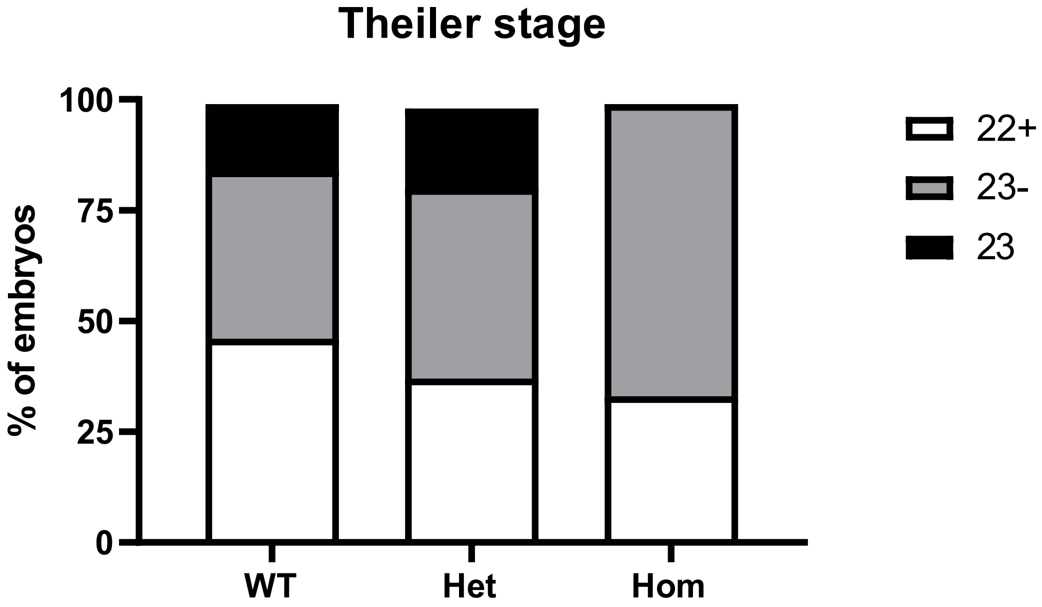
Theiler stage
% of embryos
100
75
50
25
0
WT
Het
Hom
22+
23-
23

Figure S14. Embryonic hearts at E15.5 after wholemount staining. Hom have dark areas indicating congealed blood in the cavities (arrows), which is not found to the same extent in WT hearts. This might indicate poor residual contractility during the fixation process. Scale bar represents 2 mm.

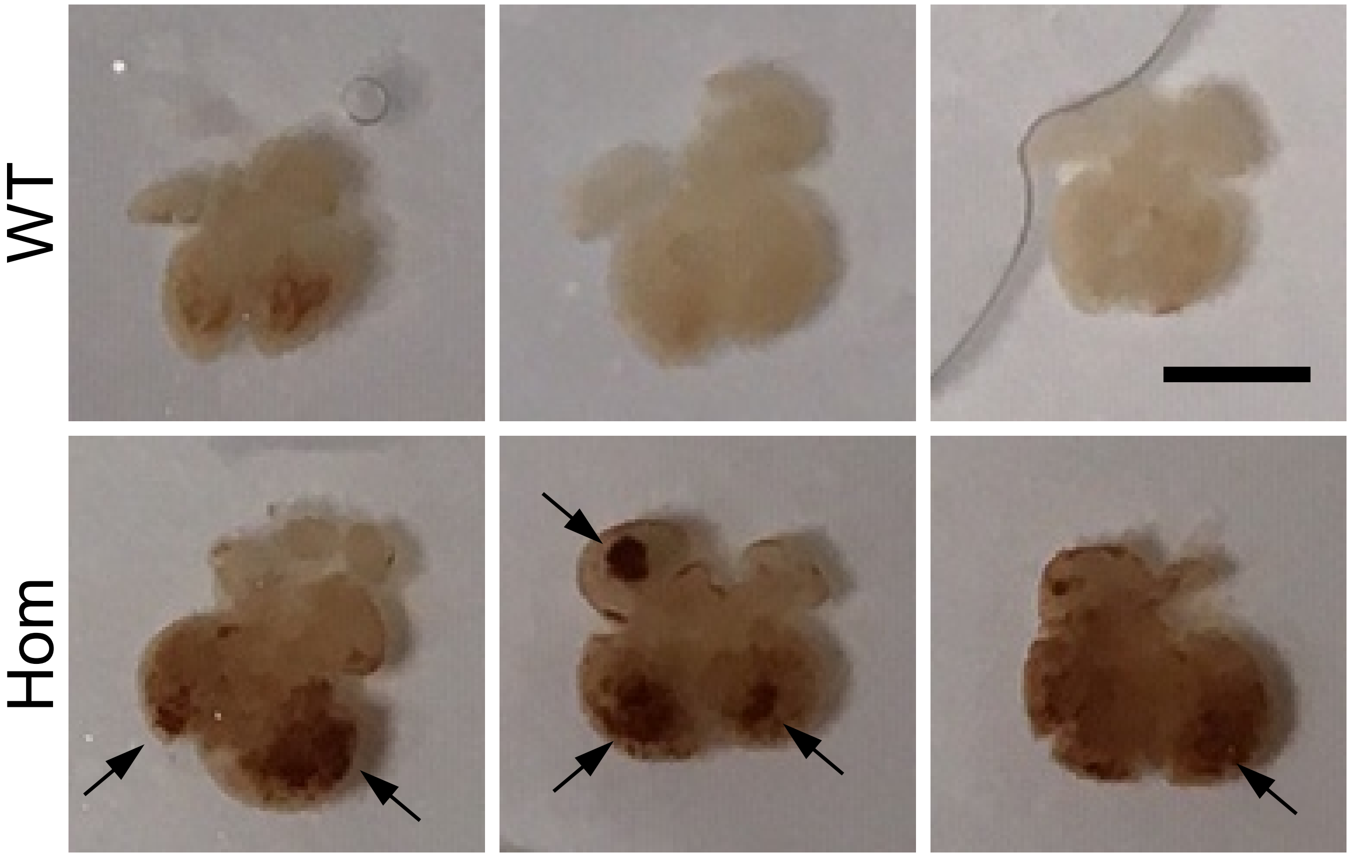
WT
Hom

Figure S15. Ultrastructural analysis of sarcomeres in E15.5 hearts. Sarcomere structures of (A) WT and (B) Hom hearts were analysed by transmission electron microscopy. While sarcomeres were present and regular in both WT and Hom hearts. (C) Higher magnification indicated less uniform Z-disk appearance in Hom hearts. This is reflected in a wider Z-disk distribution in Hom hearts. The average Z-disk width was not significantly changed. (D) Data was not normally distributed, so was analysed by the non-parametric Mann-Whitney U test. n = 26 WT (left), n = 77 Hom (right) from three independently processed hearts per genotype. Scale bars represent 1 micron.

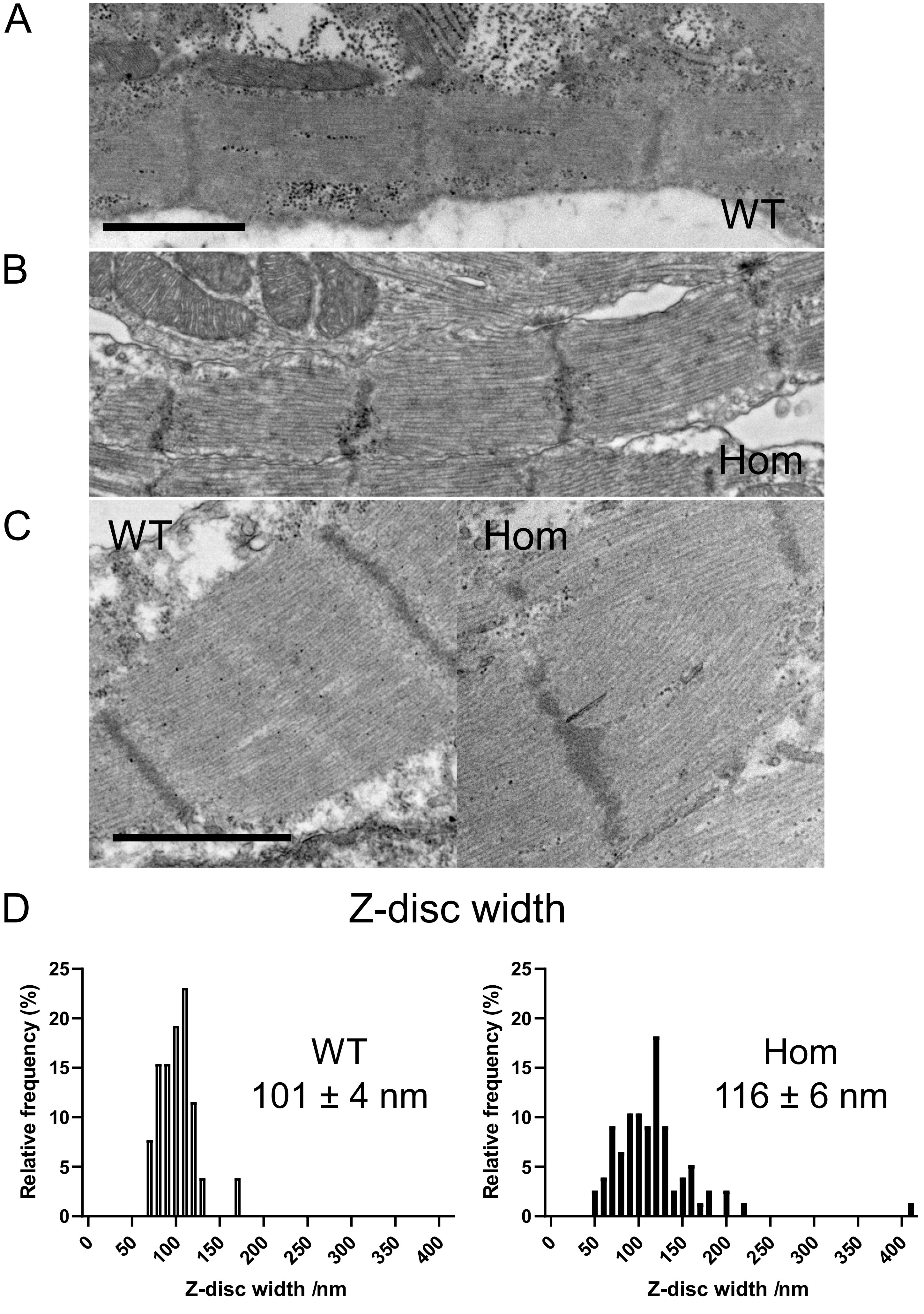
A
WT
B
Hom
C
WT
Hom
D
Z-disc width
Relative frequency (%)
WT
101 ± 4 nm
Z-disc width /nm
Relative frequency (%)
Hom
116 ± 6 nm
Z-disc width /nm

Figure S16. Trend towards increased p53 protein levels in Hom E15.5 hearts. (A) Western blot for p53 (n = 5 WT/ n = 6 Hom), PonceauS stain of the membrane (cardiac actin band) is used as a loading control; position of marker bands are indicated (molecular weight in kDa). (B) Quantification indicates a trend of increased p53 protein levels in Hom E15.5 hearts, but it does not reach significance (p = 0.13 Student's *t*-test).

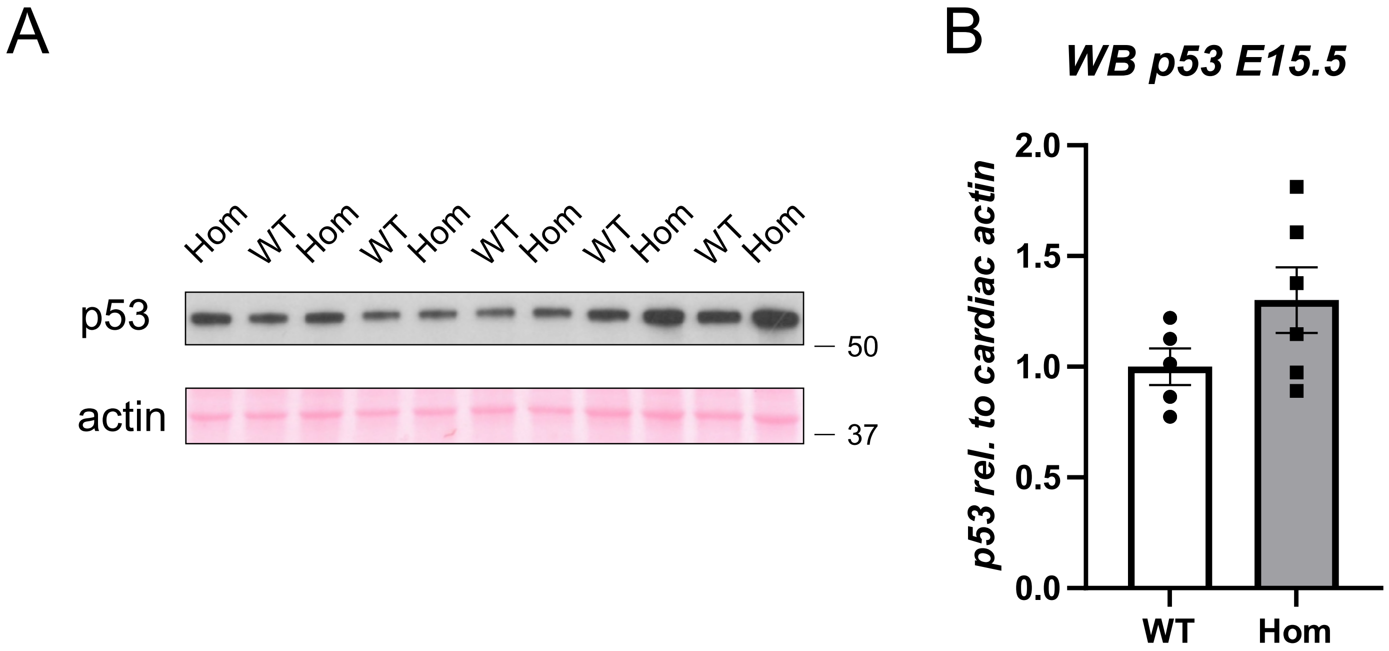
A
Hom
WT
Hom
WT
Hom
WT
Hom
WT
Hom
WT
Hom
p53
actin
50
37
B
WB p53 E15.5
p53 rel. to cardiac actin
2.0
1.5
1.0
0.5
0.0
WT
Hom

Figure S17. Myofibrillar disassembly defects in dividing E15.5 Hom cells. Ventricular whole mount stainings were labelled for phospho-Histone H3 (pH3, Upstate) and alpha-actinin 2, nuclei were stained with DAPI. Merge images show pH3 in green, alpha-actinin 2 in red and DAPI in blue. Scale bar represents 10 microns. In dividing, pH3-positive Hom cells with metaphase chromosome arrangement residual sarcomeric structures (arrowheads) are found, while WT cells have completely disassembled their sarcomeres at this stage. This suggests a defect in myofibril disassembly in Hom E15.5 dividing cells.

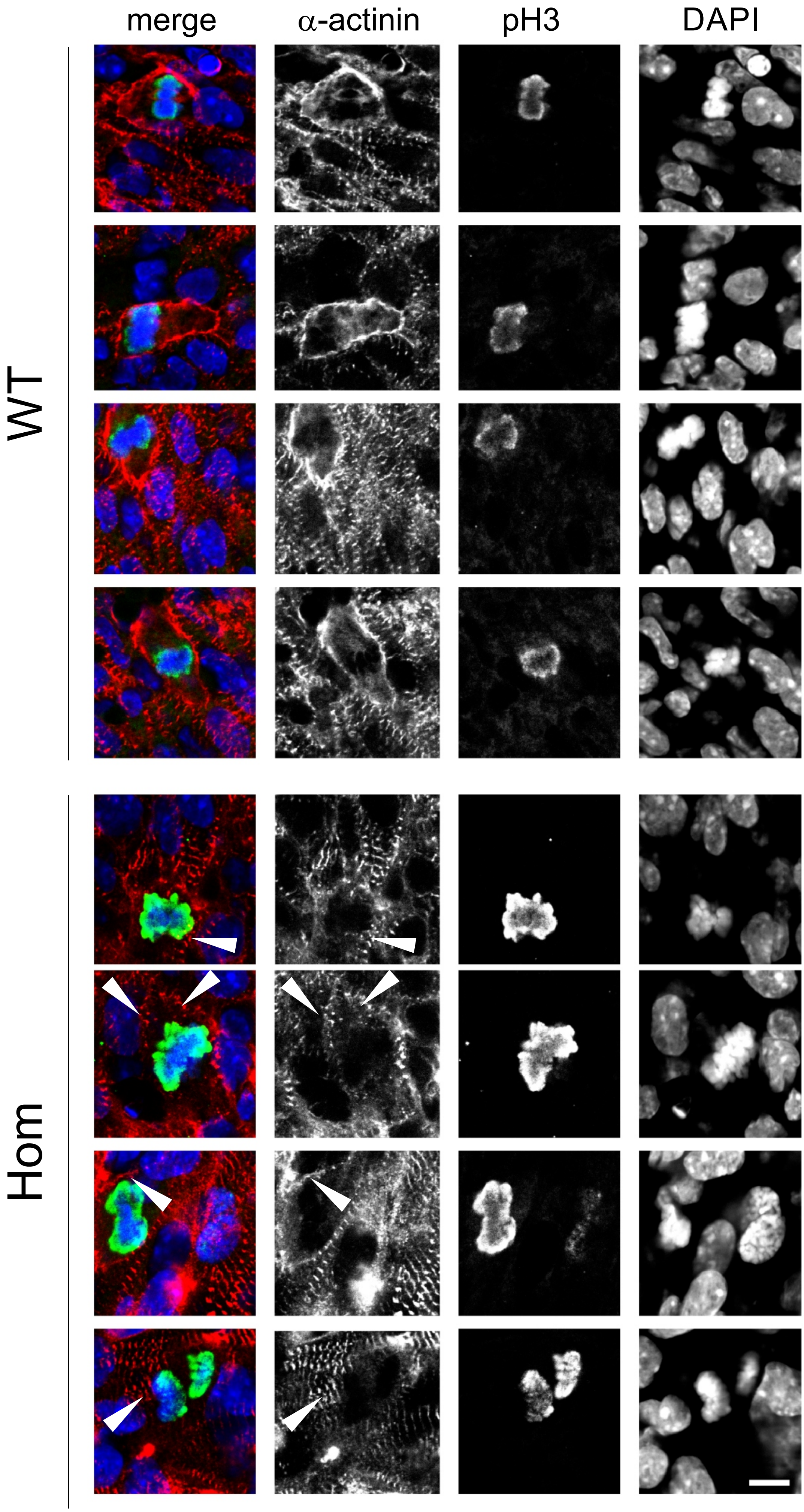
merge
α-actinin
pH3
DAPI
WT
Hom

Figure S18. Mitochondrial dysfunction and oxidative phosphorylation were identified as the top dysregulated pathways by IPA analysis (Figure. 5B). Here all the proteins associated with the pathway are overlaid with the results from MS analysis. Green represents decreased measurement while red represents increased measurement. Orange represents predicted activation while blue represents predicted inhibition based on the IPA database. Mitochondrial complex I, II (succinate dehydrogenase), III (Cytochrome bc1), IV (Cytochrome-c Oxidase) and V (ATP Synthase) are all impacted in Hom embryo hearts at E15.5.

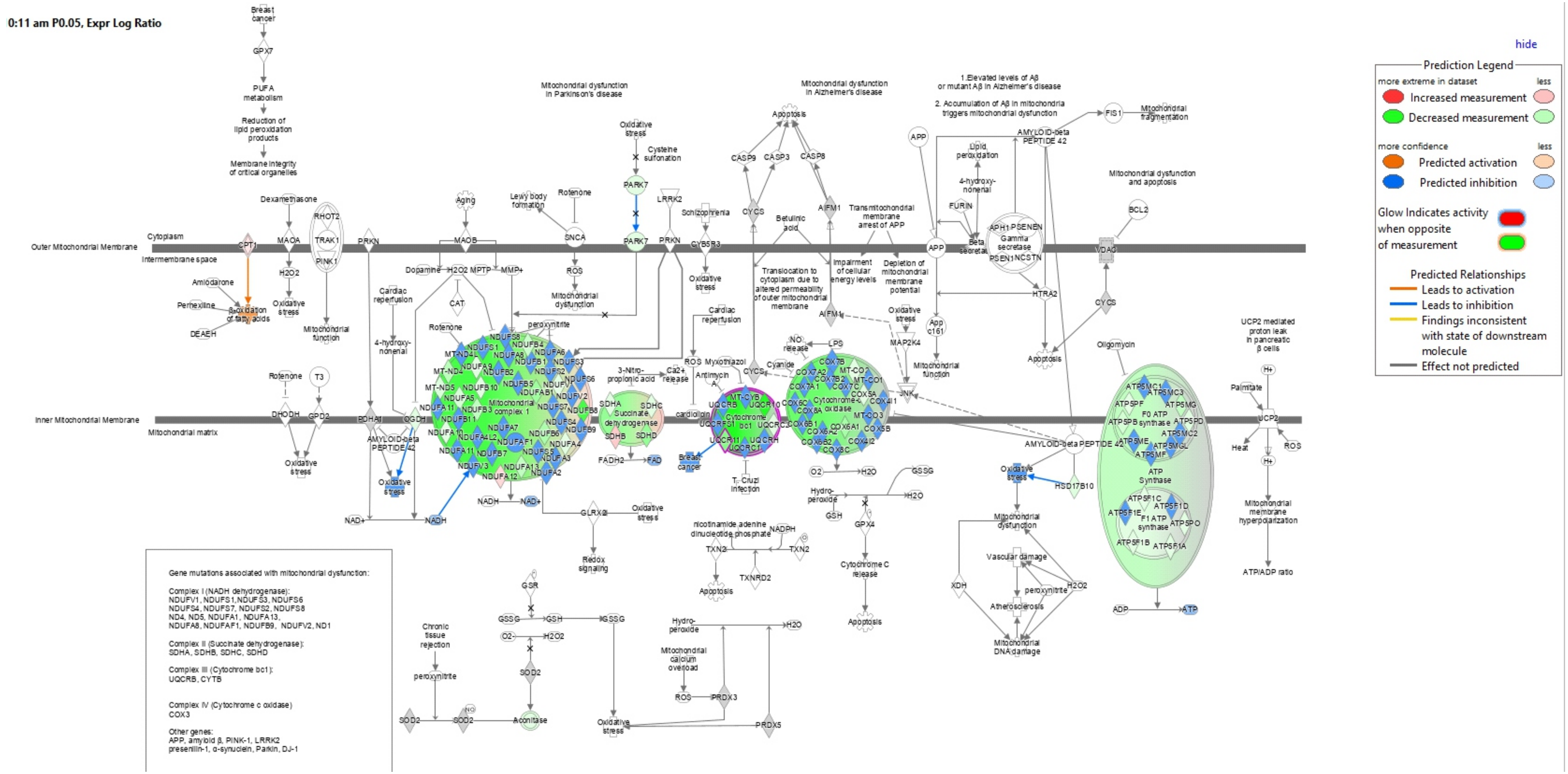
0:11 am P0.05, Expr Log Ratio
hide
Prediction Legend
more extreme in dataset
less
Increased measurement
Decreased measurement
more confidence
less
Predicted activation
Predicted inhibition
Glow indicates activity when opposite of measurement
Predicted Relationships
Leads to activation
Leads to inhibition
Findings inconsistent with state of downstream molecule
Effect not predicted
Cytoplasm
Outer Mitochondrial Membrane
Intermembrane space
Inner Mitochondrial Membrane
Mitochondrial matrix
Gene mutations associated with mitochondrial dysfunction:
Complex I (NADH dehydrogenase):
NDUFV1, NDUFS1, NDUFS3, NDUFS6
NDUFS4, NDUFS7, NDUFS2, NDUFS8
ND4, ND5, NDUFA1, NDUFA13,
NDUFA8, NDUFAF1, NDUFB9, NDUFV2, ND1
Complex II (Succinate dehydrogenase):
SDHA, SDHB, SDHC, SDHD
Complex III (Cytochrome bc1):
UQCRB, CYTB
Complex IV (Cytochrome c oxidase)
COX3
Other genes:
APP, amyloid β, PINK-1, LRRK2
presenilin-1, α-synuclein, Parkin, DJ-1

Figure S19. Alpha-actinin 2, but not 3 is upregulated in Hom E15.5 hearts. (A) Targeted assessment of transcriptional changes in *Actn2* by qPCR in WT and Hom E15.5. All measurements are normalised to Gapdh. Significant up-regulation of *Actn2* with three different probes (position indicated by which exons are spanned) are observed in the hearts of Hom mice (Student's *t*-test, ** p<0.01, *** p<0.001, **** p<0.0001, n = 6 per group). Please note data for probe spanning exons 11-12 are already shown in Fig. 6A. (B) Western blot for alpha-actinin 3 (Actn3) and Gapdh (n = 4 WT, n = 3 Hom); position of marker bands indicated (molecular weight in kD). Samples were run on the same blot, but not in adjacent positions. (C) Quantification indicates normal Actn3 protein levels in Hom E15.5 hearts (Student's *t*-test).

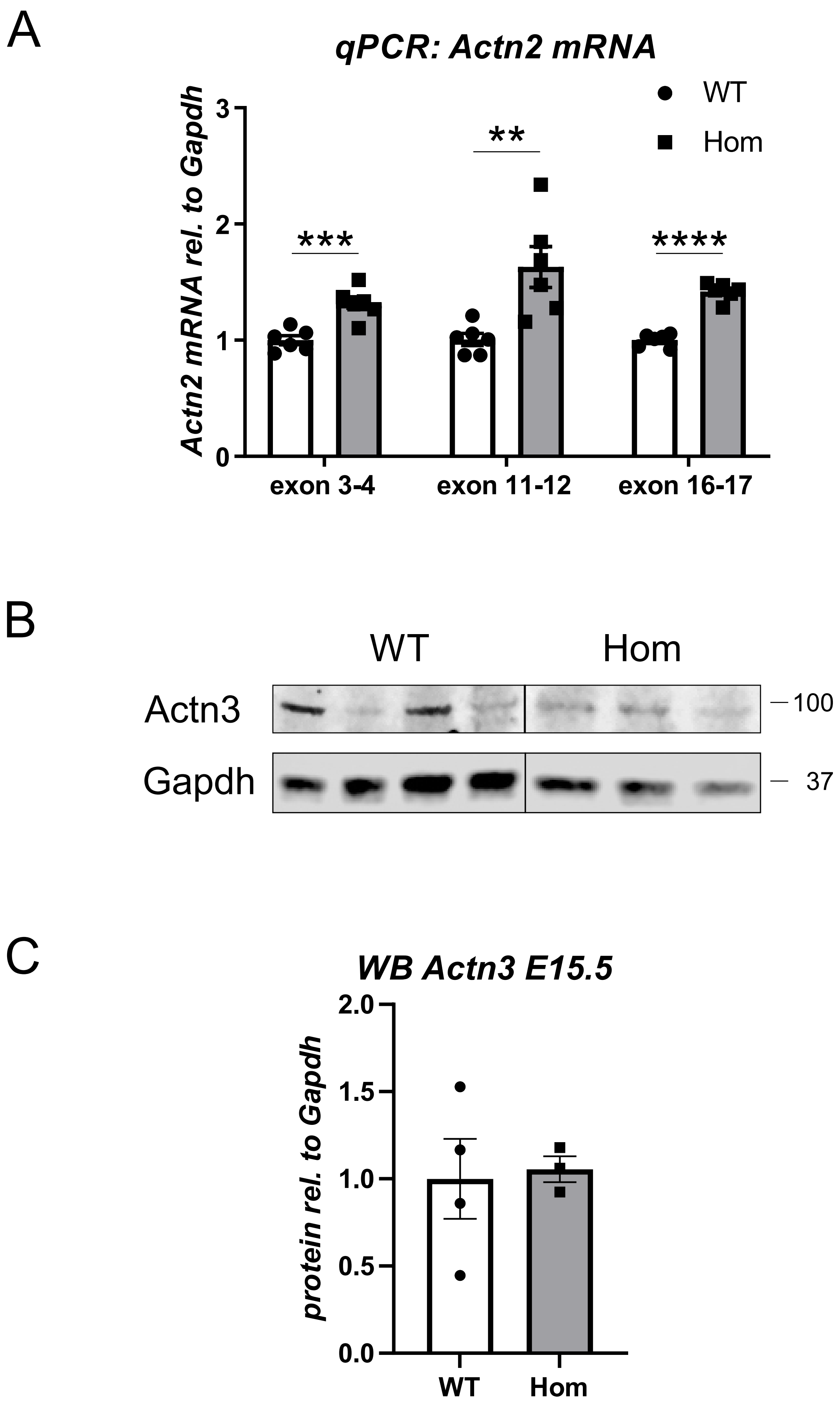
A
qPCR: Actn2 mRNA
Actn2 mRNA rel. to Gapdh
WT
Hom
0
1
2
3
***
**
****
exon 3-4
exon 11-12
exon 16-17
B
WT
Hom
Actn3
Gapdh
100
37
C
WB Actn3 E15.5
protein rel. to Gapdh
0.0
0.5
1.0
1.5
2.0
WT
Hom

Figure S20. No indication of increased proteasomal activity in Hom 15.5 hearts. (A) Measurement of proteasomal activities in embryonic hearts. Chymotrypsin-, trypsin- and caspase-like proteolytic activities were measured in tissue lysates from WT and Hom E15.5 hearts (n=5). Values are normalised to an average of 1 in the WT group. No changes were observed (Student's *t*-test). (B) Western blot for ubiquitinated proteins (top) and PonceauS (bottom, to visualise protein loading); position of marker bands indicated (molecular weight in kD). Arrow indicates theoretical position of ubiquitinated alpha-actinin, which could not be detected.

A

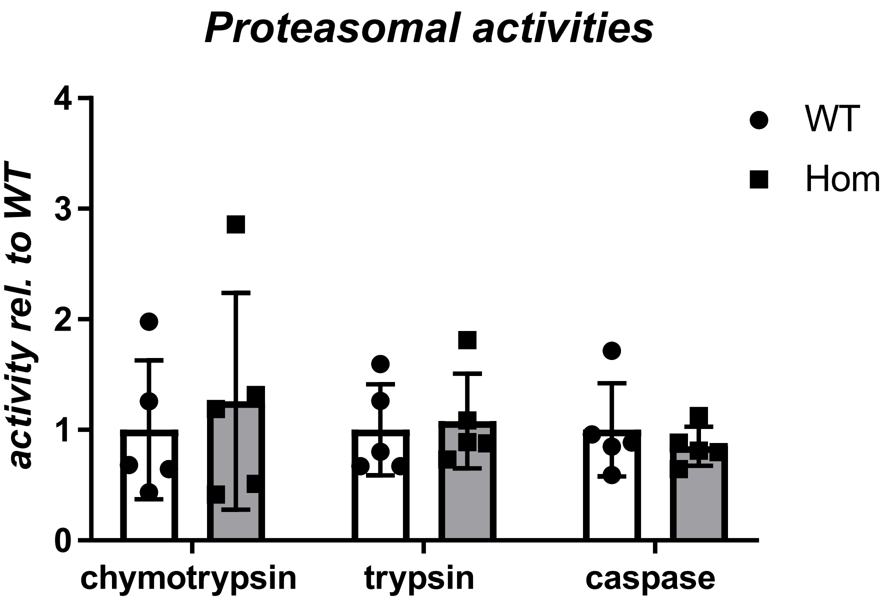
Proteasomal activities
activity rel. to WT
0
1
2
3
4
WT
Hom
chymotrypsin
trypsin
caspase

B

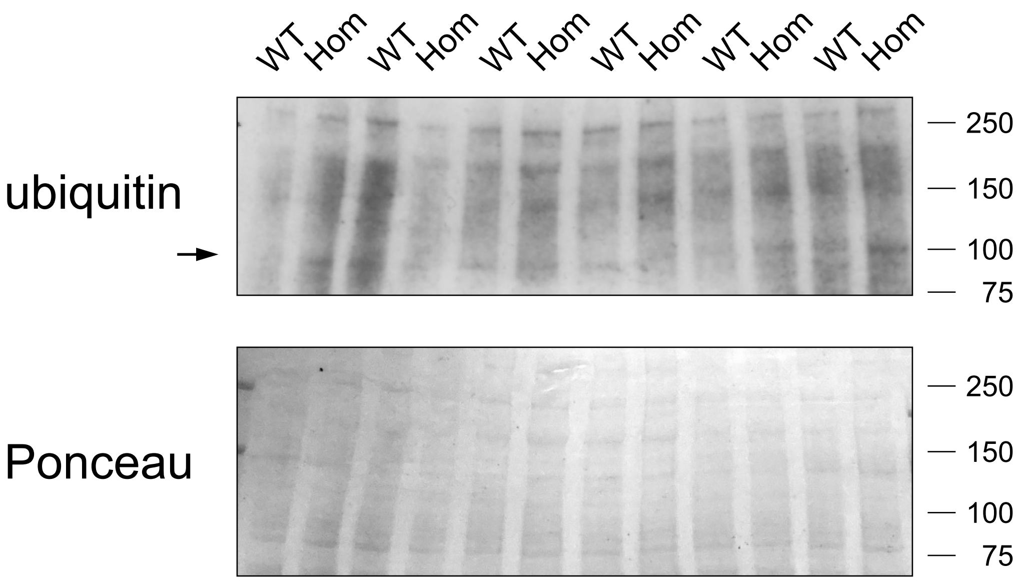
WT
Hom
WT
Hom
WT
Hom
WT
Hom
WT
Hom
WT
Hom
ubiquitin
Ponceau
250
150
100
75
250
150
100
75

Figure S21. No changes in markers of autophagy p62 and LC3 in Hom E15.5 hearts. (A) Western blots for p62, LC3 and Gapdh (n = 6 per group); position of marker bands indicated (molecular weight in kD). Gapdh is the same blot as already shown in Fig. 7B, as probed on the same membrane. (B) Quantification indicates normal levels of p62 and LC3 in Hom E15.5 hearts (Student's *t*-test).

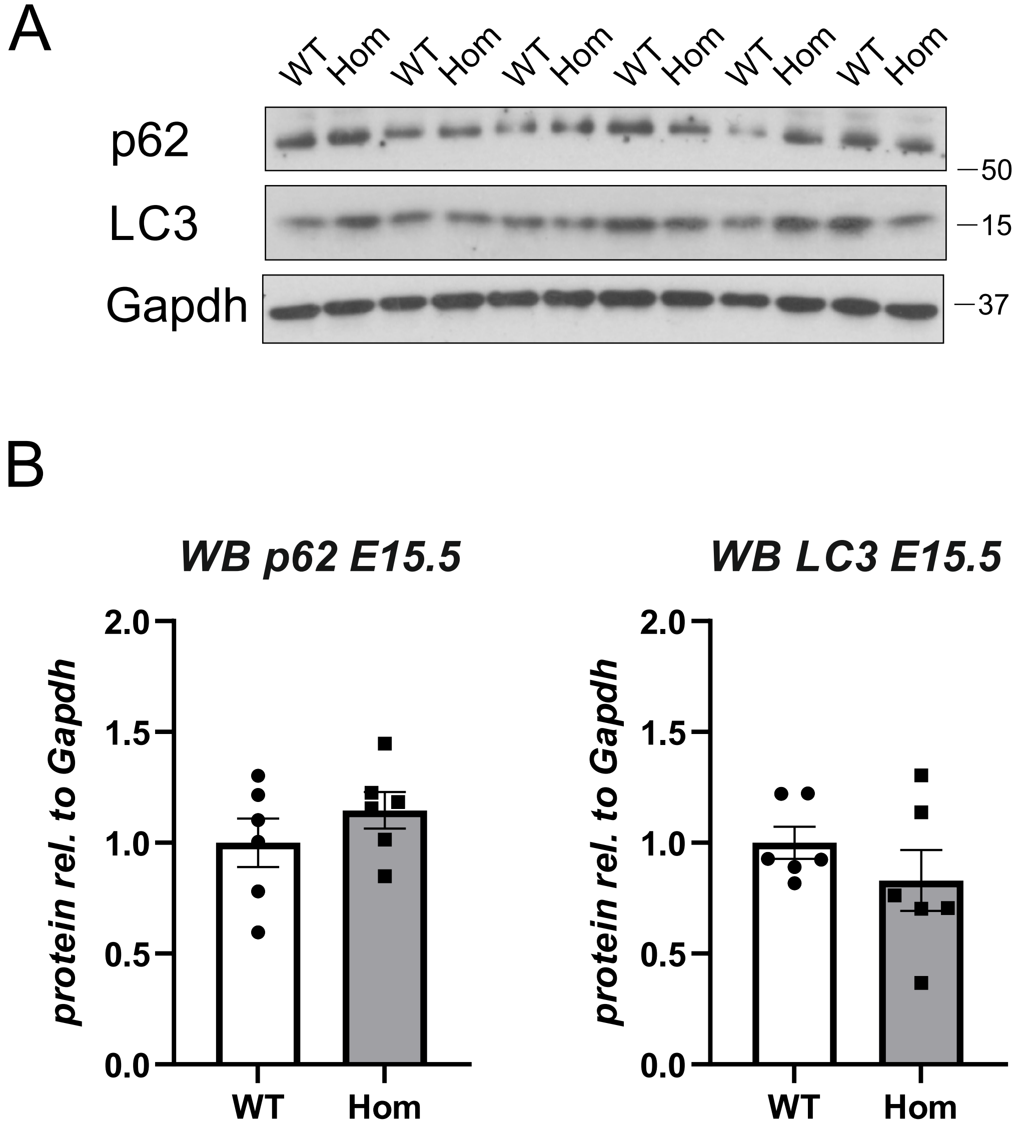
A
WT
Hom
WT
Hom
WT
Hom
WT
Hom
WT
Hom
WT
Hom
p62
LC3
Gapdh
50
15
37
B
WB p62 E15.5
WB LC3 E15.5
protein rel. to Gapdh
2.0
1.5
1.0
0.5
0.0
WT
Hom

Table S1. Echocardiographic parameters of WT and *Actn2* p.Met228Thr heterozygous (Het) hearts at **young age**. Values are given as mean ± SEM. No significant changes were observed between both genotypes (Mann-Whitney U test). FS – fractional shortening, LVEDD – left ventricular end diastolic dimension, LVESD - left ventricular end systolic dimension, LVAWD – left ventricular anterior wall in diastole, LVPWD – left ventricular posterior wall in diastole, LVM – calculated left ventricular mass, HR – heart rate, HW/TL – heart weight normalised to tibia length.

		**Male**		**Female**	
		**WT**	**Het**	**WT**	**Het**
Age	days	84 ± 1	84 ± 1	84 ± 1	84 ± 1
n		16	13	18	13
Body weight	g	27.4 ± 0.4	27.6 ± 0.4	20.8 ± 0.3	20.5 ± 0.3
FS	%	37.1 ± 1.6	38.0 ± 1.9	39.3 ± 1.2	38.2 ± 2.1
LVEDD	mm	3.87 ± 0.06	3.77 ± 0.08	3.58 ± 0.04	3.59 ± 0.06
LVESD	mm	2.45 ± 0.09	2.35 ± 0.12	2.18 ± 0.06	2.23 ± 0.10
LVAWD	mm	1.18 ± 0.02	1.24 ± 0.03	1.04 ± 0.04	1.00 ± 0.02
LVPWD	mm	0.86 ± 0.02	0.86 ± 0.02	0.84 ± 0.03	0.77 ± 0.02
LVM	mg	125 ± 3	126 ± 5	98 ± 3	91 ± 3
HR	bpm	504 ± 1	503 ± 2	501 ± 1	503 ± 1
n		17	16	20	14
HW/TL	mg/mm	7.22 ± 0.14	7.40 ± 0.14	5.64 ± 0.09	5.61 ± 0.10

Table S2. Echocardiographic parameters of WT and *Actn2* p.Met228Thr heterozygous (Het) hearts at **mature age**. Values are given as mean ± SEM. No significant changes were observed between both genotypes (Mann-Whitney U-test). FS – fractional shortening, LVEDD – left ventricular end diastolic dimension, LVESD - left ventricular end systolic dimension, LVAWD – left ventricular anterior wall in diastole, LVPWD – left ventricular posterior wall in diastole, LVM – calculated left ventricular mass, IVRT- Isovolumentric relaxation time, E'/A'- peak early diastolic velocity/peak later diastolic velocity of the left ventricular posterior wall, HR – heart rate, HW/TL – heart weight normalised to tibia length.

		**Male**		**Female**	
		**WT**	**Het**	**WT**	**Het**
Age	days	417 ± 4	412 ± 3	376 ± 7	384 ± 16
n		8	11	7	8
Body weight	g	45.7 ± 2.6	44.5 ± 1.7	31.2 ± 2.0	30.1 ± 1.3
FS	%	32.2 ± 3.0	31.1 ± 1.9	35.3 ± 2.2	31.4 ± 1.1
LVEDD	mm	4.09 ± 0.15	4.22 ± 0.08	3.80 ± 0.11	3.99 ± 0.09
LVESD	mm	2.79 ± 0.20	2.91 ± 0.10	2.46 ± 0.12	2.74 ± 0.10
LVAWD	mm	1.14 ± 0.09	1.08 ± 0.05	1.03 ± 0.06	0.93 ± 0.03
LVPWD	mm	1.05 ± 0.04	0.99 ± 0.06	0.90 ± 0.04	0.88 ± 0.04
LVM	mg	153 ± 13	148 ± 12	114 ± 10	111 ± 4
IVRT	ms	17.2 ± 1.1	17.0 ± 0.6	17.6 ± 0.9	18.1 ± 0.7
E'/A'		1.5 ± 0.5	1.8 ± 0.6	1.3 ± 0.2	1.5 ± 0.2
HR	bpm	476 ± 7	480 ± 9	485 ± 3	488 ± 7
n		5	9	5	6
HW/TL	mg/mm	9.1 ± 0.7	9.3 ± 0.4	6.2 ± 0.3	6.3 ± 0.2

Table S3.*Genotypes of animals at weaning or at birth.*

	Offspring					
Litter ID at weaning	M WT	F WT	M Het	F Het	M Hom	F Hom
27	0	1	2	1	0	0
28	1	1	1	1	0	0
29	0	1	2	2	0	0
30.1	0	1	0	2	0	0
30.2	1	0	1	0	0	0
31.1	2	0	2	1	0	0
31.2	0	0	1	0	0	0
32.1	0	1	0	5	0	0
32.2	1	1	2	1	0	0
32.3	0	1	2	2	0	0
34.1	2	0	1	0	0	0
34.2	0	1	4	1	0	0
34.3	0	1	0	4	0	0
35.1	0	1	2	1	0	0
35.2	1	0	1	2	0	0
35.3	1	0	3	3	0	0
36.1	3	0	2	0	0	0
36.2	0	2	3	4	0	0

total litters	M WT	F WT	M Het	F Het	M Hom	F Hom	total pups
18	12	12	29	30	0	0	83
observed	***14.5***	***14.5***	***34.9***	***36.1***	***0.0***	***0.0***	***%***
expected	***12.5***	***12.5***	***25***	***25***	***12.5***	***12.5***	***%***

	Offspring		
Litter ID at weaning	WT	Het	Hom
27	1	3	0
28	2	2	0
29	1	4	0
30.1	1	2	0
30.2	1	1	0
31.1	2	3	0
31.2	0	1	0
32.1	1	5	0
32.2	2	3	0
32.3	1	4	0
34.1	2	1	0
34.2	1	5	0
34.3	1	4	0
35.1	1	3	0
35.2	1	3	0
35.3	1	6	0
36.1	3	2	0
36.2	2	7	0
at birth			
39	2	2	0
40	0	6	0
41	2	2	0
42	1	4	0

Table S4. ***Genotypes of embryos at E15.5.***

litter harvest date	WT	Het	Hom
12/08/2020	2	4	0
12/08/2020	1	6	1
14/08/2020	1	6	0
14/08/2020	1	7	0
14/08/2020	3	3	1
26/08/2020	2	4	4
27/08/2020	3	6	2
09/10/2020	1	5	0
16/10/2020	3	4	0
30/10/2020	2	2	4
20/11/2020	3	4	3
03/02/2021	4	2	2
10/02/2021	0	2	4
25/02/2021	3	4	1
17/03/2021	2	6	1
18/03/2021	4	3	4
28/04/2021	4	3	3
28/04/2021	2	2	0
05/05/2021	0	2	1
12/05/2021	1	4	3
13/05/2021	3	5	2
10/06/2021	2	6	3
17/06/2021 A	2	3	2
17/06/2021 B	0	4	2
07/07/2021	3	3	3
08/07/2021	3	4	2
10/09/2021	3	5	0
16/09/2021	1	3	4
17/09/2021	2	5	2
22/09/2021	2	6	2

total litters	WT	Het	Hom	total pups
30	63	123	56	242
observed	***26.03***	***50.83***	***23.14***	***%***
expected	***25***	***50***	***25***	***%***

Table S5. Nuclear parameter of WT and *Actn2* p.Met228Thr homozygous (Hom) embryonic hearts (E15.5). Values are given as mean ± SEM. A total of 112 images from 23 WT hearts and 79 images from 16 Hom hearts were analysed; nested ANOVA. Significant differences are highlighted in bold. For an explanation of parameters see https://cellprofiler.org/

**Parameter**	**WT**	**Hom**	***P* value**
**Number per field**	**72.17 ± 1.58**	**60.91 ± 1.71**	**0.0092**
Area (μm^2)	101.83 ± 1.20	104.25 ± 1.33	0.1717
Perimeter (μm)	35.71 ± 0.21	36.14 ± 0.23	0.1671
Feret's diameter (μm)	13.37 ± 0.15	13.51 ± 0.22	0.7563
Length (μm)	11.37 ± 0.07	11.50 ± 0.07	0.1671
Width (μm)	5.71 ± 0.03	5.72 ± 0.04	0.8879
**Eccentricity**	**0.74 ± 0.003**	**0.69 ± 0.04**	**<0.0001**
Solidity	0.93 ± 0.001	0.93 ± 0.01	0.7727

Tables S6 and S7 supplied as extra files.

Table S8. Proteins related to protein turnover with $p < 0.05$, sorted by fold change (FC).

	Group	Protein	Full Name	FC	P-Value
Upregulated	Protein Turnover	Psmd7	Proteasome 26S Subunit, Non-ATPase 7	1.28	0.01
		Psma7	Proteasome 20S Subunit Alpha 7	1.28	0.02
		Rps2	Ribosomal Protein S2	1.26	0.04
		Psmd6	Proteasome 26S Subunit, Non-ATPase 6	1.25	0.01
		Otub1	OUT Deubiquitinase, Ubiquitin Aldehyde Binding 1	1.19	0.01
		Nedd4	NEDD4 E3 Ubiquitin Protein Ligase	1.14	0.03
		Skp1	S-Phase Kinase Associated Protein 1	1.14	0.002
		Rps3	Ribosomal Protein S3	1.14	0.04
		Rpl11	Ribosomal Protein L11	1.12	0.003
		Eif3a	Eukaryotic Translation initiation Factor 3 Subunit A	1.12	0.05
		Uba1	Ubiquitin Like Modifier Activating Enzyme 1	1.1	0.04
		Rpl23	Ribosomal Protein L23	1.09	0.05
		Uchl1	Ubiquitin C-Terminal Hydrolase L1	1.06	0.01
		Npm1	Nucleophosmin 1	1.06	0.04
		Rpl5	Ribosomal Protein L5	1.06	0.01
		Vps29	VPS29 Retromer Complex Component	1.05	0.01
		Ube2l3	Ubiquitin Conjugating Enzyme E2 L3	1.05	0.02
		Aimp2	Aminoacyl TRNA Synthetase Complex Interacting Multifunctional Protein 2	1.05	0.02
		Psmc2	Proteasome 26S Subunit, ATPase 2	1.05	0.05
		Psmc5	Proteasome 26S Subunit, ATPase 5	1.05	0.04
		Psme1	Proteasome Activator Subunit 1	1.03	0.02
		Hsp90ab1	Heat Shock Protein 90 Alpha Family Class B Member 1	1.01	0.03

	Group	Protein	Full Name	FC	P-Value
Downregulated	Protein Turnover	Psmb4	Proteasome 20S Subunit Beta 4	0.97	0.03
		Ppia	Peptidylprolyl Isomerase A	0.94	0.01
		Psmb6	Proteasome 20S Subunit Beta 6	0.94	0.001
		Eif3m	Eukaryotic Translation initiation Factor 3 Subunit M	0.84	0.05
		Eif4g1	Eukaryotic Translation initiation Factor 4 Gamma 1	0.83	0.04
		Psmd3	Proteasome 26S Subunit, Non-ATPase 3	0.81	0.04
		Eif3b	Eukaryotic Translation initiation Factor 3 Subunit B	0.8	0.02
		Cdk1	Cyclin Dependent Kinase 1	0.78	0.01
		Cops2	COP9 Signalosome Subunit 2	0.72	0.03

Table S9. Reagents used for qPCR, Western blotting (WB) and immuno-fluorescence (IF)

a) Taqman Assays for qPCR (all Applied Biosystems, FAM-MGB unless stated otherwise)

Transcript	*Species*	*Assay ID*	*Comments*
Acta1	Mouse	Mm00808218_g1	
Actc1	Mouse	Mm01333821_m1	
Actn1	Mouse	Mm01304398_m1	
Actn2	Mouse	Mm00473657_m1, Mm01340071_m1, Mm01340076_mH	
Actn3	Mouse	Mm00496495_m1	
Actn4	Mouse	Mm00502489_m1	
Ankrd1	Mouse	Mm00496512_m1	
Ankrd2	Mouse	Mm00508030_m1	
Anln	Mouse	Mm00503748_m1	
Cdkn1a	Mouse	Mm04205640_g1	
Cdkn1b	Mouse	Mm00438168_m1	
Cdkn2a	Mouse	Mm00494449_m1	
Fhl1	Mouse	Mm04204611_g1	
Fhl2	Mouse	Mm00515781_m1	
Gapdh	Mouse	4352339E	VIC-MGB labelled
Myh7	Mouse	Mm00600555_m1	
Nppa	Mouse	Mm01255748_g1	
Nppb	Mouse	Mm01255770_g1	
Rcan1.4	Mouse	Mm00627762_m1	
Trp53	Mouse	Mm01731290_g1	
Wee1	Mouse	Mm00494175_m1	

b) Antibodies (Dilutions for Western blotting unless stated.)

Protein	*Type*	*Clone/Cat. No.*	*Source*	*Dilution (WB)*
alpha-Actinin2	rabbit	ab68167	Abcam	1:10,000, 1:200 (IF)
alpha-Actinin2	mouse	EA53/ A7811	Sigma	1:500 (IF)
alpha-Actinin3	mouse	ab68204	Abcam	1:1000
beta-Myosin	rabbit	22280-1-AP	Proteintech	1:2000
Gapdh	rabbit	ABS16	Millipore	1:3000
Hsp27	rabbit	2442	CST	1:1000
HspB7	rabbit	15700-1-AP	Proteintech	1:1000
Laminin	rabbit	L9393	Sigma	1:200 (IF)
LC3	rabbit	14600-1-AP	Proteintech	1:2000
Myosin heavy chain	mouse	A4.1025	Developmental Studies Hybridoma Bank	1:5 (IF)
p53	mouse	DO1 supernatant	gift by A. Turnell	1:20
p62	rabbit	66184-1-Ig	Proteintech	1:2000
phospho-H3 (T3)	rabbit	07-424	Upstate/Sigma	1:500 (IF)
phospho-H3 (S10)	rabbit	PA5-17869	Thermo	1:100 (IF)
Titin T12	mouse	T12 (near Z-disc)	[*]	1:10 (IF)
Ubiquitin	rabbit	Z0458	DAKO	1:1000

[*] D.O. Furst, M. Osborn, R. Nave, K. Weber, The organization of titin filaments in the half-sarcomere revealed by monoclonal antibodies in immunoelectron microscopy: a map of ten nonrepetitive epitopes starting at the Z line extends close to the M line, J Cell Biol 106(5) (1988) 1563-72.

Original blots

Figure 7B (both flipped)

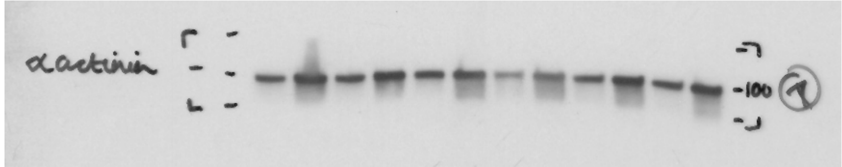

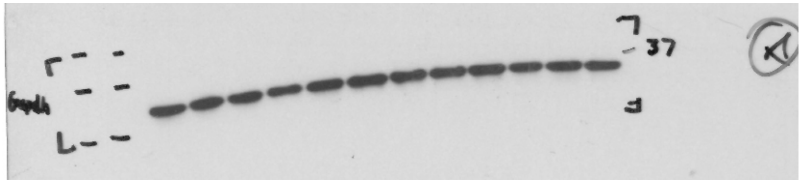

ES5 (membrane cut into 4 strips incubated with different antibodies)

Figure S4A

Actn2

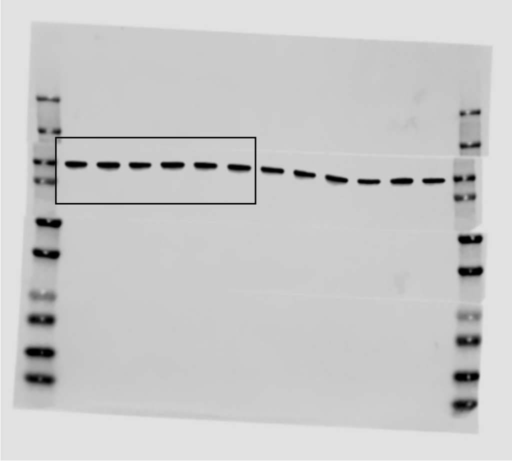

Gapdh

HspB7

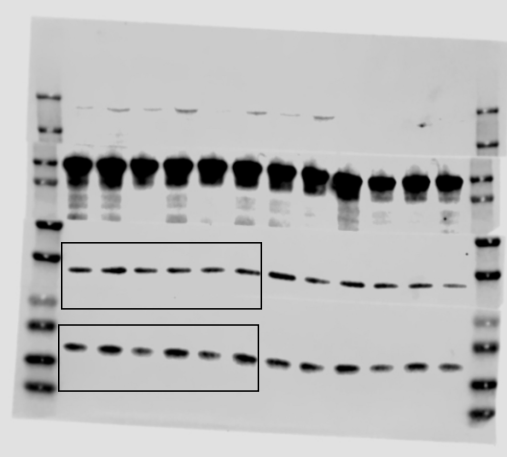

Myh7

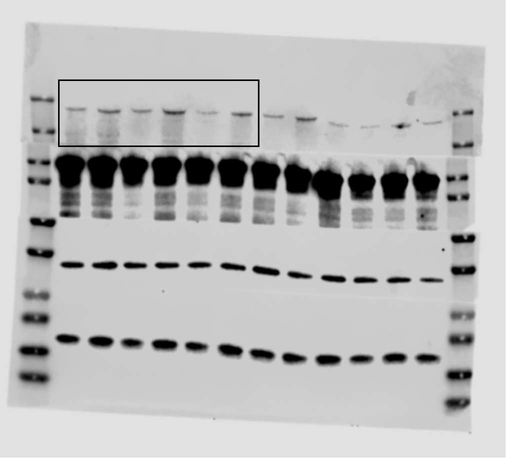

KW2 (membrane cut into 4 strips incubated with different antibodies)

Hsp27

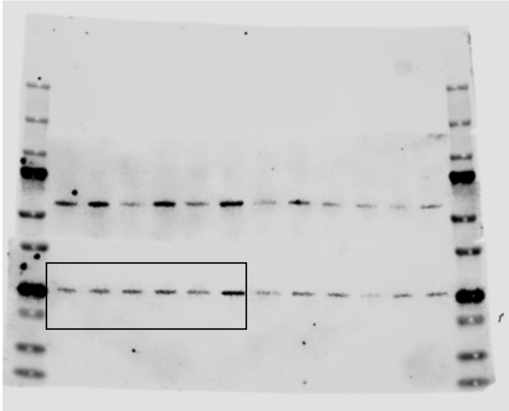

KW3 (membrane cut into 3 strips incubated with different antibodies)

Original blots

Figure S4C

ubiquitin

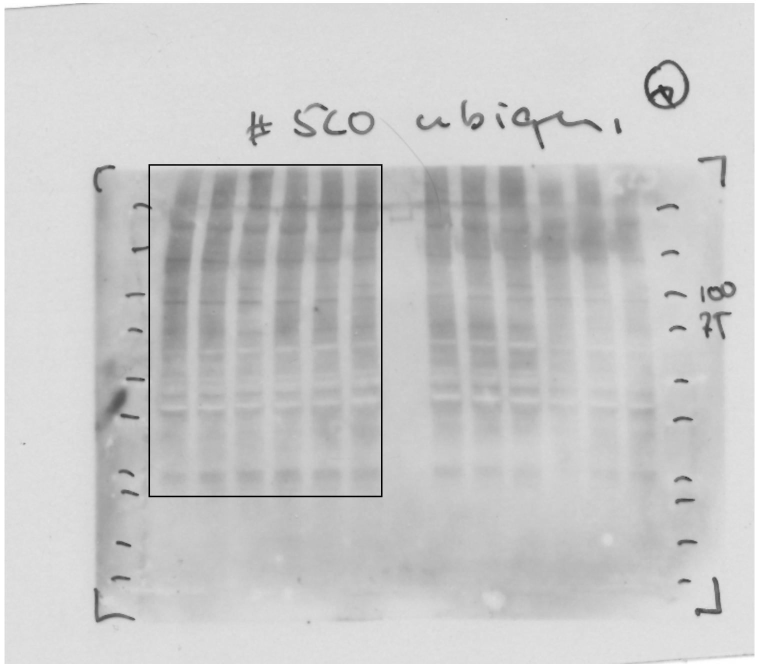

520

Gapdh (2nd incubation)

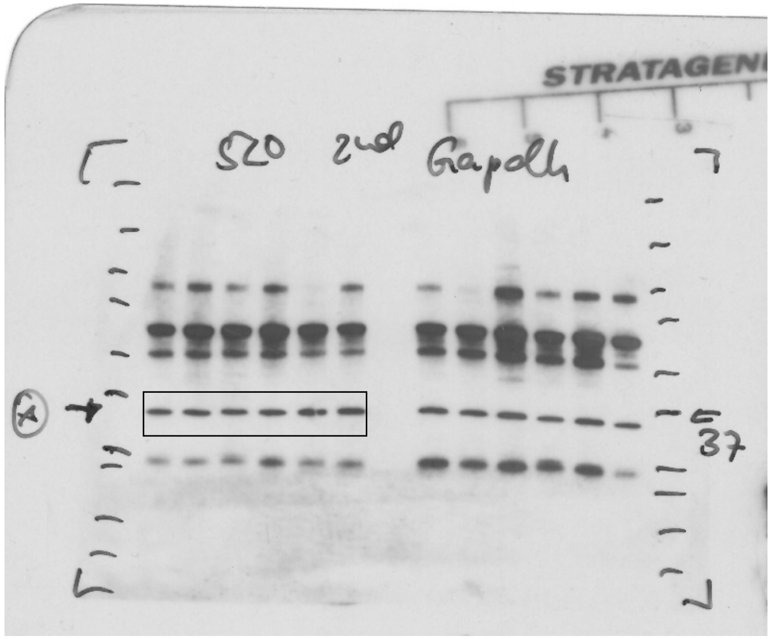

Original blots

Figure S19B

Actn3

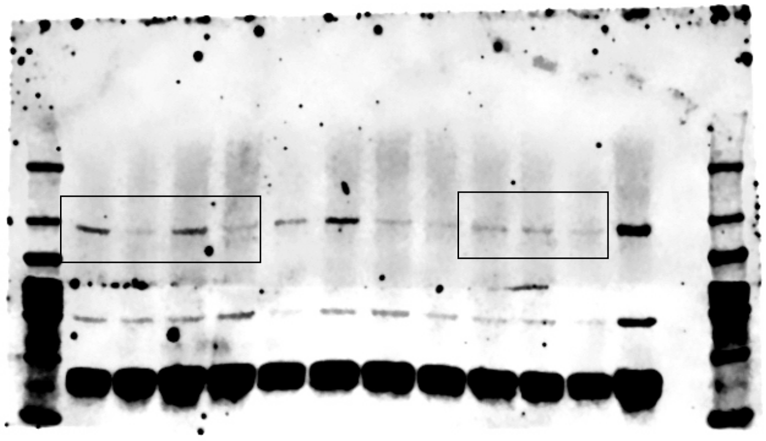

Gapdh

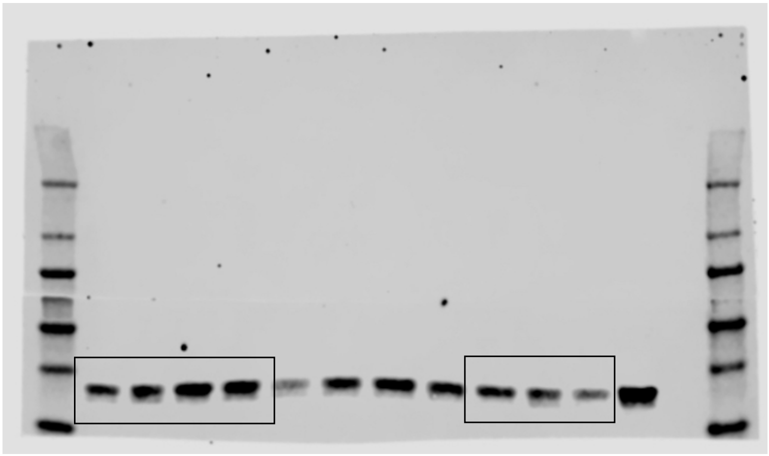

KW14 (membrane cut into 2 strips incubated with different antibodies)

Figure S20B

(ES6)

Ubiquitin

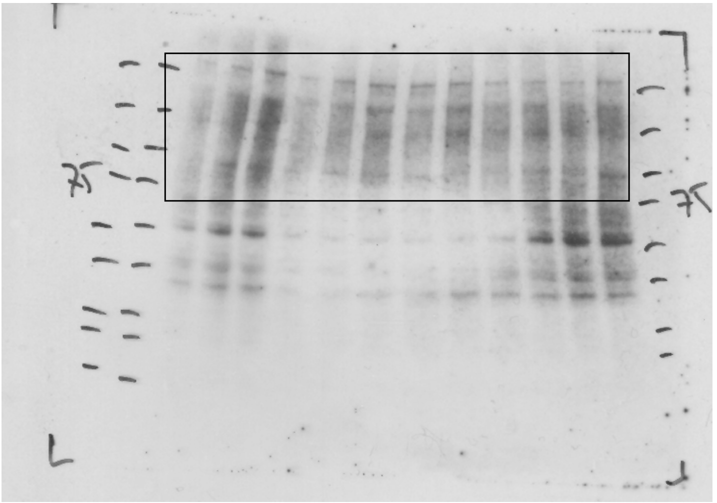

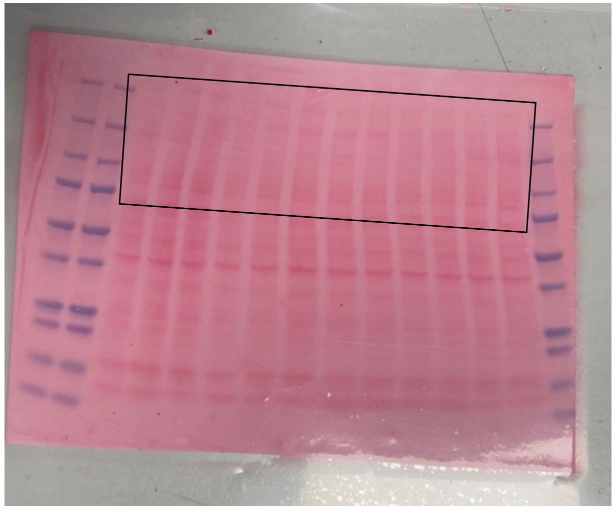

PonceauS

Original blots

Figure 16B

p53

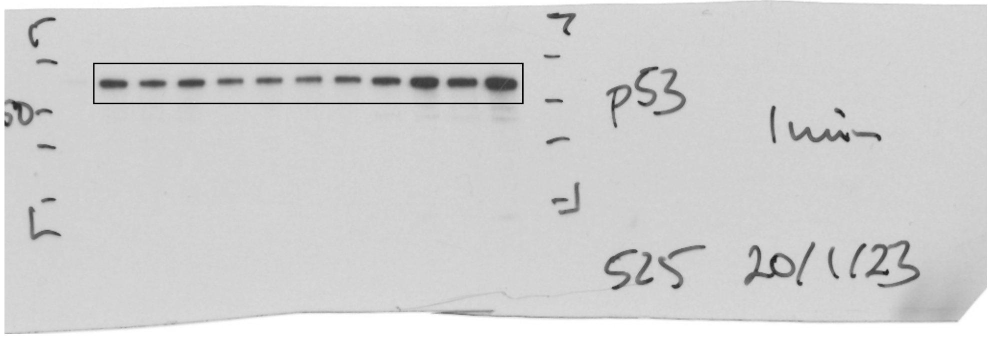

PonceauS for cardiac actin

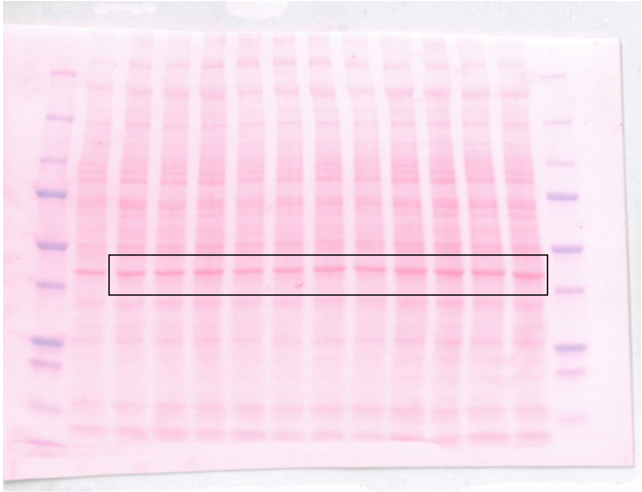

Figure S21A (all flipped)

p62

LC3

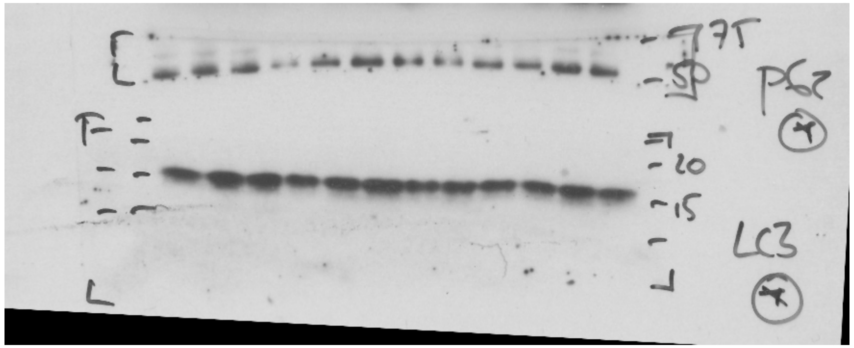

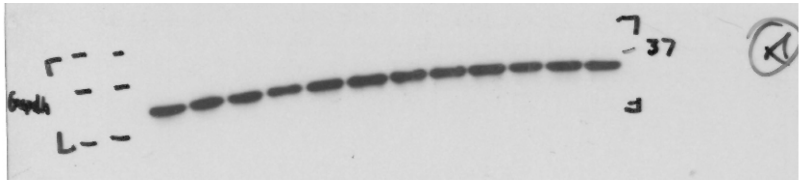

Gapdh (same as Figure 7B)

ES5 (membrane cut into 4 strips incubated with different antibodies)
