# Supplementary material for: Insights into the Role of a Cardiomyopathy-Causing Genetic Variant in ACTN2
Source: Cells. 2023 Feb 24;12(5):721. doi: 10.3390/cells12050721 (PMC10001372; doi:10.3390/cells12050721)
Supplement: Supplementary file 1 [file cells-12-00721-s001.zip › Table S6.pdf]

	Gene Names	log2(FC)	P value	WT 1	WT 2	WT 3	WT 4	WT 5	WT 6	HOM 1	HOM 2	HOM 3	HOM 4	HOM 5	HOM 6	WT Avg	HOM Avg	Fold change
1	Aars	0.31	0.033	154.1	148.6	160.4	156.8	159.65	234.3	169.85	188.95	236.35	240.55	231.9	187.2	169.0	209.1	1.24
2	Aco2	0.12	0.066	195.6	171.9	169.3	155.6	195.2	153.3	167.8	158.4	201.1	208.7	207.6	186.1	173.48	188.28	1.09
3	Actc1	0.26	0.013	255.9	242.3	245.8	265.6	297	239.6	249.2	273.4	340.6	307.2	341.2	341.7	257.7	308.9	1.20
4	Adk	0.23	0.021	249.25	250.45	253.55	244.45	259.25	254.9	285.75	274.75	271.3	304.2	313.15	329.6	252.0	296.5	1.18
5	Adss1	0.16	0.039	302.1	254.6	263.2	267.9	290.1	271.4	289.1	264.2	341.8	302.1	316	330.3	274.9	307.3	1.12
6	Agl	0.15	0.019	512.5	467.2	462.7	476.6	454.4	468.9	487.5	494	596.2	514.1	515.2	543.7	473.7	525.1	1.11
7	Ahsa1	0.00	0.047	185.95	194.2	192.3	202.75	194.05	199.65	199	207.85	173.05	199.05	193.45	196.5	194.82	194.82	1.00
8	Aimp2	0.07	0.021	142	139.65	146.15	146.75	163.65	140.3	144.4	140.4	154.55	157.35	161.5	163	146.4	153.5	1.05
9	Ak1	0.34	0.018	265.8	249.3	259.4	257	251.9	238	248.3	255.2	340.3	362.4	363	359.5	253.6	321.5	1.27
10	Aldoa	0.06	0.066	240.75	240.35	219	242.25	247.4	239.95	242.05	248.2	253.6	239.15	247.3	255	238.28	247.55	1.04
11	Anp32a	0.63	0.021	133.2	120.75	122.3	127	130.35	119.05	132.55	126.6	202.2	214.75	248.4	237.85	125.4	193.7	1.54
12	Anxa2	0.29	0.007	192.3	192.2	183.1	200.1	208.8	179.1	221.1	225.4	247.6	238	248	237.3	192.6	236.2	1.23
13	Anxa5	0.23	0.022	159.5	158.1	160.65	168.85	157.55	156.65	180.15	179.25	199	190.35	186.25	189.85	160.2	187.5	1.17
14	Apex1	0.27	0.000	421.4	424.9	394.6	465.6	418.7	445.5	501.6	474.9	504.5	521.9	546.8	551.7	428.5	516.9	1.21
15	Apoe	0.21	0.007	205.1	209.25	223.9	220.25	210.7	254.35	280.05	262	285.5	246.5	251.95	205.6	220.6	255.3	1.16
16	Arcn1	0.29	0.048	244.5	219.7	229.2	232.5	244.2	202.4	215.4	216.8	276.1	319	313.8	337.2	228.8	279.7	1.22
17	Atad1	0.19	0.032	182.6	166.2	198.9	176.4	193	185.8	191.9	180.8	202	216.3	243.4	225.8	183.8	210.0	1.14
18	Atic	0.18	0.024	303.1	292.9	301.9	284.4	304.7	303.8	314.1	315.3	330.4	345.4	349	374.5	298.5	338.1	1.13
19	Atp1b1	0.04	0.006	555.6	489.3	563	533.5	564.5	496.5	546.9	513.4	539	551	570.6	563.1	533.7	547.3	1.03
20	Atp5po	0.07	0.063	119.3	116.2	119.8	114	114.7	112.8	105.5	101.2	121	135	131.5	136.7	116.13	121.82	1.05
21	Atpif1	0.28	0.005	74.8	79.2	64.1	68.8	71	66.5	91.1	70.6	94.9	86.1	91.1	81.2	70.7	85.8	1.21
22	Banf1	1.01	0.016	145.4	111.3	130.3	118.8	185.4	100.9	140.5	115.7	294.2	361.3	342.8	345	132.0	266.6	2.02
23	Bola1	0.15	0.003	252.5	265.9	246.8	271.1	241.9	258.9	265.7	281.9	300.2	286.7	292.3	273.8	256.2	283.4	1.11
24	Bsg	0.07	0.023	380.05	415.75	392.9	405.6	427.55	388.45	439.45	440.55	384.8	424	414.4	431.35	401.7	422.4	1.05
25	Cab39	0.55	0.000	98.5	98.4	88.9	97.9	94.6	85.5	128.5	141.5	146.8	128.4	138.1	141.3	94.0	137.4	1.46
26	Cald1	0.46	0.001	24.4	33	25.6	35.2	31.7	27.8	38.2	33.1	41.7	42	46.1	43.1	29.6	40.7	1.37
27	Calml3	0.12	0.004	179.1	152.55	188.85	181.9	196.2	180.55	184.05	168.6	201.5	194.6	194.1	227	179.9	195.0	1.08
28	Calr	0.25	0.007	249.75	243.95	247.45	274.9	278.65	264.35	300.85	287.4	287	325.4	318.15	333.75	259.8	308.8	1.19
29	Canx	0.19	0.085	176.25	178.55	179.4	186.3	189.65	188.65	217.65	205.15	191.35	204.25	209.5	221.35	183.13	208.21	1.14
30	Casq2	0.88	0.009	150.5	192	165	162.7	166.3	152.1	182.9	177.1	415.9	360.8	348.7	335.8	164.8	303.5	1.84
31	Cct3	0.21	0.015	192.45	223.05	207.05	210.55	214.25	218.6	221.65	232.55	243.25	241.9	255.3	266.45	211.0	243.5	1.15
32	Cct8	0.21	0.025	232.6	225.2	206.8	219.4	215.8	219.9	240	219.2	258.7	245.1	268.4	291.5	220.0	253.8	1.15
33	Chchd3	0.04	0.016	260	253.75	252.95	249.2	248.15	232.55	262.3	247.05	261.1	250.75	262.35	259.25	249.4	257.1	1.03
34	Ckap4	0.25	0.004	173.6	160.5	179	180.3	209.5	171.3	207.8	193.6	214.9	225.3	216.8	222	179.0	213.4	1.19
35	Clic4	0.31	0.005	206.4	191.5	203.45	204.45	214.1	197.4	212.65	210.85	248.95	301.35	276.15	258.35	202.9	251.4	1.24
36	Colgalt1	0.13	0.019	170.3	175	177.1	166.2	165.8	165	190.8	160.2	194	187.1	188.5	193.3	169.9	185.7	1.09
37	Copa	0.23	0.044	249.6	181.6	232.2	212.2	251.6	196.2	259.6	201.9	272.5	266.7	277.5	274.1	220.6	258.7	1.17
38	Copb1	0.09	0.013	278.3	271.7	276.6	282.9	301.4	267	304.6	301.4	286.3	291.5	309.6	294.6	279.7	298.0	1.07
39	Cpt1b	0.19	0.043	157.8	152	155.6	147.4	152.2	135.1	144.1	155.1	178.4	162.4	189	198	150.0	171.2	1.14
40	Crip2	0.50	0.030	166.4	142.5	159	149.3	159.1	151.7	150	151.9	260.2	259.2	259.2	233.9	154.7	219.1	1.42
41	Cryab	0.08	0.001	193.1	209.35	200.85	204.25	179	203.2	244.35	254.75	208.35	186.1	186.1	175.15	198.3	209.1	1.05
42	Csrp2	0.16	0.001	298.3	303.6	286.8	292.9	297.3	311.4	354.4	326.7	306.3	329.2	348.2	330.4	298.4	332.5	1.11

	Gene Names	log2(FC)	P value	WT 1	WT 2	WT 3	WT 4	WT 5	WT 6	HOM 1	HOM 2	HOM 3	HOM 4	HOM 5	HOM 6	WT Avg	HOM Avg	Fold change
43	Csrp3	0.50	0.005	187.5	170.7	166.8	166.3	171.4	166.1	181.2	204.1	279.1	264.9	238.2	282.6	171.5	241.7	1.41
44	Ctnnb1	0.15	0.019	128.65	141.4	139.15	131.5	144.7	131.2	140.6	148	145.9	161.6	157.8	149.1	136.1	150.5	1.11
45	Ctsb	0.33	0.000	103.3	102.5	104	107.7	112.7	112.4	122.6	127.6	137.9	140.6	148.5	130.2	107.1	134.6	1.26
46	Ctsd	0.44	0.012	115.5	110.8	122.9	120.5	137.6	111.6	127.4	125.3	156.2	166.8	190.2	210.6	119.8	162.8	1.36
47	Ddah2	0.16	0.013	167.3	177.25	181.35	171.4	191.15	184.55	184.1	192.25	197	207.1	209.6	209.25	178.8	199.9	1.12
48	Ddb1	0.38	0.010	200.9	195.8	196.1	202.9	229.5	204.5	207.5	221.6	291.5	280.3	296.4	307.6	205.0	267.5	1.31
49	Ddx1	-0.01	0.008	386.55	377.45	403.55	417.9	435.8	432.65	422.15	410.9	387.8	415.4	408.05	400.45	409.0	407.5	1.00
50	Des	0.24	0.004	173.95	167	179.2	184.75	189.6	162.35	204.6	211.2	201.9	202.7	209.8	215.85	176.1	207.7	1.18
51	Dhx29	2.65	0.009	32.8	22.9	29.6	29.7	42.7	26.3	41.5	33.5	241.7	258.2	287.8	293.2	30.7	192.7	6.28
52	Dpf2	0.40	0.041	11.1	11.4	13.2	16.8	13.6	19.8	19.3	15.4	17.2	20.9	24.3	16.1	14.3	18.9	1.32
53	Dpysl2	0.11	0.009	207	206.65	200.15	214.65	211.65	222.75	228.7	239.8	214.65	224.75	230.65	222.9	210.5	226.9	1.08
54	Dync1li1	0.25	0.002	152.5	152.5	144.2	148.6	126.2	159.4	152.9	178.1	178.5	171.4	182.9	183.3	147.2	174.5	1.19
55	Ech1	0.15	0.017	285.8	269.45	282.35	276.65	299.85	277.45	274	288.35	298.5	332.8	348.5	337.1	281.9	313.2	1.11
56	Eef1b	0.34	0.029	119.15	105.35	112.95	125.15	119.4	129.15	123.6	122.45	147.45	167.9	174.2	165	118.5	150.1	1.27
57	Eef1d	0.14	0.039	412.8	393.1	424.1	438.7	431.9	435.8	440.2	435.3	432.9	466.4	495.5	527.3	422.7	466.3	1.10
58	Eef1g	0.18	0.050	168.05	169.9	154.25	180.1	165.35	169.7	185.4	169.5	192.7	193.2	202.45	198.65	167.9	190.3	1.13
59	Eef2	0.31	0.074	159.1	132.6	146.8	154.1	160.1	143.9	165.8	156.1	187.1	199.7	199.2	204.4	149.43	185.38	1.24
60	Ehd4	0.13	0.013	238.7	216.4	235.6	240.2	257.8	249.3	270.2	271.9	259.5	246.4	250.9	274.9	239.7	262.3	1.09
61	Eif3a	0.17	0.046	99.2	117.5	105.7	119.4	115.3	121.5	117.5	132.3	133.2	116.7	127	135.9	113.10	127.10	1.12
62	Eif4g2	0.20	0.015	100.4	110.3	88.3	108.7	107	109.9	123.5	105.9	124.9	120.3	132.8	110.9	104.1	119.7	1.15
63	Elavl1	0.22	0.005	199.85	196.05	193.7	205.55	212.35	216.6	215.85	214.65	259.1	239.9	255.45	242.15	204.0	237.9	1.17
64	Eno1	0.19	0.028	295.8	289.1	274.4	285.8	295.8	278.9	307.6	308.4	319.8	329.6	330.3	363.6	286.6	326.6	1.14
65	Erh	0.10	0.006	318.1	311.7	300.3	297.7	311.5	316.1	342.4	316.4	318.9	326.5	332.2	349.1	309.2	330.9	1.07
66	Fabp5	0.18	0.014	154.9	158.15	157.45	152.4	156.2	172.35	179	177.35	174.95	178.1	181.8	188.6	158.6	180.0	1.13
67	Fam162a	0.30	0.020	135.2	124.1	148.2	135.7	128.4	120.9	145	146.8	193.7	175	162.2	154.4	132.1	162.9	1.23
68	Fermt2	0.06	0.040	384.1	376.6	395.6	373.2	395.9	398.1	397.9	388.7	417.7	392.6	404.9	412.1	387.3	402.3	1.04
69	Fhl1	0.20	0.001	250.2	266.4	248.6	283.8	272.8	282.5	288	312.1	312	291.7	310.2	332.3	267.4	307.7	1.15
70	Filip1	0.29	0.001	248.1	258.5	256.2	262.5	273.65	258.9	302.25	283.7	315.4	327.1	340.5	339.8	259.6	318.1	1.23
71	Fkbp1a	0.16	0.004	255.9	253.4	248.6	252.3	291.1	274.8	289	297.9	291.3	283.9	312.7	282.7	262.7	292.9	1.12
72	Fkbp3	0.17	0.040	358.4	331.65	344.7	344.85	369.85	344.8	343.05	340	411.65	411.3	430.85	427.15	349.0	394.0	1.13
73	Flii	0.25	0.002	236.5	225.3	231.5	238.4	238.9	251.8	263.4	260.5	279.1	262.2	308.5	312.5	237.1	281.0	1.19
74	Flnc	0.05	0.050	208.8	213.9	189.6	197	196.3	209.8	218.7	227.6	218.7	197.8	193.5	201.4	202.6	209.6	1.03
75	Fscn1	0.17	0.000	205.55	202.65	214.45	225.95	216.7	214.15	241.25	236.2	240.05	231.75	242.6	249.4	213.2	240.2	1.13
76	Fubp1	0.17	0.014	215.25	223.55	219.15	232.35	219.05	211.05	231.85	237.4	249.25	252.65	252.35	259.5	220.1	247.2	1.12
77	Fxr1	0.00	0.015	335.8	350.7	361.7	328.15	351.5	334.75	330.5	339.75	314.6	340.4	365.2	366.75	343.8	342.9	1.00
78	Gapdh	0.01	0.020	278.65	249.25	258.95	258.95	298	247.05	258.55	255.05	268.6	261	278.35	278.55	265.1	266.7	1.01
79	Gemin5	0.41	0.011	48.9	48.4	46.5	56.6	47.8	48	54.3	51.3	72.4	60.3	82.5	73.8	49.4	65.8	1.33
80	Glud1	0.21	0.021	133	155.3	130.3	140.5	132	138.3	136.4	149.5	153.1	164.5	183.5	172.9	138.2	160.0	1.16
81	Gm20431	0.12	0.030	497	471	481.6	484.4	499.6	473.2	492.5	501.7	493.3	545.2	533.9	592.3	484.5	526.5	1.09
82	Gm45713	0.18	0.022	212.85	216.85	205	209.55	217	208.85	229.25	220.7	255.8	231.6	247.55	253.4	211.7	239.7	1.13
83	Gmpr	0.67	0.009	39.3	32.5	36.8	35.9	40.7	24.6	39.1	35.7	67.3	64.1	67.6	60.4	35.0	55.7	1.59
84	Gnai2	0.07	0.018	206.7	205.4	230.6	225.4	256.5	219.4	249.3	234.2	217.1	256.3	231.5	224.4	224.0	235.5	1.05

	Gene Names	log2(FC)	P value	WT 1	WT 2	WT 3	WT 4	WT 5	WT 6	HOM 1	HOM 2	HOM 3	HOM 4	HOM 5	HOM 6	WT Avg	HOM Avg	Fold change
85	Got2	0.09	0.064	294.5	264.8	315.7	272.6	353	268.3	311	252.9	323.4	327.5	326.7	345.3	294.82	314.47	1.07
86	Gpi	0.17	0.023	166.05	153.6	159.55	157	184.85	160.05	190.1	173.3	184.6	193.7	169.45	191.55	163.5	183.8	1.12
87	Gstm4	0.23	0.020	254.2	230	223.2	223.3	226.5	291.3	263.1	252.7	326.4	292.1	291.7	277.8	241.4	284.0	1.18
88	Gstp2	0.09	0.023	308.35	305.15	310.05	322.6	287.1	299.45	345.35	321	314.15	308.85	320.45	334.65	305.5	324.1	1.06
89	Gyg	0.41	0.012	147.1	217.6	169.8	175.5	138.6	226.1	150.3	244.4	250.8	268.9	250.6	259.6	179.1	237.4	1.33
90	H1-1	0.26	0.017	158.25	136.95	122.3	162.15	134.05	130.2	137.1	123.2	200.55	181.65	183.35	187.7	140.7	168.9	1.20
91	H2bc3	0.32	0.036	255.45	248.15	244.85	248.65	296.95	229.2	255.4	237.85	344.15	361.85	349.35	351.35	253.88	316.66	1.25
92	Hadhb	0.07	0.065	170	166.7	176.2	167.1	162.3	169.4	155.6	148.5	197.6	182.8	214.2	161.7	168.62	176.73	1.05
93	Hbb-y	0.40	0.044	243.6	191.5	215.2	211.7	282.9	333.2	309.3	272.9	356.9	342.6	346.3	328.6	246.4	326.1	1.32
94	Hdgf	0.79	0.010	237.8	194.9	211.5	235.15	249.9	226.9	250.75	222.3	450.4	452.6	481.7	491.1	226.0	391.5	1.73
95	Hint1	0.21	0.020	235.9	222	207.2	253	251.6	236.2	249.3	249.2	273.8	266.4	269.8	312.1	234.3	270.1	1.15
96	Hk1	0.01	0.021	203	207.3	203.8	190	213.2	204	197.1	192	207.8	219.9	211.9	201.8	203.6	205.1	1.01
97	Hnrnpa0	0.16	0.034	163	157.15	155.15	170.65	176.15	154.8	161.9	164.15	180	187.95	197.75	203.5	162.8	182.5	1.12
98	Hnrnpk	0.02	0.026	160.9	141.5	156.8	159.8	172.5	177.1	155.3	149.3	145.7	176.1	177.5	175.4	161.4	163.2	1.01
99	Hnrnpll	0.16	0.001	194	201.6	206.1	206.6	201.4	198.3	211.8	222.9	235.6	229.2	214.3	239	201.3	225.5	1.12
100	Hprt1	0.41	0.120	133.05	121.1	122.55	125.4	127.45	115.25	118.65	126.3	183.75	184.35	180.25	195.3	124.13	164.77	1.33
101	Hsd17b10	0.11	0.053	131.9	134.05	132.75	127.2	131.7	129.4	131.35	133.1	141.5	146.2	147.1	148.75	131.17	141.33	1.08
102	Hsp90ab1	0.02	0.032	221.3	220.4	219.7	222.2	235.75	229.05	236.8	232.65	208.2	221.65	223	246.3	224.7	228.1	1.01
103	Hspa8	0.07	0.017	206.15	202.1	200.5	215.1	241.7	208.75	236.25	217.4	216.5	214.1	231.15	224.4	212.4	223.3	1.05
104	Hspb1	0.24	0.006	216.3	184.85	198.2	194.85	199	196.1	221.65	209.7	237.65	239.7	235.95	260.5	198.2	234.2	1.18
105	Hspd1	0.06	0.024	550.5	543.9	549.1	590.7	544.5	558.9	555.5	561.5	558.1	599	580	616	556.3	578.4	1.04
106	Hyou1	0.12	0.142	162.9	190.15	193.45	184.5	189.8	190.55	202.25	188.5	197.65	205.4	205.2	209.95	185.23	201.49	1.09
107	Idh3a	0.08	0.031	145.1	149.6	147.3	135.7	144.3	136.5	139.5	144.6	162.7	148.7	162.7	152.3	143.08	151.75	1.06
108	Ilf3	0.16	0.012	150.2	164.8	155.8	164.5	166.3	171.6	173.3	176.5	175.6	167.3	197.8	198.4	162.2	181.5	1.12
109	Immt	0.02	0.034	541.1	561.9	543.1	531.9	561.2	567.9	545.9	530.1	552.4	568.9	601.6	564.2	551.2	560.5	1.02
110	Ipo5	0.22	0.072	204.8	190.7	208.2	220.1	216.1	231.1	228.4	221.9	296.2	257	256.8	222	211.83	247.05	1.17
111	Ipo7	0.20	0.019	228.2	206.35	217.05	224.5	232.25	225.85	220.9	220.2	263.75	270.95	269.75	285.65	222.4	255.2	1.15
112	Lgals1	0.20	0.000	223.7	212.8	215.7	253.5	243.5	191.2	241.1	255.4	272.5	239.6	274	261.4	223.4	257.3	1.15
113	Lman1	0.25	0.013	146.1	133.6	132.1	128.1	138.5	156.7	160.6	143.3	171.7	150.8	169	194.7	139.2	165.0	1.19
114	Luc7l2	0.24	0.026	316.1	297.5	273	297.7	290.3	289.8	297.1	281.8	362.6	359	403.5	375.2	294.1	346.5	1.18
115	Map1b	0.48	0.007	125	117.9	115	145.1	126.7	139.4	142.6	127.5	193.7	205	205.2	198.7	128.2	178.8	1.39
116	Map3k20	0.16	0.008	172	162.1	185.4	181.4	174.6	173.8	176.8	190.1	198.6	188.8	205	212.3	174.9	195.3	1.12
117	Map4	0.09	0.013	294.7	302.75	290.95	306.5	302	293.7	310.55	308.25	326.8	320.1	318.1	325.65	298.4	318.2	1.07
118	Mapre2	0.24	0.040	116.4	104.2	121.4	121.7	144.7	138.7	129.9	122.2	146.1	154	162.8	165.8	124.5	146.8	1.18
119	Marcks	0.32	0.005	106.3	135	113.5	152.9	147.9	135.5	170.2	166.8	152	154.3	159.7	186.8	131.9	165.0	1.25
120	Matr3	0.00	0.045	159.1	166.45	164.2	163.35	152.55	168.35	157.6	153.5	176.15	156.2	162.25	168.5	162.33	162.37	1.00
121	Mdh1	0.24	0.058	163.1	142.6	147.9	147.9	158.1	141.5	135.4	150.2	199.2	195.4	195.8	191.8	150.18	177.97	1.18
122	Mest	0.29	0.002	89	108	114.8	102.3	115.7	117	126.8	124.3	125.7	136.3	147.6	131.3	107.8	132.0	1.22
123	Myef2	0.31	0.001	183.1	200.2	181.5	204.2	211.6	199.3	244.5	214.1	229.6	241	273.6	260	196.7	243.8	1.24
124	Myom1	0.00	0.020	313.65	298.8	307.2	302.35	295.1	301.1	321.95	328.85	296.9	290.2	296.1	285.45	303.0	303.2	1.00
125	Nap1l1	0.64	0.030	186.05	178.95	197.95	201.15	216.85	184.85	203.35	186.5	316.45	363.5	363.8	385.05	194.3	303.1	1.56
126	Ncor1	0.34	0.023	101.8	87	98.1	94.5	97.4	87.4	94.1	89.3	126.8	132.3	132.9	141.9	94.4	119.6	1.27

	Gene Names	log2(FC)	P value	WT 1	WT 2	WT 3	WT 4	WT 5	WT 6	HOM 1	HOM 2	HOM 3	HOM 4	HOM 5	HOM 6	WT Avg	HOM Avg	Fold change
**127**	Ndufa12	0.31	0.007	302.8	297.55	301.55	305	294.1	289.95	292.05	316.5	404.8	377.1	413.75	419.8	298.5	370.7	1.24
**128**	Ndufa9	0.08	0.080	162	167.65	166	173.8	174.3	153.05	154.85	167.3	182.3	176.35	189.3	180.65	166.13	175.13	1.05
**129**	Ndufab1	0.06	0.036	291.5	296.8	306.8	286.6	288.8	327	299.1	295.7	300.8	321.4	335.4	322.1	299.58	312.42	1.04
**130**	Ndufs5	0.12	0.010	159.1	160	143.2	155	158	169.9	180.2	170.8	172.2	167.7	173.6	162.4	157.5	171.2	1.09
**131**	Nebl	0.05	0.003	376.9	376.4	364.1	349.35	370.05	389.05	329.05	335.5	412.45	425.25	412.9	384.65	371.0	383.3	1.03
**132**	Nedd4	0.19	0.031	152.7	142.3	140.3	148.15	164.1	161.1	153.4	161.5	161.65	191.45	181.35	184.9	151.4	172.4	1.14
**133**	Nes	0.24	0.000	372.9	347	365.3	363.8	368.5	378.3	434	425	407.9	445.7	422.8	463.4	366.0	433.1	1.18
**134**	Nexn	0.27	0.014	141.1	144.6	149.4	144.8	147.2	141.9	140.1	146.9	189.4	193	185.1	196.4	144.8	175.2	1.21
**135**	Nmd3	0.59	0.006	149.2	118.7	155.7	130.6	153.1	152.2	154.8	149.8	243.9	260.5	244.1	244.1	143.3	216.2	1.51
**136**	Nme1	0.03	0.026	294.9	260.85	269.95	271.4	284.75	242.7	273.5	257.3	267.7	280.35	278.85	299.1	270.8	276.1	1.02
**137**	Nono	0.15	0.020	157.8	160.6	144.6	155.2	167.65	161.8	165.55	156.65	169.45	177.75	195.5	188.2	157.9	175.5	1.11
**138**	Npm1	0.09	0.042	172.35	155.2	165.75	161.75	172.5	165.6	157.05	149.7	167.95	185.8	191.85	201.7	165.5	175.7	1.06
**139**	Oat	0.01	0.124	217.8	222.8	220.45	229.45	233.5	234.3	254.95	254.05	205	208.15	218.4	225.45	226.38	227.67	1.01
**140**	Obscn	0.08	0.028	314	333.3	327	285.7	324.3	327.6	338.8	337.8	335.1	338.9	342.6	330.6	318.7	337.3	1.06
**141**	Ogdh	0.38	0.040	256	257.4	254	241.1	267.2	238.7	245.5	254.2	365.1	355.9	383.4	365.8	252.40	328.32	1.30
**142**	Opa1	0.33	0.014	226.05	199.5	205.7	210.3	229.55	193.65	217.55	214.45	288.05	269.65	295.5	303.55	210.8	264.8	1.26
**143**	Otub1	0.26	0.009	85.2	100	84.5	96.8	90.9	96.1	100.7	98.2	123.3	104	126.6	108.6	92.3	110.2	1.19
**144**	P4hb	0.04	0.011	174.6	180.3	176.8	201	182.4	205.3	230.3	225.7	165.1	175.6	171.1	183.1	186.7	191.8	1.03
**145**	Pabpc4	0.03	0.053	479.8	512.9	483.4	517.7	479.4	544.7	488.2	528.6	506.1	521.4	512	525.3	502.98	513.60	1.02
**146**	Pafah1b1	0.36	0.004	240.15	237.25	230.7	256.55	263.85	245.65	278.8	262.6	311.85	328.45	352.95	355.65	245.7	315.1	1.28
**147**	Paics	0.11	0.043	241.45	242.35	258.6	247.25	271.95	244.1	253.2	251.85	260.3	288.45	275.6	290.85	251.0	270.0	1.08
**148**	Pde4dip	1.10	0.010	125.8	100.7	126.1	119.9	110.4	121.5	132.5	119.9	261.2	302.2	362.5	329.5	117.4	251.3	2.14
**149**	Pdia3	0.53	0.006	119.2	111.6	112.4	130.1	143.5	141	143.2	125.4	199.7	190	208.7	223.7	126.3	181.8	1.44
**150**	Pdia6	0.29	0.002	167.8	167	179.5	179.4	182.65	176.85	204.35	191.55	203	216.05	236.3	238.9	175.5	215.0	1.22
**151**	Pdlim1	0.10	0.011	330.9	333.95	330.25	346.1	356.95	343.4	360.8	348.05	360	374.3	366.8	381	340.3	365.2	1.07
**152**	Pdlim3	0.25	0.001	271.7	244.2	262.1	284.2	289.7	282.1	326.8	308.2	293.4	323.3	344.2	349.4	272.3	324.2	1.19
**153**	Pebp1	0.19	0.020	347.8	327.95	345.75	364.3	385.6	354.05	382	380	403.3	423.2	420.75	418.95	354.2	404.7	1.14
**154**	Pfkp	0.02	0.084	142	155.5	133.8	131.15	150.8	136.75	149	149	137.35	146	142.9	138.2	141.67	143.74	1.01
**155**	Pgam2	0.04	0.036	272	247	251.9	257.2	249.2	276.4	267.9	256.2	262.2	273	276.1	260.2	259.0	265.9	1.03
**156**	Pgd	0.21	0.036	233.7	251.05	244.85	243.95	253.65	256.05	270.7	249.75	280.85	298.9	307.25	307.5	247.2	285.8	1.16
**157**	Pgk1	0.11	0.028	251.2	243.2	244.7	247.8	237	270.1	266.4	276.3	272.1	278	250.6	271.5	249.0	269.2	1.08
**158**	Pgls	0.15	0.008	168.8	187.2	182.7	194.45	183.2	183.6	174.75	182.05	211.6	205.9	218.1	224.1	183.3	202.8	1.11
**159**	Pgm1	0.13	0.039	202	206.8	230.8	218.7	234.1	251.5	251.7	225.8	248.1	250.9	245.6	248.4	224.0	245.1	1.09
**160**	Phpt1	0.24	0.002	224	241	223.8	239.9	239.9	243.5	245.2	268.4	265.9	286.2	281.7	315.3	235.4	277.1	1.18
**161**	Pkm	0.07	0.029	240	218.3	230.75	228.8	244.55	233.15	248.75	268.5	227.6	239.35	227.5	252	232.6	244.0	1.05
**162**	Pon3	0.19	0.011	145.6	118.7	116.3	132.2	125.1	114.9	135.6	134.4	141.8	145.7	147.4	152.9	125.5	143.0	1.14
**163**	Popdc2	0.19	0.004	335.2	322.9	337.6	308	318.8	289	343.8	340.9	365.4	359.4	403.2	362.1	318.6	362.5	1.14
**164**	Postn	0.06	0.019	224.4	224.75	227.05	226.45	204.15	214.25	253.45	254.7	236.6	208.9	216.25	211.7	220.2	230.3	1.05
**165**	Ppa1	0.25	0.003	54.5	48.7	54.1	56.7	56.8	52.3	56.2	66.3	62.2	73.7	64.8	62.1	53.9	64.2	1.19
**166**	Ppib	0.16	0.010	234.45	233.8	230.35	229.9	239.9	225.1	250.1	239.65	253.3	265.35	258.85	289.6	232.3	259.5	1.12
**167**	Ppic	0.15	0.013	146.75	157.55	153.55	178.05	139.45	161.95	160.85	172.05	169.3	173.9	185.8	176.95	156.2	173.1	1.11
**168**	Ppp1cb	0.32	0.012	175.3	185.9	174.2	180.5	193	173.5	197.4	195.2	225.7	255.3	233.8	244.1	180.4	225.3	1.25

	Gene Names	log2(FC)	P value	WT 1	WT 2	WT 3	WT 4	WT 5	WT 6	HOM 1	HOM 2	HOM 3	HOM 4	HOM 5	HOM 6	WT Avg	HOM Avg	Fold change
169	Ppp2r1a	0.37	0.022	142.5	142.8	159.4	150.9	175.9	137.4	156.3	146.9	214.2	214.4	228.2	215.7	151.5	196.0	1.29
170	Prkcsh	0.17	0.009	169.2	179.4	182.6	179.3	170.5	160.7	183.3	187.8	182	194.8	215	212.9	173.6	196.0	1.13
171	Psma7	0.36	0.023	84.2	74.8	82	79.5	78.2	71.1	81.1	72.6	99.9	119.3	112.2	116.4	78.3	100.3	1.28
172	Psmc2	0.06	0.049	178.1	196.85	187.2	190.2	189.8	178.65	193.05	200.6	200.85	187.8	200.45	189.55	186.80	195.38	1.05
173	Psmc3	0.08	0.087	154.1	160.1	149.2	148.4	147.3	172.1	163.05	161.1	178.7	172.95	166.65	144.7	155.20	164.53	1.06
174	Psmc5	0.07	0.037	143.9	135.2	156.3	140.5	147.9	142.3	150.4	138.2	146.6	156.2	149.6	168.9	144.35	151.65	1.05
175	Psmd12	0.14	0.080	134.5	135.2	114.9	145.1	139.7	135.2	144.8	144.9	141.1	147.5	152.1	155.6	134.10	147.67	1.10
176	Psmd6	0.32	0.011	234.9	245	221.4	234	273.7	264	266.4	250.1	319.5	305.1	344.6	350.5	245.5	306.0	1.25
177	Psmd7	0.36	0.012	130.1	121.25	115.8	136.15	148.1	139.55	145.25	137	177.85	184.1	195.35	173	131.8	168.8	1.28
178	Psme1	0.04	0.021	100.4	98.6	95.2	109.5	98.4	101.2	91.7	87.5	110.3	111.8	104.9	113.3	100.6	103.3	1.03
179	Ptbp1	0.09	0.028	303.9	297.5	293.1	332.9	323	271.9	327.3	316.5	285.6	320.6	343.2	350.2	303.7	323.9	1.07
180	Ptn	0.20	0.029	73.6	78.6	74.8	90.5	83	91.7	93	104.1	88.5	83.1	91.2	103.8	82.0	94.0	1.15
181	Rab14	0.16	0.040	166.55	154.2	170.75	175.5	172	175.45	178.75	167.1	190.45	198.4	201.6	199.9	169.1	189.4	1.12
182	Rab5a	0.46	0.003	116.5	103.5	112.9	98.2	104.3	103	133.5	111	147.2	141	173.6	174.3	106.4	146.8	1.38
183	Rab5b	0.14	0.033	167.9	178.3	157.7	165.6	146.7	163.9	175.8	165.2	173.2	176.8	188.2	201.7	163.4	180.2	1.10
184	Ralbp1	0.22	0.049	62.6	50.7	59	60.3	68	73.1	67.4	59	73.6	76.2	79.5	78.6	62.3	72.4	1.16
185	Rbp1	0.21	0.007	105	97.6	110.7	111.2	120.9	114.2	131.9	121.1	144.6	120.9	122.2	122.6	109.9	127.2	1.16
186	Rcn2	0.17	0.012	244	230	224.3	252.1	230.1	260.1	277.9	246.8	257.8	260.1	275.4	298.9	240.1	269.5	1.12
187	Rcn3	0.14	0.016	338.5	364.3	324.7	378	345.2	351.6	395.9	367.8	388	376.6	393.7	399.9	350.4	387.0	1.10
188	Rpl10a	0.16	0.017	205.5	190.7	190.7	212.1	209.6	206.4	227.2	213.3	217.7	235.4	223.4	237	202.5	225.7	1.11
189	Rpl11	0.16	0.003	299.4	314.2	299.9	295.8	325	322.2	334	338.8	330	354.6	344	373.2	309.4	345.8	1.12
190	Rpl18	0.14	0.072	255.2	227.75	241.8	247.95	247.35	231.85	262.35	247.7	265.85	271.8	272.65	281.3	241.98	266.94	1.10
191	Rpl18a	0.13	0.021	162.9	150.1	140.4	137.2	163	145.7	146.6	142.1	151.2	175	166.2	200.6	149.9	163.6	1.09
192	Rpl19	0.12	0.041	103.2	102.6	91.2	102.6	105.15	104.8	106.9	106.75	116.8	108.6	113.65	110.05	101.6	110.5	1.09
193	Rpl21	0.18	0.018	214.3	223.2	196.8	217.8	234.3	239.6	243.8	221.4	247	241.7	268.4	276.4	221.0	249.8	1.13
194	Rpl23	0.12	0.047	79.9	68.8	71.2	64.8	77.2	70.8	70.5	62.2	85	76.2	95.4	80.3	72.1	78.3	1.09
195	Rpl26	0.19	0.021	160.9	157.3	159.6	155.1	182.4	155.4	190.8	152.9	183.4	185.7	207.6	188.8	161.8	184.9	1.14
196	Rpl27	0.24	0.022	76.35	66.4	66.6	70.05	72	76.7	74.6	74.1	94.95	83.45	86.4	93.75	71.4	84.5	1.18
197	Rpl28	0.15	0.038	169.35	158.6	152.7	162.55	168.75	173	163.95	169.95	187.15	190.9	188.65	190.8	164.2	181.9	1.11
198	Rpl3	0.19	0.038	146.5	143.45	147.15	147.15	136.55	152.35	162.3	143.7	163.95	170.95	173	181.05	145.5	165.8	1.14
199	Rpl31	1.23	0.020	81.95	87.7	89.5	91.95	95.7	99.85	94.05	92.7	268.5	261.65	281.2	285.55	91.1	213.9	2.35
200	Rpl32	0.33	0.004	240.85	221.4	214.6	230.85	246.8	235.7	279.9	259.25	318.25	283.4	310.35	301.15	231.7	292.1	1.26
201	Rpl36a-ps1	0.22	0.025	130.7	134.7	124.1	116.4	125.7	128	149.3	138.7	147.6	140.5	161.4	145.9	126.6	147.2	1.16
202	Rpl37	0.23	0.038	149.1	122.3	127.1	141.3	128.1	135.9	138.7	134.1	189.7	165.1	168.3	145.6	134.0	156.9	1.17
203	Rpl4	0.06	0.026	230.9	222	224.9	234.9	223.6	239.6	238.2	240.7	230.8	246.2	240.2	234.4	229.3	238.4	1.04
204	Rpl5	0.09	0.012	319	291.75	312.4	289.65	336.3	298.45	326.25	306.55	311.7	328.7	343.6	348.4	307.9	327.5	1.06
205	Rpl8	0.18	0.022	206	220.9	226.8	215.5	223.8	234.2	233.1	221.3	263.7	252.6	269.1	267.9	221.2	251.3	1.14
206	Rpl9	0.21	0.020	276.3	283.9	275.3	312.6	305	297	323.2	314.3	302.9	342.4	349.3	389	291.7	336.9	1.15
207	Rplp2	0.17	0.011	152.4	155.2	158.5	158.3	166.3	154.5	179.6	158.5	163.7	179.4	170.3	210.2	157.5	177.0	1.12
208	Rps11	0.03	0.034	180.25	183.05	177.1	182.95	188.2	189.9	196.6	196.3	168.15	177.05	188.95	199.1	183.6	187.7	1.02
209	Rps13	0.28	0.048	145.9	126.9	135.2	132.5	166.8	117	168	140.5	168.5	157.6	177.9	190.1	137.4	167.1	1.22
210	Rps17	0.04	0.006	244.55	240.75	236.2	243.3	230.25	251.3	250.85	252.75	230.15	248.55	252.85	246.95	241.1	247.0	1.02

	Gene Names	log2(FC)	P value	WT 1	WT 2	WT 3	WT 4	WT 5	WT 6	HOM 1	HOM 2	HOM 3	HOM 4	HOM 5	HOM 6	WT Avg	HOM Avg	Fold change
211	Rps19	0.05	0.048	228.95	202.7	206.95	221.2	252.7	211.9	238.95	207.65	224.05	233.6	226.25	245.2	220.7	229.3	1.04
212	Rps2	0.34	0.039	155.2	142.05	157.55	149.4	196.55	154.2	175.2	149.6	205.3	220.45	210.6	243.55	159.2	200.8	1.26
213	Rps20	0.48	0.047	257.85	280.65	262.35	301.6	255.6	280.05	275.45	290.8	382.4	433.05	426.35	469.7	273.0	379.6	1.39
214	Rps23	0.19	0.024	83.45	78.4	81.6	76.6	86	79.4	75.8	80.05	94.8	97.55	97.45	107.8	80.9	92.2	1.14
215	Rps25	0.84	0.030	137.7	112.4	121.4	115.8	161.9	110.9	145	131	243.5	304	245.9	290.1	126.7	226.6	1.79
216	Rps3	0.19	0.038	161.1	144.05	163.55	165.45	186.6	151.5	180.05	168.05	186.1	187.45	184.45	205.45	162.0	185.3	1.14
217	Rps3a	0.19	0.019	143.1	142.8	120.9	153.2	164.2	161.4	150.4	162.7	166.6	167.5	184.8	175.3	147.6	167.9	1.14
218	Rps4x	0.22	0.032	128.25	120.85	130.05	133.65	138.45	121.65	131.6	130.45	157.6	154.9	155.15	168.7	128.8	149.7	1.16
219	Rps9	0.34	0.018	111.1	110.45	92.5	107.5	116.3	105.45	114.2	121.45	143.35	137.9	139.9	154.75	107.2	135.3	1.26
220	Rrbp1	0.34	0.045	145.9	136.9	141.7	181.1	152.8	214.2	191.5	177.3	250.8	208.1	243	162.4	162.1	205.5	1.27
221	Rtn4	0.09	0.002	148.7	174.6	149	152.55	156	171.05	176.4	173.05	168	157.6	162.05	175	158.7	168.7	1.06
222	S100a11	0.15	0.048	553.3	562.25	606.8	604.45	618.7	527.85	672.9	650.2	535.55	674.65	656.55	656.2	578.9	641.0	1.11
223	Sdha	0.13	0.042	179.15	196.05	182.85	181.7	182.2	175.1	168.55	176.9	203.25	206.25	227	222.65	182.84	200.77	1.10
224	Sdhb	0.73	0.015	102.5	95.4	114.9	109.1	119.7	102.7	104.8	111.9	219.8	211.3	202.5	221.4	107.4	178.6	1.66
225	Septin7	0.14	0.009	239.6	246.1	238	239.7	254.95	267.95	259.05	264.15	272.4	274.7	286.3	286.05	247.7	273.8	1.11
226	Serbp1	0.49	0.041	110	97.4	93.05	110.25	121.9	102.55	112.5	111.5	153.1	165.35	168.85	178.9	105.9	148.4	1.40
227	Serpina3g	0.97	0.014	90.3	79.2	82.6	86.2	102.9	83.8	94.7	79.8	210.6	218.7	243	182.2	87.5	171.5	1.96
228	Serpinb6a	0.16	0.063	137.3	160.1	152	167.1	151.5	140.2	159.4	144.1	162.2	166.2	175	205.5	151.37	168.73	1.11
229	Serpinh1	0.02	0.016	135.4	138.5	146.9	147.6	125.9	143.1	157.6	154.4	125.9	126.7	137.8	145.8	139.6	141.4	1.01
230	Skp1	0.19	0.002	214.9	207.3	230.8	225.6	242.4	219.4	243	238.9	248	264.2	276.8	258.8	223.4	255.0	1.14
231	Slc3a2	0.23	0.000	394.2	387.3	369.7	395.4	416.1	421	458.1	439.7	464.3	467.1	494.8	471.1	397.3	465.9	1.17
232	Smc4	0.16	0.034	63.9	60.3	68.9	70.4	65.5	62.8	66	68.6	78.3	67.1	81.9	75.4	65.3	72.9	1.12
233	Smpx	0.29	0.015	236.2	275.5	212	243.4	223	242.5	315.3	338.3	314.7	227.4	271.2	289.1	238.8	292.7	1.23
234	Snd1	0.10	0.023	455.75	467.65	468.6	495.65	473.85	477.6	502.05	508.75	467.95	504.8	508.6	542.45	473.2	505.8	1.07
235	Snrpb	0.01	0.038	235.8	239.2	234.35	246.35	253.95	251.4	247.2	242.45	211.5	254.4	261	256.75	243.5	245.6	1.01
236	Sod1	0.18	0.007	119.8	135	113.8	133.3	132	150.2	144.6	134.2	154.1	156.8	149.7	151.9	130.7	148.6	1.14
237	Sorbs2	0.13	0.001	213.6	220.9	201.9	198.4	219.6	221.5	247.6	256.6	239	238.6	201.4	216.9	212.7	233.4	1.10
238	Sptan1	0.12	0.035	193.3	209.5	196.2	200.95	214.45	216.8	211.15	216.3	223.1	230.85	232.4	224.75	205.2	223.1	1.09
239	Srl	0.08	0.015	227.85	203.85	217.45	219.85	236.2	218.75	248.75	234.55	257.85	226.1	214.5	218.8	220.7	233.4	1.06
240	Srsf1	0.16	0.031	322.95	340.4	328.85	359.95	344.05	365.1	377	364.9	371.95	377.25	402.65	405.25	343.6	383.2	1.12
241	Ssr1	0.26	0.020	111.9	124.6	118.1	128.9	148	125.6	137.6	131.4	141.1	161.4	153.3	180.7	126.2	150.9	1.20
242	Suclg2	0.77	0.008	112.2	98.2	118.5	100.8	124.2	106.5	128	105	216.5	213.3	225.1	238.7	110.1	187.8	1.71
243	Syncrip	0.21	0.007	145.6	126.5	140.8	142.9	144.1	139.4	150.5	152.1	164.2	151.3	164.7	187.3	139.9	161.7	1.16
244	Tagln	0.30	0.024	88.7	103.25	91.65	109.9	96.3	100.25	116.65	112.45	104.3	117.85	121	154.95	98.3	121.2	1.23
245	Tbca	0.32	0.001	165.25	152.6	168.15	153.3	168	171.3	200.65	176.55	195.8	212.25	216.95	216.3	163.1	203.1	1.25
246	Tcirg1	0.24	0.007	290.7	292	297	339	298.2	304.3	340.2	326.9	387.4	317.9	401.4	370	303.5	357.3	1.18
247	Tmed2	0.95	0.009	209.8	218.05	213.15	223.85	235.4	222.05	236.25	229.8	464.65	506.8	555.4	567.7	220.4	426.8	1.94
248	Tmed9	0.21	0.031	112.3	130.9	119.05	115.15	118	112.25	126.8	120.35	139	136.4	143.05	154.95	117.9	136.8	1.16
249	Tmsb4x	0.16	0.042	181.1	200.9	200.3	195.8	194.6	193.6	203.7	208.9	192.5	205.7	231.6	257.6	194.4	216.7	1.11
250	Tnnt2	0.18	0.039	150.9	151.2	163.8	150.7	160.6	158.2	151.4	141.3	197.2	190	185.5	194.4	155.90	176.63	1.13
251	Tpp2	0.15	0.002	418.4	393.6	386.3	405.1	433.2	409.8	454.5	413.2	471.9	448.7	454.8	468.8	407.7	452.0	1.11
252	Tuba1a	0.07	0.027	181.1	172.7	170.3	183.5	194.9	160.7	209.9	190.3	174.4	179.3	184.5	178.3	177.2	186.1	1.05

	Gene Names	log2(FC)	P value	WT 1	WT 2	WT 3	WT 4	WT 5	WT 6	HOM 1	HOM 2	HOM 3	HOM 4	HOM 5	HOM 6	WT Avg	HOM Avg	Fold change
**253**	Txnl1	0.16	0.009	239.8	256.4	237.9	251.3	269.3	262.3	263.1	263.4	274.6	285.5	306.2	301.3	252.8	282.4	1.12
**254**	Uba1	0.13	0.040	197.1	197.1	207.3	197.5	205.4	195.4	200.9	212.5	215	227.7	241.4	216.6	200.0	219.0	1.10
**255**	Ube2l3	0.07	0.022	119.2	91	121.3	113.8	122.2	122.9	115.3	119.3	122.4	125.7	124.5	119.7	115.1	121.2	1.05
**256**	Uchl1	0.09	0.006	164.6	148.7	175.7	165.9	172.7	156.5	182	170.7	175.6	174	164.5	179.3	164.0	174.4	1.06
**257**	Unc45b	0.13	0.032	182.4	183.1	184.3	183.8	213.7	184.3	179.2	179.3	188.5	224.8	245.6	223	188.6	206.7	1.10
**258**	Uqcrc2	0.03	0.068	138	119.8	127.7	121.5	134.3	131.2	124.6	118.9	133.8	134.6	147.3	131.4	128.75	131.77	1.02
**259**	Vcan	0.32	0.005	366.1	385.5	354.4	381	416.8	435.7	424	443.6	434.7	533.4	539	546.2	389.9	486.8	1.25
**260**	Vezf1	0.25	0.022	27.1	26.6	23.7	27.7	28.4	29.6	30.6	31.2	35.4	32.9	37.7	25.8	27.2	32.3	1.19
**261**	Vps29	0.07	0.010	127.1	140.05	140.2	139.8	147.25	144.65	136.6	144.95	155.6	143.05	151.95	147.55	139.8	146.6	1.05
**262**	Ywhab	0.05	0.036	231.1	232.85	221.1	234.7	238.95	229.6	230.35	249.7	219.85	234.25	244.95	261.05	231.4	240.0	1.04
**263**	Zc3h4	0.65	0.011	14.2	14.5	14.5	30.9	19	23	26.7	25.5	36.6	25.7	37.2	30	19.4	30.3	1.57
**264**	Zfp780b	0.47	0.026	217.9	209.7	187.9	213.6	172	195.4	203	188.8	296	270.4	361.9	340.2	199.4	276.7	1.39
**265**	Zyx	0.36	0.020	51.7	51.2	47.6	47.7	49.8	50.7	48.4	48.7	70.8	75.7	73.5	65.8	49.8	63.8	1.28
