# Supplementary material for: Insights into the Role of a Cardiomyopathy-Causing Genetic Variant in ACTN2
Source: Cells. 2023 Feb 24;12(5):721. doi: 10.3390/cells12050721 (PMC10001372; doi:10.3390/cells12050721)
Supplement: Supplementary file 1 [file cells-12-00721-s001.zip › Table S7.pdf]

	Gene Names	log2(FC)	P value	WT 1	WT 2	WT 3	WT 4	WT 5	WT 6	HOM 1	HOM 2	HOM 3	HOM 4	HOM 5	HOM 6	WT Avg	HOM Avg	Fold change
1	Acaa2	-0.296	0.031	194.3	200.6	174.2	197	176.3	169.9	178.3	159.7	141.1	143.4	142.4	141	185.38	150.98	0.81
2	Acadm	-0.304	0.201	254.6	244.65	261.85	223.35	220	227.9	236.85	205.7	196.6	186.65	167.35	167.4	238.73	193.43	0.81
3	Acadsb	-0.241	0.076	38.3	37.5	38	35.8	37.9	33.9	35	37.4	29.5	35.4	23.5	26.6	36.90	31.23	0.85
4	Acadvl	-0.423	0.021	231.4	233.6	246.9	198.3	210.5	197	198.2	185.8	151.2	156.7	157.2	134	219.62	163.85	0.75
5	Acot13	-0.429	0.013	460	507.8	481.8	446.4	423.9	491.3	418.2	418.1	312	318.3	309.8	311.3	468.53	347.95	0.74
6	Actn2	-0.440	0.008	264	282.85	266.15	261.8	281.5	267.2	171.85	181.55	251.3	208.5	214.6	169.15	270.58	199.49	0.74
7	Actr3	-0.572	0.047	267.45	264.2	271.95	296.45	280.5	271.65	288.15	292.75	138.7	142.65	126.85	122.45	275.37	185.26	0.67
8	Adprhl1	-0.478	0.065	273.7	336.3	346.5	307.7	277.7	335.6	321.1	333.7	169.2	183.5	170.9	169.5	312.92	224.65	0.72
9	Adsl	-0.342	0.037	76.55	83.95	79.15	79.9	74.45	77.55	77.15	71.4	51.4	58.3	59.4	54.5	78.59	62.03	0.79
10	Aimp1	-0.431	0.041	282.9	327.9	284.6	293.2	282.6	309.9	280.5	312.3	176.4	192.8	174.2	185	296.85	220.20	0.74
11	Ak2	-0.305	0.027	457.6	443.2	494.3	481.8	466.1	423	428.2	451.4	316.5	361.2	353.5	327.5	461.00	373.05	0.81
12	Aldh1b1	-0.251	0.007	132.1	127.6	123.8	139	127.5	135.4	113.7	119.9	109.8	100.4	104.3	111.9	130.90	110.00	0.84
13	Anp32e	-0.414	0.037	283.75	283.7	277.5	268.3	254.55	255.85	243.2	247	193.15	181	169.5	184.8	270.61	203.11	0.75
14	Apobec2	-0.047	0.077	318.2	302.3	322.2	302.3	328.8	303.7	289.7	295.1	286.2	317.8	309.8	318.7	312.92	302.88	0.97
15	Aprt	-0.153	0.097	422.75	423.55	448.2	416.05	432.95	421.95	444	434.9	337.95	365.25	363.85	362	427.58	384.66	0.90
16	Atp2a2	-0.101	0.046	281.9	314.6	292.8	313.6	303.7	304.1	268.4	266.3	277.5	281.6	302.9	291.8	301.78	281.42	0.93
17	Atp5a1	-0.139	0.039	226.5	238.7	255.9	219.2	232	228.8	259.3	232.1	189.8	205.4	182.1	204	233.5	212.1	0.91
18	Atp5c1	-0.049	0.085	165.2	148.4	160.4	165.2	166.9	142.3	141.5	139.7	159.1	162.4	151.1	162.8	158.07	152.77	0.97
19	Atp5f1b	-0.161	0.065	288.25	298.4	291.3	285.5	296.85	265.75	261.65	300.6	247.3	242.15	254.95	236.8	287.68	257.24	0.89
20	Atp5f1e	-0.159	0.131	164.05	173.9	173.9	150.7	154.95	136.8	137.35	135.6	142	154.55	146.95	138.1	159.05	142.43	0.90
21	Atp5mg	-0.234	0.041	152	148.1	159.8	144.7	160.4	130.7	144.3	146.3	109.6	114.1	119.5	128	149.28	126.97	0.85
22	Atp5mk	-0.161	0.062	221.4	185.4	203.5	178.5	204.7	186	197.5	175	164.9	168.2	171.7	177.3	196.58	175.77	0.89
23	Atp5pb	-0.354	0.022	213.9	238.5	232.6	208	198.5	225.8	193.2	189.6	159	153.5	152.2	183	219.55	171.75	0.78
24	Atp5pd	-0.230	0.035	147.6	129.9	126.5	134.8	119.2	120.4	111.3	107.9	115	104.6	115.7	109.2	129.73	110.62	0.85
25	Blmh	-0.027	0.006	182.1	176.95	180.05	178.35	181.2	192.3	198.95	188.5	158.95	170.8	177.4	176.05	181.8	178.4	0.98
26	Blvrb	-0.038	0.083	249.65	237.55	252.25	256.95	249.55	258.8	281.5	257.4	251.45	217.95	224.6	233	250.79	244.32	0.97
27	Btf3	-0.194	0.045	330.8	370.3	373.8	328.3	322.7	346.9	340.9	338.4	277.3	283.5	294.2	277.6	345.47	301.98	0.87
28	Bzw1	-0.288	0.051	94.4	110.9	97.5	90.8	101.9	110.5	102.7	90.6	63	76.4	88.6	74.9	101.00	82.70	0.82
29	C1qbp	-0.112	0.039	279.75	300.7	304.1	274.15	277.35	300.8	281.25	297.15	241.35	260.4	264.35	263	289.48	267.92	0.93
30	Cacybp	-0.293	0.050	596.3	629.75	590.85	611.25	556.4	639.7	579.9	608.35	442.9	445.9	428	453.9	604.04	493.16	0.82
31	Calu	-0.087	0.031	273.5	316.6	255.3	297.7	278.2	310	302.4	334.7	233.9	239.4	255.3	264.5	288.6	271.7	0.94
32	Cand1	-0.104	0.017	237.5	261.8	282.4	250.6	233	257.5	236.9	249.9	227.3	213.3	246.4	242.6	253.8	236.1	0.93
33	Cand2	-0.598	0.005	268.2	280	292	256.2	261	246.5	228.4	247.5	141.8	154.1	156.4	131.4	267.3	176.6	0.66
34	Cct2	-0.159	0.048	281.2	283.6	286.2	298.5	281.6	316.65	304.55	302.8	240	236.15	246.75	234.8	291.29	260.84	0.90
35	Cdk1	-0.366	0.010	261.5	255.2	268.9	263.4	285.9	262	241.4	225.5	175.2	211.6	208.1	177.4	266.15	206.53	0.78

	Gene Names	log2(FC)	P value	WT 1	WT 2	WT 3	WT 4	WT 5	WT 6	HOM 1	HOM 2	HOM 3	HOM 4	HOM 5	HOM 6	WT Avg	HOM Avg	Fold change
36	Cfl1	-0.086	0.030	371.55	389	380.2	406.6	346.2	425.7	347.2	394.85	344.35	369.25	348	380.8	386.5	364.1	0.94
37	Ckb	-0.146	0.057	279.95	288.95	278.75	276.05	262.2	252.55	252.9	259.95	257.8	240.45	231.3	238.85	273.08	246.88	0.90
38	Cltb	-0.383	0.028	20.6	18.3	21.9	19.7	23.2	25.9	19.8	17.3	18.2	14	14.8	15.3	21.60	16.57	0.77
39	Cluh	-0.284	0.088	225.3	256.05	243.1	243.85	227.9	252.85	234.9	271.6	171.15	172.8	166.15	173.35	241.51	198.33	0.82
40	Copg2	-0.086	0.031	241.1	240.75	218.35	227.2	222.75	228.35	227.9	241.35	194.6	200.4	217.7	216.95	229.75	216.48	0.94
41	Cops2	-0.474	0.033	239.3	285.1	284.5	246.9	244.2	249	246.5	254.1	149.3	157.6	156.3	151.2	258.17	185.83	0.72
42	Cops7b	-0.221	0.052	145.1	157	154	156.2	152.8	143.5	154.4	150.4	114.3	129.5	111.8	119.1	151.43	129.92	0.86
43	Cotl1	-0.093	0.112	121.85	105.8	121.2	116.3	114.1	110.4	124.95	102.15	97.85	105	102.45	114.1	114.94	107.75	0.94
44	Cox20	-0.524	0.034	272.5	252.4	274	249.2	246.8	266.7	242	270.5	147.3	147.2	137.8	141.3	260.27	181.02	0.70
45	Cox5a	-0.601	0.063	357.7	371.4	345.9	313.3	302.7	340	313.2	279.6	204.1	169.3	190.7	182.1	338.50	223.17	0.66
46	Cox6a1	-0.428	0.027	199.1	201.2	223.6	247.1	177.6	209.9	189.1	196.8	147.3	148.2	132.3	121.5	209.75	155.87	0.74
47	Cox8b	-0.573	0.027	103.3	113.7	114.4	95.7	93.1	95.5	92.6	98.6	58.8	58.4	54.3	51.3	102.62	69.00	0.67
48	Cse1l	-0.198	0.029	340.6	328.05	366.3	329.5	348.7	328.5	318.65	317.5	264.5	297.45	286.7	295.45	340.28	296.71	0.87
49	Ctps1	-0.109	0.029	286.7	276.3	267.8	276.3	257.5	264.1	258.9	247.3	259.2	235.1	256.1	254	271.45	251.77	0.93
50	Cyb5r3	-0.140	0.038	208	235.1	198.2	197.9	189.3	281.3	210.4	232.6	193.6	204.6	177	170.6	218.30	198.13	0.91
51	Ddc	-0.465	0.030	236.9	269.1	253.7	253.9	210.8	268.7	211.1	255.3	166.2	153.3	144	151.8	248.85	180.28	0.72
52	Dlat	-0.180	0.332	127.35	129.45	140.15	121.65	123.5	111.45	112.3	119.15	107.4	101.9	114.95	109.5	125.59	110.87	0.88
53	Dld	-0.281	0.045	263.55	275.6	273.45	257.15	263	258.6	253.05	260.2	191.75	203.9	201.25	199.85	265.23	218.33	0.82
54	Dnaja2	-0.126	0.296	272.25	284.85	300.85	270.65	266.15	274.5	275.4	272.6	217.15	254	255.1	255.05	278.21	254.88	0.92
55	Dnmt3a	-0.626	0.069	142.65	133.8	131.4	147.4	145.8	165.7	158.6	147.7	66.85	65.45	67.55	55.5	144.46	93.61	0.65
56	Dpp3	-0.254	0.067	74.3	84.45	87.9	79.65	71.55	86.5	83.65	77.3	62.2	64.4	60.3	58.45	80.73	67.72	0.84
57	Drg1	-0.259	0.038	67.7	73	78.2	76.5	69.2	73.7	74.8	67.7	57.2	58	50.7	57.8	73.05	61.03	0.84
58	Dync1h1	-0.172	0.090	173.9	176.55	190.35	183.75	168.1	183.05	184.1	188.15	133.55	151.25	154.65	143.15	179.28	159.14	0.89
59	Eef1a2	-0.063	0.024	256	232.6	244.6	225.1	254.6	223	244.5	246.8	218.5	220.8	222.6	221.7	239.3	229.2	0.96
60	Eif3b	-0.316	0.020	198.6	217.9	198.4	203	206.9	198.1	190.9	189.2	131.9	151.9	155.3	163.4	203.82	163.77	0.80
61	Eif3m	-0.246	0.047	67.5	75.1	86.5	86.9	71.6	75.5	73.8	73.7	63.3	58.9	59.5	61.3	77.18	65.08	0.84
62	Eif4g1	-0.275	0.037	151.4	163.3	171.4	163.3	156.5	161.1	163.9	155.9	121.4	122.9	119.2	116.1	161.17	133.23	0.83
63	Esd	-0.098	0.173	244.7	232.4	247.7	242.15	233.5	241.45	248.55	245.3	197.65	210.45	222	222.95	240.32	224.48	0.93
64	Etfa	-0.326	0.099	278.6	271.2	261.75	279.4	259.5	260.2	234.8	235.55	194.55	208.85	208.85	202.6	268.44	214.20	0.80
65	Etfb	-0.381	0.066	144.95	154.35	147.7	148.45	134.75	135.8	119.6	123.15	106.75	102	107	106.6	144.33	110.85	0.77
66	Fdps	-0.229	0.049	161.9	178	175.8	174.5	188.3	151.4	166.6	168.9	124.1	144.8	134.3	139.9	171.65	146.43	0.85
67	Fh1	-0.310	0.009	135.5	150.4	158.4	156.7	153.8	158.9	129.1	119.1	104.7	129.4	133.2	121.7	152.28	122.87	0.81
68	Galk1	-0.267	0.035	233.1	255.9	242.8	248.6	263.7	258.7	251.1	252.1	177.2	190.7	200.3	177.7	250.47	208.18	0.83
69	Gatad1	-0.292	0.018	235.2	205.5	206.9	205.7	186.7	183.5	180.6	167.1	158.4	154.1	161.3	177.6	203.92	166.52	0.82
70	Glrx3	-0.131	0.071	222.5	188.9	211	197.6	212.4	202	202.5	203.6	177.3	171.3	181.7	190.5	205.73	187.82	0.91

	Gene Names	log2(FC)	P value	WT 1	WT 2	WT 3	WT 4	WT 5	WT 6	HOM 1	HOM 2	HOM 3	HOM 4	HOM 5	HOM 6	WT Avg	HOM Avg	Fold change
**71**	Gnb2	-0.301	0.031	402.6	421.5	416.7	424.3	373.2	420.6	390	405.6	302.7	285	291.1	321.6	409.82	332.67	0.81
**72**	Gosr1	-0.302	0.087	73	77.6	73.4	81.3	76.6	73.7	86.6	73.2	48.3	57.9	51.2	52.3	75.93	61.58	0.81
**73**	Got1	-0.185	0.076	216.9	193.45	200	190.1	185.6	194	174.45	185.45	174.5	166.85	166.1	171	196.68	173.06	0.88
**74**	Gtf2i	-0.269	0.030	163.7	181.3	203.3	185.6	188.6	195.8	167.3	181.8	130.7	153.7	153.2	141.1	186.38	154.63	0.83
**75**	Gys1	-0.121	0.097	173.9	191.4	181.25	184.2	174.4	191.9	170.8	186.9	164.7	158.7	162.15	165.75	182.84	168.17	0.92
**76**	H2az2	-0.201	0.087	261.6	266.9	264.6	275.2	283.5	251.1	256.8	233.9	216.3	233.7	228.5	225.3	267.15	232.42	0.87
**77**	H4c1	-0.204	0.038	312.95	342.1	292.95	331.5	316.35	318.4	310.6	305.45	239.3	271.65	264.7	269.95	319.04	276.94	0.87
**78**	Hadh	-0.028	0.062	226.5	239.7	231.9	219.5	216	254.4	232	227.5	227.1	220.3	215.4	238.6	231.33	226.82	0.98
**79**	Hadha	-0.568	0.024	368.55	381.15	373.4	376.85	328.75	343.5	317.65	307.25	209.4	211.9	212.3	206.6	362.03	244.18	0.67
**80**	Hibadh	-0.404	0.021	132.7	147.3	133.9	131.4	130.7	119.9	116.8	127.3	92.2	93.2	90.1	82	132.65	100.27	0.76
**81**	Hmgb2	-0.207	0.048	168.4	189.3	181.3	192.7	166.8	177.1	173.8	193.2	138.3	136.5	133.1	156.7	179.27	155.27	0.87
**82**	Hmgcl	-0.389	0.037	245.1	257.5	277.6	249	255.2	259.1	252.8	258	155.8	180.8	174	157.2	257.25	196.43	0.76
**83**	Hnrnpl	-0.104	0.041	203.65	221.8	202.15	214.85	222.25	217.2	224.05	207.85	173.65	186.6	204.9	195.5	213.7	198.8	0.93
**84**	Idh2	-0.053	0.037	247.8	240.7	253.8	239.1	251	226.6	233.4	260.8	220.5	232.6	242.8	216.6	243.17	234.45	0.96
**85**	Idh3b	-0.249	0.057	203.6	214.35	219.7	214.65	221.2	193.3	195.85	194.35	169.35	166.65	170.75	168.7	211.13	177.61	0.84
**86**	Impdh1	-0.389	0.039	111.9	120.8	125	110.2	118.7	104.7	110.6	117.4	73.9	76.4	80	69.8	115.22	88.02	0.76
**87**	Impdh2	-0.318	0.030	85.7	93.1	97.8	90.9	80.8	74.3	79.6	78.3	59	70.6	71.8	59.9	87.10	69.87	0.80
**88**	Iqgap1	-0.395	0.038	165.9	185.4	198.6	181.9	168.8	169.8	179	173.5	116.1	106.9	117.8	120.9	178.40	135.70	0.76
**89**	Isoc1	-0.407	0.017	134.5	149.6	161.8	145	136.9	134.8	119.9	135.6	93	106.5	93.2	102.4	143.77	108.43	0.75
**90**	Itgb1bp2	-0.181	0.036	148.7	155.2	171.7	154.3	169.5	152.8	152.8	146.7	126.7	147.4	138.7	127.5	158.70	139.97	0.88
**91**	Kpnb1	-0.026	0.043	298.4	300.5	308.25	283.15	303.2	318.45	310.4	306.75	273.2	295.5	293.25	301.05	302.0	296.7	0.98
**92**	Lamb1	-0.170	0.005	440.1	422.1	415.7	436.1	442.5	410.4	444.3	427.1	366.7	347.4	369.7	327	427.8	380.4	0.89
**93**	Lap3	-0.259	0.061	212.8	226.7	212.4	216.6	193.2	229.3	225.9	212.6	161	157.3	158.3	163.4	215.17	179.75	0.84
**94**	Lars	-0.442	0.043	223	232.3	247.4	235.6	194.6	246.8	214	243.1	135.2	143.6	130.8	148.7	229.95	169.23	0.74
**95**	Ldhb	-0.241	0.008	260.7	236.8	257.4	237.55	234.3	239.3	245.55	253.65	194.9	185.05	178.85	182.7	244.3	206.8	0.85
**96**	Lrpprc	-0.386	0.051	217.6	261.5	238.5	242.8	230	280.4	243.2	248.8	146.2	151.7	176	159.4	245.13	187.55	0.77
**97**	Lsm6	-0.202	0.072	172	211.3	204.7	168.5	188.8	177.7	180.9	171.7	159	178.5	146.2	139.9	187.17	162.70	0.87
**98**	Maged2	-0.603	0.048	56.3	57.1	58.3	86.2	67.4	67.8	64.2	60	42.5	35.2	24.9	32.1	65.52	43.15	0.66
**99**	Mb	-0.082	0.017	139.15	112.7	131.45	127.85	133.75	102.95	103.35	101.3	133.95	122.6	129.7	115.4	124.6	117.7	0.94
**100**	Mdh2	-0.361	0.020	410.5	388.3	380.2	360.3	355.6	333.2	298.7	310.8	296.8	280.7	273.8	274.4	371.35	289.20	0.78
**101**	mKIAA0038	-0.192	0.081	270	293.5	292.65	285	287	301.4	290.6	290.9	228.8	231.4	238.55	233.5	288.26	252.29	0.88
**102**	Mrpl28	-0.493	0.032	233.6	251.3	256.8	215.2	216.7	252.4	221.4	236.6	137.5	150.5	131.5	135.4	237.67	168.82	0.71
**103**	Mtco2	-0.238	0.025	504.3	533.4	488.4	502.2	464.1	453	426.5	435.7	421.4	404	402.1	407.5	490.90	416.20	0.85
**104**	Mtnd5	-0.210	0.088	122.15	105.4	134.35	102	118	109.5	101.1	93.55	93.4	109.7	97	103.05	115.23	99.63	0.86
**105**	Mtx2	-0.318	0.026	469.8	509.6	541.3	502.7	465.4	536.6	453.9	499.1	354.7	379.2	368.4	371.4	504.23	404.45	0.80

	Gene Names	log2(FC)	P value	WT 1	WT 2	WT 3	WT 4	WT 5	WT 6	HOM 1	HOM 2	HOM 3	HOM 4	HOM 5	HOM 6	WT Avg	HOM Avg	Fold change
106	Myh9	-0.143	0.050	261.5	235.7	247.9	253.3	255.8	230.9	233.9	229.9	218.1	221.1	214.2	227.4	247.52	224.10	0.91
107	Myl3	-0.308	0.048	443.4	409.9	413.7	430.9	421.6	396.4	409.9	401.7	293.5	334.3	288.3	304	419.32	338.62	0.81
108	Myo18a	-0.330	0.040	218.7	205.4	235.9	209.1	187.7	212.8	213.3	203.1	136.3	154.9	151.3	150.9	211.60	168.30	0.80
109	Naca	-0.376	0.050	1083.2	1143.9	1083.4	1147.8	1083.9	1025.9	1072.6	1169.4	683.1	708.9	710.7	715.4	1094.68	843.35	0.77
110	Naga	-0.257	0.066	91.8	111.8	115.7	112	99	118.8	106.9	107.4	75.3	80.6	84.1	88.9	108.18	90.53	0.84
111	Nasp	-0.063	0.037	297.8	318.95	278.4	298.6	280.55	351.35	298.1	303.05	294.45	274.05	292.4	285.6	304.3	291.3	0.96
112	ND2	-0.393	0.018	229.8	255.1	247.7	233.8	221.2	226.2	216.9	216.7	161.9	173.7	151.3	156.3	235.63	179.47	0.76
113	ND4	-0.352	0.037	330.4	349.6	354.4	319.8	339.4	327.9	307.55	302.05	236.65	249.75	252.85	235.05	336.92	263.98	0.78
114	Ndrg2	-0.220	0.032	340.7	342.9	343.3	328.4	317	286.2	305.5	313.9	244.8	269.8	265.9	281.5	326.42	280.23	0.86
115	Ndufa10	-0.155	0.084	247.9	231.3	260.9	233.3	229.8	220	225	210.2	202.9	218.8	212.2	209.1	237.20	213.03	0.90
116	Ndufa13	-0.281	0.057	90.7	94.6	94.7	86.75	83.7	80.8	82.6	81.95	70.2	68.7	68.1	65.7	88.54	72.88	0.82
117	Ndufa4	-0.025	0.025	311.6	336.6	326.9	327.9	337.9	289.3	295.4	306.3	336.6	308.3	326.3	324	321.70	316.15	0.98
118	Ndufa5	-0.163	0.028	490.9	509.3	495.8	503.8	483	505.4	489.3	490.5	429.9	428.2	424.8	406.6	498.03	444.88	0.89
119	Ndufb10	-0.120	0.084	317.95	312.65	317.75	323.15	292.65	327.4	288.1	283.15	296.1	283.2	303.35	286.15	315.26	290.01	0.92
120	Ndufb3	-0.311	0.070	141.5	166.1	162.2	138.8	126.7	183.9	133.2	158.9	117.7	120.5	111.3	99.3	153.20	123.48	0.81
121	Ndufb4	-0.226	0.063	348.9	328.7	328.9	329.2	314.9	373.5	320.8	358.7	270.2	252.2	260.3	268.7	337.35	288.48	0.86
122	Ndufb6	-0.345	0.036	553	675.4	588.2	579.3	473.3	641.5	527.5	560.5	434.5	399	423.1	419.1	585.12	460.62	0.79
123	Ndufb8	-0.664	0.019	179.6	186	193.6	186.8	193	178.1	172.1	164.5	94.1	90.1	99.3	84.9	186.18	117.50	0.63
124	Ndufc2	-0.225	0.026	159.25	171.6	160.85	161.05	158.1	172.5	161.05	165	130.8	130.8	130.75	123.05	163.89	140.24	0.86
125	Ndufv1	-0.167	0.079	124.3	141.5	131.85	128.4	122.2	126.35	124.5	121.1	104	105.15	114.25	120.85	129.10	114.98	0.89
126	Nolc1	-0.353	0.091	194.6	196.4	173.6	197	173.7	208.9	208.4	186.7	158	113.5	131.5	97.7	190.70	149.30	0.78
127	Nop58	-0.466	0.030	122.6	127.6	129.4	112.6	118.2	113	125.1	104.6	71.8	82	73.6	66.6	120.57	87.28	0.72
128	Nt5dc2	-0.285	0.036	245.9	249.3	253.4	241.7	229	252.9	230	254	173	190.8	183.8	176.8	245.37	201.40	0.82
129	Park7	-0.116	0.067	364.6	372.8	344	417.5	377.7	438.2	407.2	396.8	336.4	336.8	340.9	317.8	385.80	355.98	0.92
130	Pdcd5	-0.198	0.026	406.4	450.5	438.9	434.7	432.4	443.7	414.9	428.7	350.8	369.7	355.2	353.5	434.43	378.80	0.87
131	Pdhb	-0.201	0.075	259.5	286.1	267.75	267.75	272.4	236.4	231.6	246.15	240.85	211.05	233.25	220.5	264.98	230.57	0.87
132	Pmpca	-0.238	0.042	130.2	114.9	141.5	124.6	123.9	117.3	124.2	119.2	96	105.4	98.5	94.7	125.40	106.33	0.85
133	Ppia	-0.089	0.014	267.9	269.2	275.7	252.6	239.1	265.1	259.9	261.8	220.5	229.9	253.7	249.7	261.6	245.9	0.94
134	Ppp1r12a	-0.305	0.048	156.3	183.6	168.7	163.9	214.5	193.3	164.9	176.4	122.4	132.9	132.3	145.3	180.05	145.70	0.81
135	Ppp2ca	-0.202	0.038	268.45	240.65	253.5	250.25	257.5	242.15	231.7	228.55	211.95	213.2	212.3	217.35	252.08	219.18	0.87
136	Ppp4r1	-0.694	0.052	158.3	173.3	163.8	185.2	140.2	201.5	162.3	184.1	95.4	69.6	63.2	57.3	170.38	105.32	0.62
137	Pqbp1	-0.996	0.044	213.8	243.3	198.9	233.2	159.4	236.3	193.6	236.3	59.9	48.9	65.4	40.3	214.15	107.40	0.50
138	Prkar1a	-0.338	0.082	213.75	226.55	216.95	206.2	186.75	212.25	209.65	225.3	153.4	139.6	133.8	136.9	210.41	166.44	0.79
139	Psmb1	-0.203	0.074	209.25	208.3	227.65	220.6	219.5	235.6	217.7	224.7	168.6	193.7	175.35	167.15	220.15	191.20	0.87
140	Psmb4	-0.042	0.030	359.05	369.95	368.6	370.05	389.45	398.05	371.7	380.2	357	358.3	368.4	354.9	375.86	365.08	0.97

	Gene Names	log2(FC)	P value	WT 1	WT 2	WT 3	WT 4	WT 5	WT 6	HOM 1	HOM 2	HOM 3	HOM 4	HOM 5	HOM 6	WT Avg	HOM Avg	Fold change
141	Psmb6	-0.043	0.009	288.5	276.95	295.15	285.75	267.9	304.1	297.4	304.65	260.75	267.15	263	275.4	286.4	278.1	0.97
142	Psmd2	-0.026	0.085	130.9	122.7	142.9	125.3	145.4	138.7	138.6	133	115.8	136.1	143.7	124.1	134.32	131.88	0.98
143	Psmd3	-0.311	0.042	311.85	305.7	311.2	320.6	300.75	339.75	310.45	313	220.8	228.9	215.65	234.7	314.98	253.92	0.81
144	Psmg1	-0.248	0.087	243.3	246.1	230.7	276.1	232.4	321.4	249.4	253.1	200.3	192.3	207.3	202.9	258.33	217.55	0.84
145	Puf60	-0.318	0.034	247.9	253.1	261.8	248.1	242.4	264.4	244.1	254.3	168.1	193.2	181.3	176.3	252.95	202.88	0.80
146	Rack1	-0.183	0.030	327.1	371.2	341.3	349.45	306.95	332.65	312.2	341.4	286.85	267.85	294.05	284.05	338.11	297.73	0.88
147	Ranbp1	-0.008	0.028	244.85	249	234	240.75	248.25	242.2	242.6	260	217.85	243.05	239.8	247.3	243.2	241.8	0.99
148	Rap1a	-0.412	0.012	54.8	50.2	56.1	54	66.7	50.3	46.1	44.8	37.5	39	41.4	40.8	55.35	41.60	0.75
149	Rcc2	-0.231	0.041	206.3	210.8	216.5	220.6	182.4	227.7	192.9	209.3	152	184.5	166.9	172	210.72	179.60	0.85
150	Rcn1	-0.234	0.047	249.3	281.8	237.75	258.8	193.55	196.1	225.8	240.4	223.6	183.15	170	161.9	236.22	200.81	0.85
151	Rnh1	-0.304	0.038	452.4	500.7	520	464.7	462.5	471.4	472.1	474.7	325.8	348.4	346.8	357.5	478.62	387.55	0.81
152	Rpa2	-0.466	0.024	346.5	295	336	330.1	314.4	317.5	306.9	300.9	198.1	205	194	199.7	323.25	234.10	0.72
153	Rpl10l	-0.084	0.053	301.8	317.05	324.15	331	326.4	328.2	303.7	300.15	296.55	316.9	302.5	299.1	321.43	303.15	0.94
154	Rpl34	-0.021	0.058	512.1	489.95	473.95	489.95	493.95	502.35	513.2	498.35	487.9	464.7	496.5	459.8	493.71	486.74	0.99
155	Rpl35	-0.270	0.039	128.8	119.7	117.5	118.8	111.7	135.1	116.5	115.1	83.5	84.1	104.1	103.6	121.93	101.15	0.83
156	Rpl35a	-0.085	0.040	170.55	190.25	150.9	165.35	183.05	164.05	193.55	176.6	150.4	147.65	151.6	145.5	170.69	160.88	0.94
157	Rpl7	-0.061	0.010	208.6	248.4	262.6	268.4	233.3	244.3	283.9	261.8	227.3	203.6	216.1	212.6	244.3	234.2	0.96
158	Rplp1	-0.564	0.089	365.1	410.7	367.7	374.7	259.7	437.4	318.6	379.9	199.8	210.1	190.8	199.2	369.22	249.73	0.68
159	Rps15	-0.046	0.001	300.8	299.2	300.45	289.05	316.65	265.75	294.8	288.8	260.3	296	298.75	277.25	295.3	286.0	0.97
160	Rps16	-0.150	0.042	227.85	233.25	214.35	232.2	230.55	219.4	214.85	213.7	187	203.15	196.05	208.8	226.27	203.93	0.90
161	Rps6	-0.176	0.055	244.05	241.25	229	235.25	231.8	234.75	233.9	233.65	194.8	195.3	199.35	196.45	236.02	208.91	0.89
162	Sar1a	-0.412	0.064	208.6	219.2	217.1	223	191.7	253.6	223.9	229.5	134.7	138.9	134.6	125.4	218.87	164.50	0.75
163	Sccpdh	-0.249	0.075	60.6	69.8	75.4	64.7	73.7	74	61.7	76.2	54.7	48.8	53.4	57.1	69.70	58.65	0.84
164	Sdhc	-0.138	0.035	217.7	212.6	204.35	198.05	212.6	206.1	188.55	202.35	169.15	184.85	191.8	200.8	208.57	189.58	0.91
165	Sdhd	-0.418	0.021	349.8	354.1	401.9	298.6	340	369.5	329.3	304.4	233	241	238.5	235.9	352.32	263.68	0.75
166	Sec23a	-0.428	0.024	168.9	195.5	193.5	185	183.4	176	168.3	178	119.7	117.4	116.7	119.1	183.72	136.53	0.74
167	Selenbp2	-0.238	0.047	457.6	425.7	421.2	435.5	369	416.6	423.3	408.5	345.7	318.7	321.6	324.4	420.93	357.03	0.85
168	Sf3b1	-0.409	0.026	196.6	202.1	201.8	189.8	202.6	199.3	193.6	185.5	112.2	135.3	130.3	141	198.70	149.65	0.75
169	Slc1a5	-0.701	0.027	160.6	193.1	193.6	189.9	165.8	197.9	175.1	167.2	84.8	88.7	79.5	81.7	183.48	112.83	0.61
170	Slc25a11	-0.466	0.036	255.4	262.6	296.2	261.4	233.4	267.4	243.4	256.9	165.5	162.4	156.9	156.3	262.73	190.23	0.72
171	Slc25a12	-0.242	0.089	321.8	337.35	354.5	344.15	312.05	349.4	320.35	348.85	250.55	261.2	263.5	262.95	336.54	284.57	0.85
172	Slc25a3	-0.051	0.047	379	380.55	373.4	344.9	365.75	330.15	344.05	335.45	360.25	354.55	343.3	361	362.29	349.77	0.97
173	Slc25a4	-0.160	0.063	238	249.4	230.6	228	241.7	231.5	224.6	235.3	205.2	204.6	200.2	200	236.53	211.65	0.89
174	Slirp	-0.150	0.057	242.3	256.9	236.8	240.7	248.4	257.9	242.5	250.8	212.7	217.8	219.5	193.3	247.17	222.77	0.90
175	Smarcc1	-0.172	0.036	323.9	328.8	325.1	325.4	304.4	298.1	289.5	281.4	275.7	280.8	285.8	278.4	317.62	281.93	0.89

	Gene Names	log2(FC)	P value	WT 1	WT 2	WT 3	WT 4	WT 5	WT 6	HOM 1	HOM 2	HOM 3	HOM 4	HOM 5	HOM 6	WT Avg	HOM Avg	Fold change
**176**	Smg1	-0.315	0.010	233.1	255.7	243.7	220.6	209.9	258.6	193.4	204.9	173.4	194.4	190.5	186.5	236.93	190.52	0.80
**177**	Snrpd1	-0.141	0.089	264.9	274	292.4	257.7	275.1	291.5	266.9	279.2	211.1	260.2	245.8	238.1	275.93	250.22	0.91
**178**	Spr	-0.120	0.042	142.5	147.6	138.7	139.5	146.7	151	142.4	140.3	137.4	123.7	124.4	128.8	144.33	132.83	0.92
**179**	Srsf7	-0.190	0.087	147.75	151.85	145.8	159.1	160.7	172.45	151.5	159.15	119.2	125.3	130.3	136.7	156.28	137.03	0.88
**180**	Synpo2l	-0.519	0.010	252.3	240	252.2	226	248.6	234.55	200.4	216.35	149.25	152.15	148.9	147.55	242.28	169.10	0.70
**181**	Syt4	-0.356	0.080	425.9	421.8	421.1	483.7	446.7	459.8	476.3	467.5	299	289.9	270.4	275	443.17	346.35	0.78
**182**	Tardbp	-0.202	0.085	136.7	199.4	179.8	172.6	153.6	176.2	152.9	196.3	131.2	132.8	128.9	143.3	169.72	147.57	0.87
**183**	Tle5	-1.098	0.037	21.3	20.6	23.4	22.6	16.7	22.1	20.5	21	4	6.9	3.7	3.1	21.12	9.87	0.47
**184**	Tmco1	-0.563	0.040	300.9	298.6	320.4	259.5	252.2	298.4	275.5	298.3	149.2	143.3	147.9	157	288.33	195.20	0.68
**185**	Tmpo	-0.130	0.049	479.3	451.5	441.6	484.6	491.8	467.1	447.7	466.6	379.1	418.3	435.8	426	469.32	428.92	0.91
**186**	Tnnc1	-0.168	0.004	832.05	798.1	846.6	785.55	845.5	769.05	723.15	733.65	700.15	739.75	706.85	738.05	812.8	723.6	0.89
**187**	Tnni1	-0.140	0.045	129.6	143.45	129.9	143.9	133.2	131.85	110.2	130.15	119.7	131.65	115.5	129.5	135.32	122.78	0.91
**188**	Tomm22	-0.181	0.016	227	243.3	241.7	249.5	236	239.5	225.9	227.9	193	201.5	207.6	212	239.50	211.32	0.88
**189**	Tpm4	-0.280	0.064	238.2	193.65	221.05	199.1	213.95	194.55	192.35	180.85	163.6	163.75	172.45	164.85	210.08	172.98	0.82
**190**	Tpt1	-0.038	0.046	285.65	262.95	271.9	265.25	286.45	262.2	241.2	242.8	270.9	270	288.6	278.8	272.40	265.38	0.97
**191**	Trim63	-0.319	0.084	258	283.4	270	268	249.9	279.3	281.9	289.7	185.1	183.3	177.8	171.4	268.10	214.87	0.80
**192**	Tspo	-0.429	0.056	115.9	124.1	135.2	137	115.3	139.1	122.9	137.8	73.2	83.7	80.5	71.3	127.77	94.90	0.74
**193**	Tufm	-0.216	0.039	142.05	140.45	145.1	144.7	152.6	140.5	147.45	159.45	103.85	118.8	105.15	110.55	144.23	124.21	0.86
**194**	U2af65	-0.188	0.089	129.1	151.2	145.1	139.3	145.7	166.5	148.1	146.1	117.7	117.2	116.9	123.7	146.15	128.28	0.88
**195**	Ube2d3	-0.285	0.062	244.8	265	234.8	245.2	243.2	276.8	244.9	268.6	176.9	183.1	187.7	177.9	251.63	206.52	0.82
**196**	Uggt1	-0.257	0.044	263.1	264.5	291	265.2	252.9	249.3	263.6	263.9	196.2	198.6	210.1	194.9	264.33	221.22	0.84
**197**	Uqcr10	-0.897	0.025	208.3	205	173.6	173.4	178.3	184.9	180.6	159.9	77.5	71.8	57.6	55.9	187.25	100.55	0.54
**198**	Uqcr11	-0.614	0.027	66.4	90.5	80	68.4	50.6	56.8	52.2	54.1	36.2	44.6	40.2	42.3	68.78	44.93	0.65
**199**	Uqcrq	-0.417	0.027	211.3	239.5	242.7	274.4	247.7	230.8	228.7	223	166.2	154.8	157.7	152.9	241.07	180.55	0.75
**200**	Vcl	-0.029	0.023	293	282.8	269.6	285.6	275.1	263.3	276.1	299	270.2	255	267.7	268.2	278.2	272.7	0.98
**201**	Vps35	-0.078	0.245	94.45	87.65	88.75	93.85	93.05	87.6	88.55	87.6	80.85	90.6	82.25	86.85	90.89	86.12	0.95
**202**	Wdr1	-0.026	0.040	210.3	229.6	231.1	246.2	211.4	239.6	232.1	245.9	199.2	215.3	215.7	235.9	228.0	224.0	0.98
**203**	Ybx1	-0.294	0.052	86.5	89.5	76.65	118.9	104.3	87.05	108.85	86.6	80.3	67.9	66.55	48.85	93.82	76.51	0.82
**204**	Zadh2	-0.355	0.033	287	296.7	289.7	294.3	266.9	265.1	275.5	275.6	171.7	214.4	195.2	196.1	283.28	221.42	0.78
